# Targeting NRP1 in Endothelial Cells Facilitates the Normalization of Scar Vessels and Prevents Fibrotic Scarring

**DOI:** 10.1002/advs.202510545

**Published:** 2025-12-08

**Authors:** Yu Wang, Xin Zhou, Min Liu, Meimei Huang, Peirong Chen, Xinying Li, Fangchao Xue, Wenyan Zhao, Di Liu, Lang Li, Yuangang Lu, Wen Zeng

**Affiliations:** ^1^ Department of Cell Biology Army Medical University Chongqing 400038 China; ^2^ Jinfeng Laboratory Chongqing 401329 China; ^3^ Department of Plastic & Cosmetic Surgery Daping Hospital Army Medical University Chongqing 400042 China; ^4^ Department of Plastic Surgery Southwest Hospital Army Medical University Chongqing 400038 China; ^5^ Department of Neurology Southwest Hospital Army Medical University Chongqing 400038 China; ^6^ State Key Laboratory of Trauma Burn and Combined Injury Chongqing 400038 China

**Keywords:** endothelial cells, hydrogel, NRP1, skin scars, vessels

## Abstract

Current clinical treatments for skin scars primarily reduce vascular density in situ. But, outcomes remain unsatisfactory due to limited understanding of scar vascular structure, endothelial cell (EC) heterogeneity, and functional changes. Through dermatoscopy, scanning electron microscopy, and immunofluorescence staining, our study revealed substantial vascular remodeling in scars, including increased neovascularization density, branching complexity, and incomplete vascular wall coverage. Single‐cell sequencing constructed an EC atlas of scar patients, identifying upregulated ATP synthesis, decomposition, and oxidative phosphorylation in scar ECs—characteristics resembling tumor vasculature. Notably, a subset of ECs with high neuropilin‐1 (NRP1) expression exhibited mesenchymal characteristics. In vitro experiments demonstrated that NRP1 knockdown blocked the transforming growth factor‐beta (TGF‐β)/SMAD family member 2 (SMAD2) signaling pathway and mitigated endothelial‐to‐mesenchymal transition (EndMT). Importantly, NRP1 inhibition reduced EndMT, restored normal vascular function and structure, and prevented scar formation in mice. Based on these findings, a functional hydrogel spray was developed using an NRP1‐targeting peptide, effectively preventing scar formation by promoting vascular normalization.

## Graphical abstract

Scars exhibit vascular abnormal alterations, including upregulated NRP1 expression in endothelial cells, increased vascular density and branching, compromised vessel wall integrity, and incomplete pericyte coverage. Therapeutic targeting of NRP1 through hydrogel spray delivery offers a promising approach to normalize aberrant vasculature and prevent fibrotic scarring.

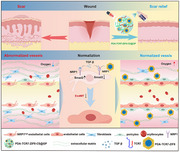

## 1 Introduction

Skin scar is an inevitable result of adult injury, which is characterized by excessive extracellular matrix deposition. Scar exhibits notable changes in appearance, shape, structure, and function. Clinical management for scars realize their effects by reducing the density of blood vessels, such as radiotherapy, local pressure therapy, injection therapy, and laser therapy. However, these approaches have limited efficacy and significant adverse reactions.^[^ ^1^ ^]^ Bevacizumab injection could reduce scar volume. While, its efficacy is influenced by VEGF expression levels, disease duration, and genetic heterogeneity. Furthermore, it may impair physiological angiogenesis and delay wound healing.^[^ ^2^ ^]^ Pulsed dye laser (PDL) is also commonly used for scar treatment. PDL has an efficiency of ≈70%. It could significantly reduce scar erythema and thickness. However, its efficacy is closely related to the vascular density, disease duration, and tissue thickness. In addition, these treatments may cause vascular abnormalities, skin atrophy and exacerbate scar.^[^ ^3^ ^]^ The current treatments cannot directly target the abnormal blood vessels in scars. Limited efficiency and adverse reactions are usually accompanied by these treatments. And no effective prevention for scar exists.^[^ ^4^ ^]^ Therefore, further exploring the characteristics of blood vessels and ECs in scars is necessary. This could help us to identify key targets in scar and develop more precise preventing strategies.

Fibrosis is a notable characteristic of scars. Several research have confirmed that abnormal blood vessel formation and dysfunction are important to fibrosis.^[^ ^5^ ^]^ For instance, the capillarization of hepatic sinusoids can promote liver fibrosis. While, the neovascularization of portal vessels can inhibit liver fibrosis.^[^ ^6^ ^]^ With the extensive application of single‐cell sequencing, the heterogeneity of ECs has been found in different organs and tissues.^[^ ^7^, ^8^, ^9^, ^10^, ^11^ ^]^ The disruption of EC functions can lead to dysfunctions, including impaired tissue healing, thrombosis, excessive inflammation and fibrosis.^[^ ^12^ ^]^ However, existing studies mainly focus on the role of fibroblast heterogeneity in skin scar formation.^[^ ^13^, ^14^ ^]^ So, understanding the characteristics of ECs in scars and identifying key targets are important for making therapies more accurate and effective.

Neuropilin1(NRP1) is a single‐pass transmembrane, non‐tyrosine kinase surface glycoprotein that exists in all vertebrates and is highly conserved. NRP1 is a pleiotropic co‐receptor for multiple growth factors, including VEGF‐A, FGF, and HGF.^[^ ^15^ ^]^ It is widely expressed on ECs and various tumor cells. High expression of NRP1 in tumor tissues can promote the formation of neovascularization and vasculogenic mimicry, leading to abnormal changes in tumor blood vessels.^[^ ^16^, ^17^, ^18^ ^]^ Transgenic mice with systemic NRP1 over‐exhibit increased capillary density yet suffer from severe hemorrhage.^[^ ^19^ ^]^ Moreover, NRP1 promotes VEGF‐ and ECM‐induced endothelial migration^[^ ^20^, ^21^ ^]^ and plays a pivotal role in both physiological and pathological angiogenesis.^[^ ^22^ ^]^ Meanwhile, NRP1 is also a unique immunomodulator in cancer immunotherapy. NRP1 regulates the function of Treg cells and CD8^+^T cells, which together hinder anti‐tumor immunity.^[^ ^23^, ^24^ ^]^ Additionally, targeted knockout of NRP1 in ECs suppresses fibrosis in multiple aging organs by the platelet‐macrophage‐mediated “circulatory microenvironment”.^[^ ^25^ ^]^ Also, NRP1 is involved in the remodeling process of cardiac fibrosis caused by hypertension.^[^ ^26^ ^]^ In pancreatic ductal adenocarcinoma, NRP1 is significantly associated with tumor‐associated endothelial‐to‐mesenchymal transition (EndMT), and reducing NRP1 expression attenuates EndMT and fibrosis.^[^ ^27^ ^]^ These studies suggest that NRP1 plays a crucial role in both abnormal angiogenesis and fibrotic diseases. However, the involvement of NRP1 in scar‐related EndMT remains unexplored.

In our study, we investigated how blood vessels and ECs change in scar tissue. Different methods were applied in our research including dermatoscopy, scanning electron microscopy (SEM), single cell sequencing data analysis, and immunofluorescence staining. By in‐depth analysis of single cell sequencing data, a special group of ECs with high *NRP1* expression (*NRP1* ^high^ ECs) was discovered. These cells were particularly activated in scars. Building on this discovery, we designed a hydrogel spray by an NRP1‐targeting peptide. Our research not only uncovered the key alterations in blood vessels and ECs within scars but also demonstrated their crucial roles in scar pathogenesis.

## 2 Results

### 2.1 Alterations of Blood Vessels and ECs in Scars

**Figure 1:**
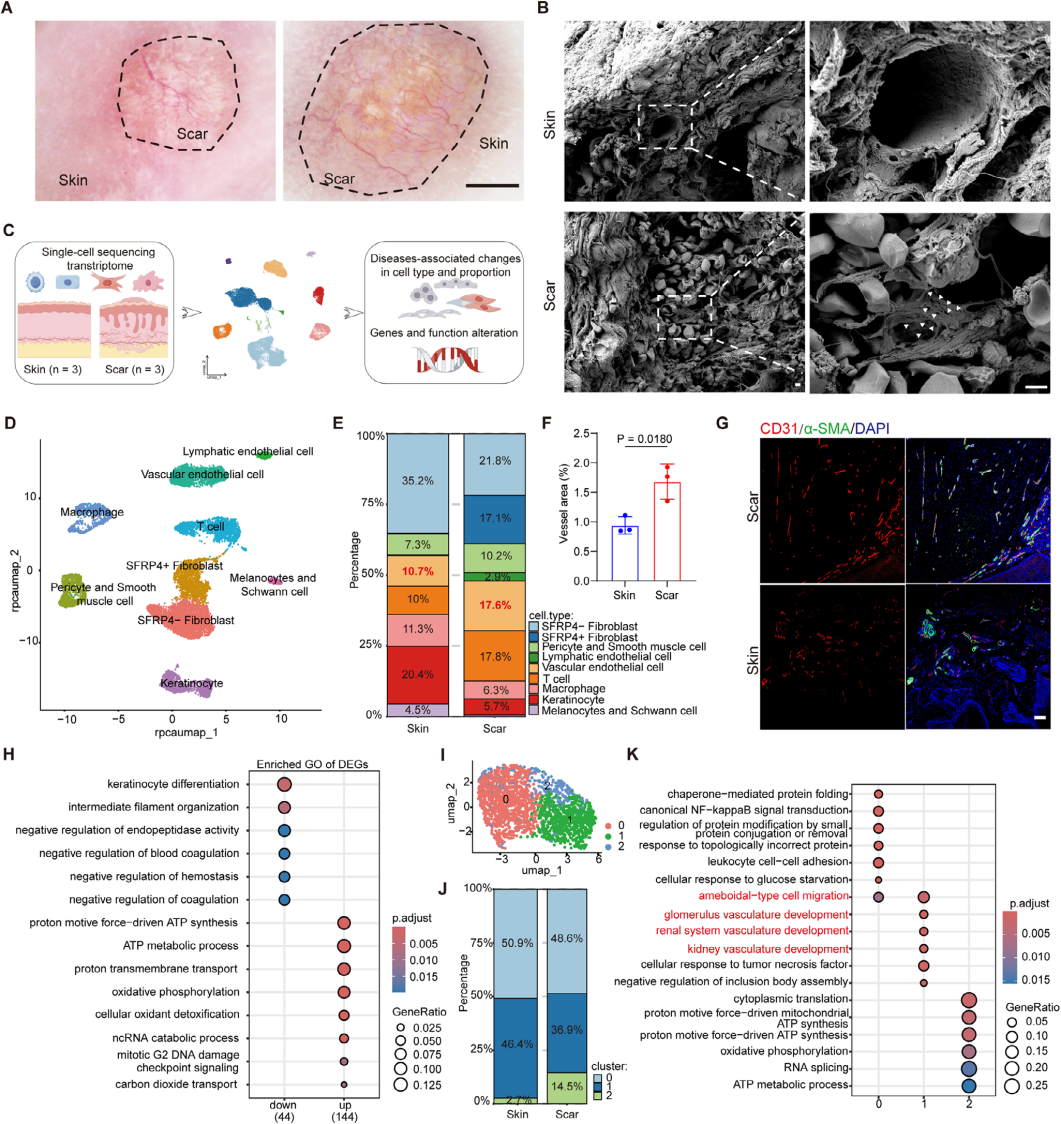
Abnormal changes of blood vessel and ECs in human scar tissues. A) Representative images of scar and normal skin under dermatoscopy. The black dotted lines indicate the area of scar. Scale bars, 2 mm. B) Representative SEM images of vessels in normal skin and scar. The white triangles indicate the discontinuation in the vessel's wall. Scale bars, 1 µm. C) Schematic diagram of single‐cell transcriptome analysis from three scar samples and three normal skin samples. D) UMAP (UMAP, Uniform Manifold Approximation and Projection) visualization of cell type (*n* = 3). E) Proportion of different cells in scar tissues and normal skin. The proportion of ECs is marked in red font (*n* = 3). F‐G) Representative immunofluorescent images and quantification of vessels in normal skin and scar. CD31(red), α‐SMA (green), DAPI (blue). (*n* = 3). Scale bars, 100 µm. H) Functional enrichment analysis of DEGs in scar ECs (*n* = 3). I) UMAP visualization shows 3 subclusters of ECs in normal skin and scar (*n* = 3). J) Bar charts showing the proportion of EC subclusters in scar and normal skin (*n* = 3). K) Functional enrichment analysis of DEGs in different EC subclusters in scar (*n* = 3). Statistical significance was analyzed by unpaired two‐tailed Student's *t*‐test.

Dermatoscopy examination of patients revealed loss of normal skin texture in scar tissues, accompanied by prominent collagen deposition and pigmentation. Scar tissues exhibited significantly increased vascular density, branching, and dilation, displaying characteristic dendritic vascular patterns (**Figure** **1**A). SEM identified vessels in scar are lined with an irregular, chaotically organized and discontinuous layer of ECs (Figure 1B). To delve into the characteristics of vascular ECs in scars, we analyzed the single cell sequencing data of three normal skin samples and three scar tissue samples (Figure 1C; Table S1, Supporting Information). After data preprocessing and batch effect removal (Figures S1–S3, Supporting Information), the cells were clustered into nine distinct populations (Figure 1D) by cell‐type‐specific markers (Figure S4A, Supporting Information) in both normal skin and scar tissues. As shown by the proportion plot, ECs constituted 10.7% in normal skin, increasing to 17.6% in scar tissues (Figure 1E). Immunofluorescence staining confirmed vascular increments in human scars, characterized by compressed morphology and incomplete pericyte coverage (Figure 1F,G; Figure S4C, Supporting Information). Comparative analysis of ECs from normal and scar tissues revealed 533 differentially expressed genes (DEGs) significantly upregulated and 396 DEGs significantly downregulated in scar ECs (Figure S4B, Supporting Information), indicating distinct transcriptional reprogramming in scar ECs. Functional enrichment analysis further revealed that these upregulated DEGs were involved in ATP synthesis, ATP metabolic process, oxidative phosphorylation, and oxidant detoxification, suggesting scar ECs are in a metabolically stressed state.^[^ ^28^ ^]^ These downregulated DEGs were involved in keratinocyte differentiation and negative regulation of coagulation, indicating scar ECs affect the formation of epidermis and the function of blood vessels (Figure 1H).

Skin ECs were further classified into three subclusters (Figure 1I). The subcluster 0 did not change significantly in proportion. While the proportion of subcluster 1 decreased from 46.4% to 36.9%, and subcluster 2 increased from 2.7% to 14.5% (Figure 1J; Figure S4D, Supporting Information). The subcluster 0 corresponds to capillary endothelium, subcluster 1 to venous endothelium, and subcluster 2 to arterial endothelium (Figure S4F,G, Supporting Information). Functional enrichment analysis was conducted on these subclusters. In subcluster 0, leukocyte adhesion and NF‐kB signaling pathway were enriched. In subcluster 1, cell migration and vascular development were enriched. In subcluster 2, ATP synthesis and oxidative phosphorylation were enriched (Figure 1J). Consequently, we found that in scar tissue, blood vessels increase in number and branching, with their inner walls showing obvious defects and incomplete pericyte coverage. Moreover, the increased quantity and significant functional changes of ECs in scars suggest their crucial role in scar pathogenesis.

### 2.2 The *NRP1* ^high^ ECs Increased in Scars

**Figure 2:**
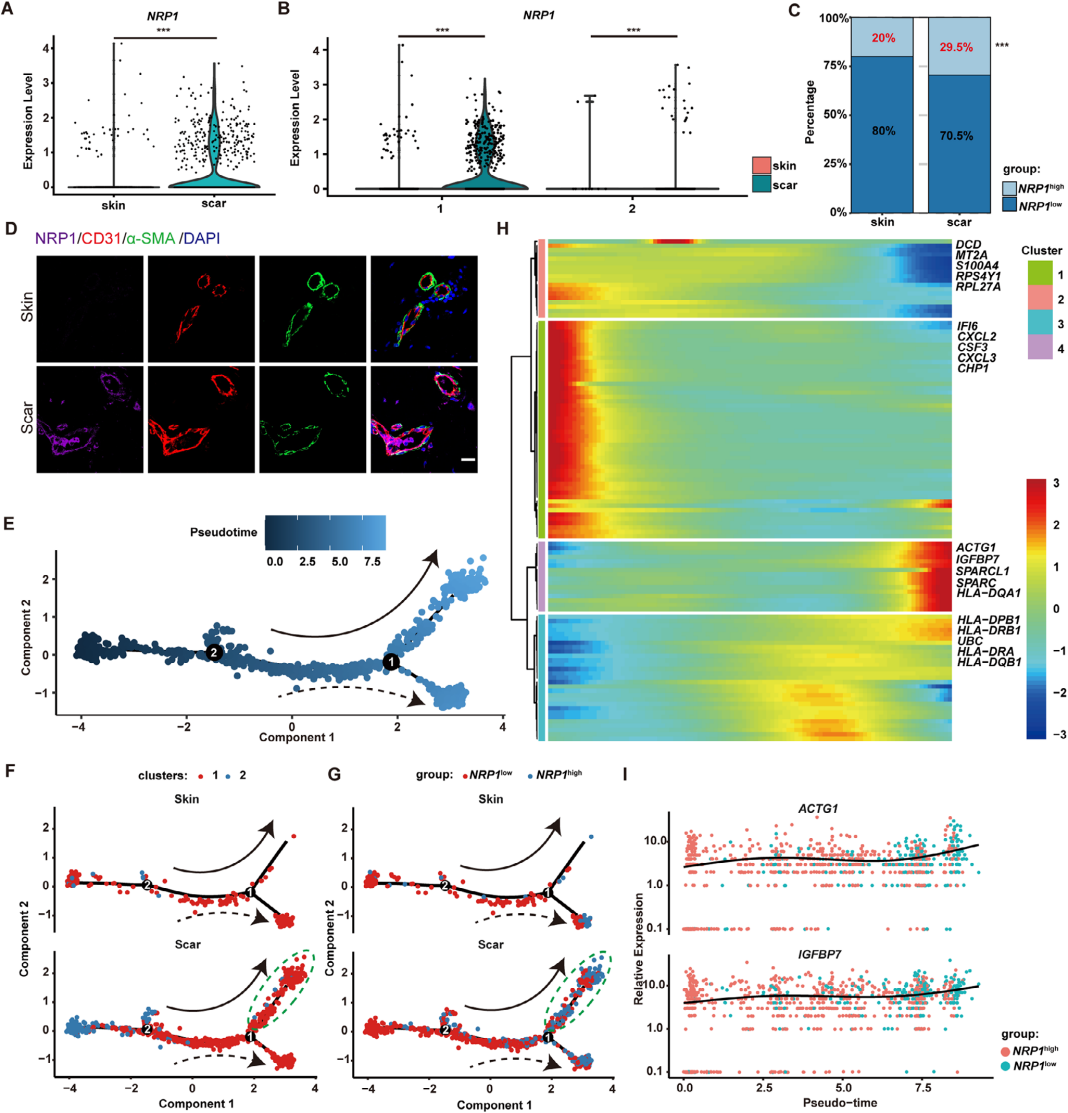
*NRP1* ^high^ECs increased in human scar tissues. A) Expression of *NRP1* between normal skin ECs and scar ECs (*n* = 3). ^***^ *P*< 0.001. B) Expression of *NRP1* in different subcluster ECs between normal skin and scar (*n* = 3). ^***^ *P*< 0.001. C) Proportion of *NRP1* ^high^ ECs and *NRP1* ^low^ ECs in normal skin and scar (*n* = 3). ^***^ *P*< 0.001. D) Immunofluorescent staining of NRP1(purple), CD31(red), α‐SMA (green), DAPI (blue). Scale bars, 20 µm. E) Cell was ordered by pseudotime. The solid and dashed lines represent the directions of different trajectory. F) Pseudotime trajectory of ECs in normal skin and scar. The green dotted line indicates the branch specially activated in scar. G) Pseudotime trajectory of *NRP1* ^high^ ECs and *NRP1* ^low^ ECs in normal skin and scar. The green dotted line indicates the branch specially activated in scar. H) Heatmap of DEGs across pseudotime. Rows were grouped based on similarity of gene expression, resulting in the 4 clusters indicated at the left. The color bar indicates the relative expression level. The top 5 DEGs of every cluster were marked on the right. I) Expression values of *ACTG1* and *IGFBP7* along the pseudotime axis. Statistical significance was analyzed by unpaired two‐tailed Student's *t*‐test.

Abnormal alterations of vessels and ECs in scar exhibiting high resemblance to tumor, neovascularization in both cases derive from angiogenesis.^[^ ^29^, ^30^ ^]^ Through analysis, *NRP1* was significantly upregulated in scar ECs (**Figure** **2**A). The upregulation of *NRP1* was mainly concentrated in the subcluster 1 of scar ECs (Figure 2B), which was closely associated with the vascular development in scars. Then, we classified ECs into *NRP1* ^high^ and *NRP1* ^low^ subclusters based on *NRP1* expression levels. The proportion of *NRP1* ^high^ ECs increased by 9.5% in the scar tissue (Figure 2C). Immunofluorescence images also confirmed the increased expression of *NRP1* in scar ECs (Figure 2D). Functional enrichment analysis revealed that the upregulated DEGs in *NRP1* ^high^ ECs were mainly enriched in the antigen processing and presentation process (Figure S5A, Supporting Information).

Then, we extracted ECs from normal skin and scar, performing pseudotime analysis.^[^ ^31^ ^]^ As the pseudotime trajectory showed (Figure 2E), there were 2 diverging cell states, state 1 on the upper branch and state 2 on the lower branch. In normal skin, most ECs diverged to the lower branch. Interestingly, most scar ECs diverged to the upper branch (Figure 2F), which indicates that this subset of ECs is specifically activated in scars. We further performed pseudotime analysis on *NRP1* ^high^ and *NRP1* ^low^ ECs. And the specifically activated subset of ECs in scars was predominantly *NRP1* ^high^ ECs (Figure 2G).

To elucidate the gene expression dynamics along the differentiation trajectory between *NRP1* ^low^ ECs and *NRP1* ^high^ ECs in scars, we conducted a hierarchical clustering analysis of identified DEGs across pseudotime. The DEGs were further classified into four clusters (Figure 2H).^[^ ^32^ ^]^ The DEGs of cluster 4 were upregulated during the transformation of *NRP1* ^low^ ECs into *NRP1* ^high^ ECs. These DEGs were mainly related to functions such as response to steroid hormones, protein folding, and transepithelial transport (Figure S5C, Supporting Information). Among these DEGs, *ACTG1*, *IGFBP7, SPARCL1* and *SPARC* show the most significant changes (Figure 2I). These genes are scarcely expressed in normal blood vessels but highly expressed in tumor blood vessels, where they promote tumor angiogenesis and are closely associated with epithelial‐to‐mesenchymal transitions (EMTs).^[^ ^33^, ^34^, ^35^, ^36^, ^37^ ^]^ In summary, we have identified the *NRP1* ^high^ ECs specifically activated in scar tissues. During the transformation from *NRP1* ^low^ to *NRP1* ^high^ ECs in scars, accompanied by upregulation of genes associated with tumor angiogenesis and EMT. We hypothesized that a correlation may exist between the *NRP1* ^high^ ECs and abnormal vascular changes in scars.

### 2.3 The *NRP1* ^high^ ECs with Mesenchymal Characteristics

**Figure 3:**
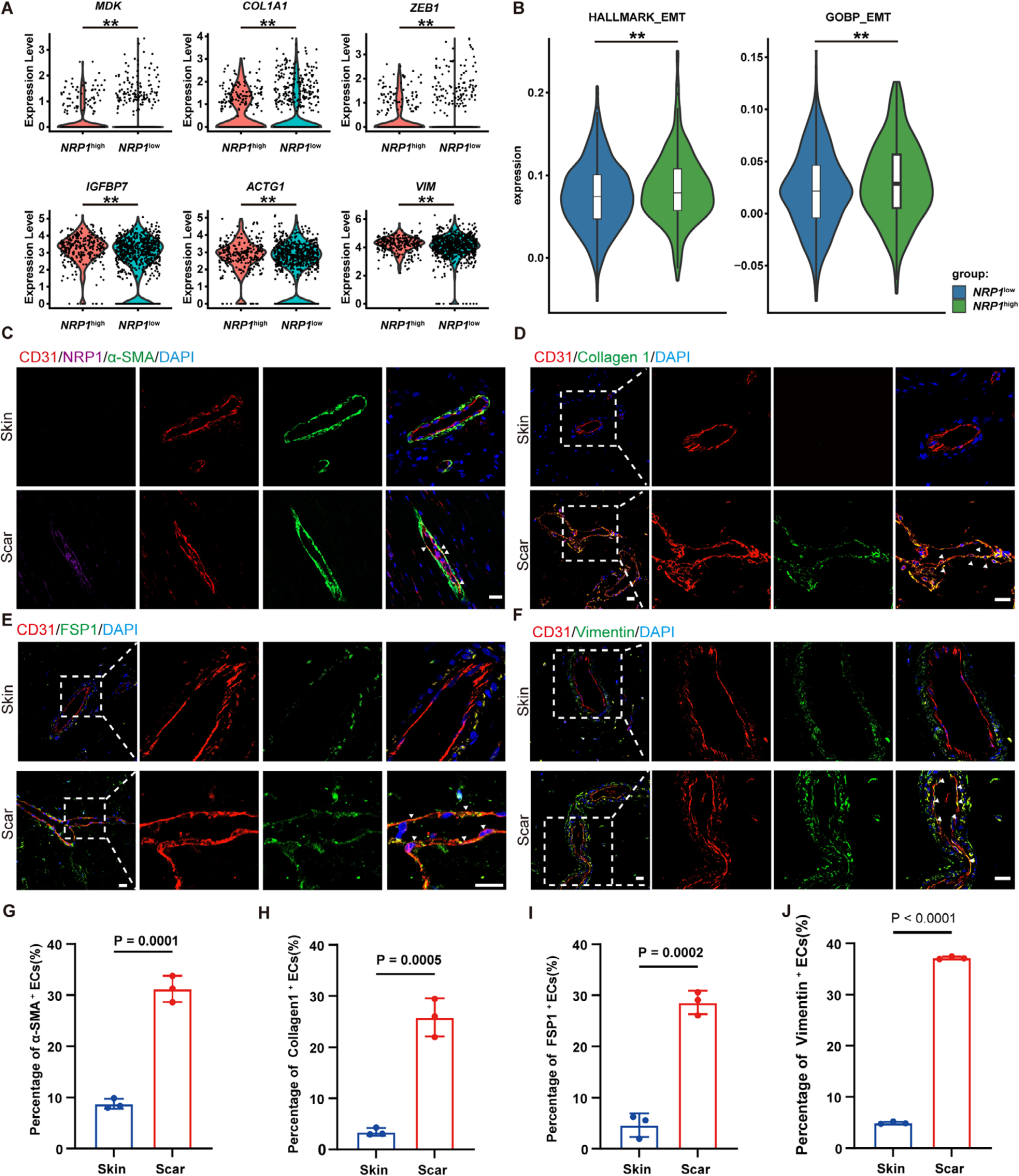
The *NRP1* ^high^ ECs exhibit mesenchymal characteristics. A) Violin plots show the expression of EMT‐related genes in *NRP1* ^high^ ECs and *NRP1* ^low^ ECs (*n* = 3). ^**^ *P* < 0.01. B) Violin plots present the HALLMARK and GOBP functional scoring analysis for EMT‐related genes in *NRP1* ^high^ ECs and *NRP1* ^low^ ECs (*n* = 3). ^**^ *P* < 0.01. C) Immunofluorescent staining of α‐SMA (green), CD31(red), NRP1(purple), DAPI (blue) in human normal skin and scar tissue. The triangles indicate the ECs expressed with NRP1 and α‐SMA. Scale bars, 20 µm. D) Immunofluorescent staining of Collagen I (green), CD31(red), DAPI (blue) in human normal skin and scar tissue. The white dotted lines indicate vessels, and the triangles indicate the ECs expressed with Collagen I. Scale bars, 20 µm. E) Immunofluorescent staining of FSP1 (green), CD31(red), DAPI (blue) in human normal skin and scar tissue. The white dotted lines indicate vessels, and the triangles indicate the ECs expressed with FSP1. Scale bars, 20 µm. F) Immunofluorescent staining of Vimentin(green), CD31(red), DAPI (blue) in human normal skin and scar tissue. The white dotted lines indicate vessels, and the triangles indicate the ECs expressed with Vimentin. Scale bars, 20 µm. G‐J) Quantification of the percentage of α‐SMA^+^, Collagen I^+^, FSP1^+^, Vimentin ^+^ ECs in human scar tissue (*n* = 3). Statistical significance was analyzed by unpaired two‐tailed Student's *t*‐test.

To explore the functional characteristics of the *NRP1* ^high^ ECs, we further analyzed the DEGs between the *NRP1* ^high^ and *NRP1* ^low^ ECs. Genes such as *IGFBP7, ZEB1, MDK, COL1A1, ACTG1*, and *VIM*, which play crucial roles in EMT, were significantly upregulated in *NRP1* ^high^ ECs (**Figure** **3**A). Subsequently, based on the EMT related gene sets (Tables S7 and S8, Supporting Information), we conducted HALLMARK and GOBP functional enrichment analyses on the DEGs between the *NRP1* ^high^ and *NRP1* ^low^ ECs. The results of both analyses showed that the levels of EMT related genes in *NRP1* ^high^ ECs were significantly higher than *NRP1* ^low^ ECs (Figure 3B). Immunofluorescence staining results confirmed that in human scar tissue, ECs with high NRP1 expression also expressed α‐SMA (Figure 3C). Additionally, the percentage of scar ECs expressed mesenchymal markers such as α‐SMA, fibroblast specific protein 1 (FSP1), Collagen I, and Vimentin significantly increased (Figure 3C–J), while the expression of vascular endothelial cadherin (VE‐cadherin) decreased (Figure S5D, Supporting Information). The above results indicate that the *NRP1* ^high^ ECs exhibit distinct mesenchymal characteristics. And the proportion of mesenchymal ECs significantly increased in scar tissue.

### 2.4 TGF‐β Upregulates NRP1 in ECs and Promotes EndMT Through the SMAD2 Signaling Pathway

**Figure 4:**
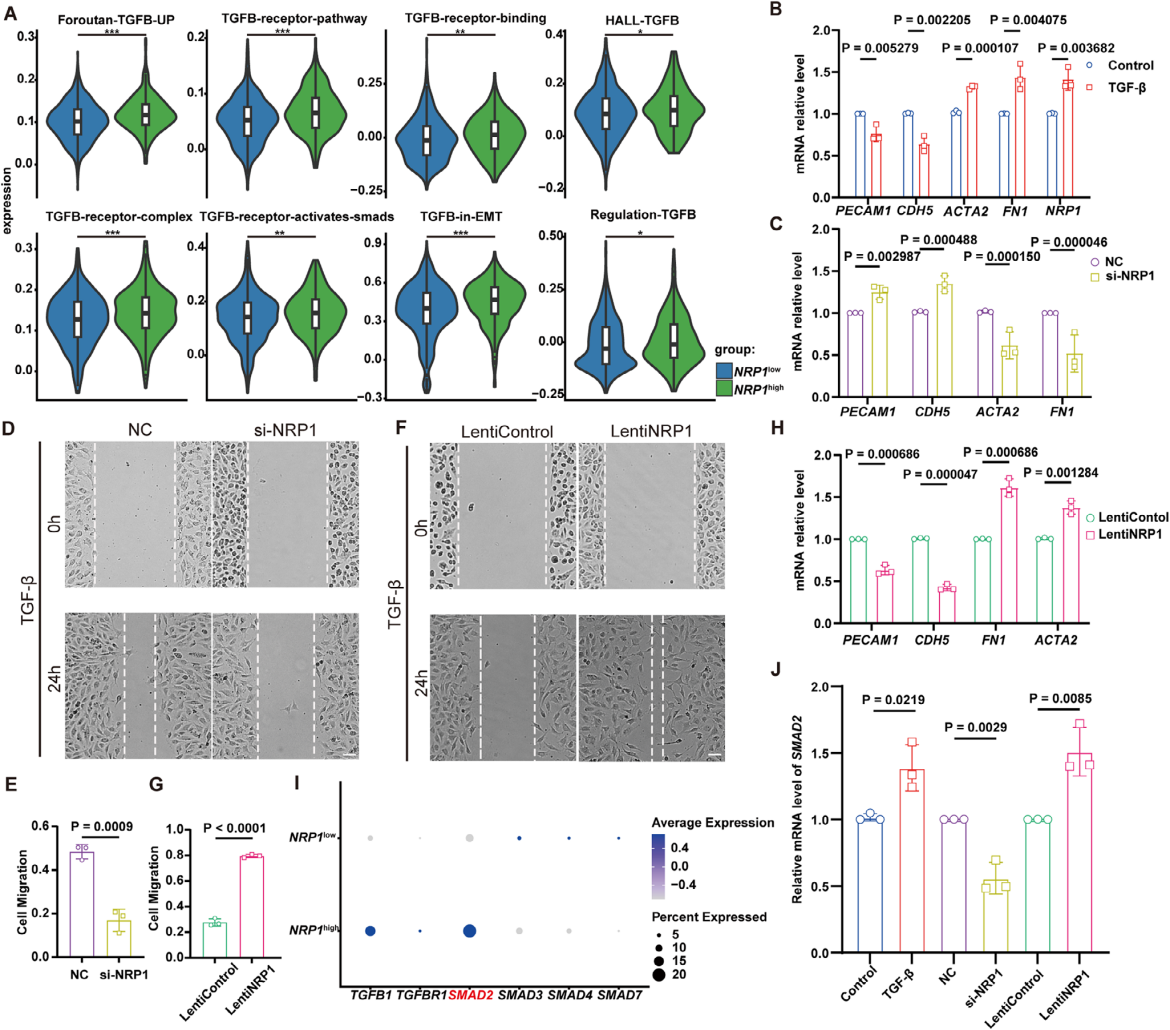
TGF‐β upregulate NRP1 in ECs and promote EndMT by SMAD2. A) Violin plots showing the expression of TGF‐β associated signal pathway related in *NRP1* ^high^ ECs and *NRP1* ^low^ ECs. B) Detection of transcript levels of specific markers associated with EndMT and *NRP1* in HUVECs treated with TGF‐β (*n* = 3). C) Detection of transcript levels of EndMT associated markers in HUVECs treated with TGF‐β and transfected with si‐NRP1 (*n* = 3). D‐E) Representative images and quantification of the migration in HUVECs treated with TGF‐β and transfected with si‐NRP1 (*n* = 3). F‐G) Representative images and quantification of the migration in HUVECs overexpressed with NRP1 and treated with TGF‐β (*n* = 3). H) Detecting transcript levels of EndMT associated markers in HUVECs overexpressed with NRP1 and treated with TGF‐β (*n* = 3). I) Expression level of SMAD family molecules in scar *NRP1* ^high^ECs and *NRP1* ^low^ECs. J) Detecting transcript levels of *SMAD2* in HUVECs treated with TGF‐β, treated with TGF‐β and transfected with si‐NRP1, overexpressed with NRP1 and treated with TGF‐β (*n* = 3). Statistical significance was analyzed by unpaired two‐tailed Student's *t*‐test.

To identify the upstream regulator of NRP1, we compared the transcriptional profiles between *NRP1* ^high^ and *NRP1* ^low^ ECs. In scar *NRP1* ^high^ ECs, the different TGF‐β signal pathways in GOBP and HALLMARK gene sets were significantly activated (**Figure** **4**A). Thus, we treated human umbilical vein endothelial cells (HUVECs) with TGF‐β, which led to a significant upregulation of *NRP1* and mesenchymal markers *ACTA2* and *FN1*, downregulated the expressions of endothelial markers *PECAM1* and *CDH5* (Figure 4B; Figure S6A, Supporting Information). This suggests that NRP1 may be involved in TGF‐β induced EndMT. EndMT refers to the process in which ECs lose their endothelial characteristics and transform into mesenchymal cells under stimulus like TGF‐β and oxidative stress.^[^ ^38^ ^]^ EndMT is a key factor leading to abnormal changes in tumor vessels and plays an important role in fibrosis.^[^ ^39^, ^40^, ^41^ ^]^

We wanted to figure out if NRP1 is involved in TGF‐β induced EndMT. For this, we used siRNA to knock down NRP1. A lentivirus was used to overexpress it in HUVECs. Our result showed that si‐NRP1 transfection reduced the expression of *NRP1*, *ACTA2* and *FN1*, but increased *PECAM1* and *CDH5* in TGF‐β treated HUVECs (Figure 4C; Figure S6B,C, Supporting Information). This suggests that si‐NRP1 inhibits EndMT. Knocking down NRP1 reduces both EC migration and tube formation (Figure 4D,E; Figure S6D,E, Supporting Information). While overexpressing NRP1 promoted EC migration and angiogenesis (Figure 4F,G; Figure S6F–H, Supporting Information). It also decreased the expression of *PECAM1* and *CDH5*, while upregulating *ACTA2* and *FN1* in TGF‐β treated HUVECs (Figure 4H; Figure S6I, Supporting Information). These results indicate that TGF‐β upregulates NRP1 and promotes EndMT.

The TGF‐β/SMAD signaling pathway is key to the EndMT.^[^ ^42^ ^]^ We compared the expression of SMAD family molecules in *NRP1* ^high^ and *NRP1* ^low^ ECs and found that *SMAD2* levels were higher in *NRP1* ^high^ ECs. This suggests that NRP1 may regulate EndMT via the TGF‐β/SMAD2 pathway (Figure 4I). Our in vitro experiments confirmed that TGF‐β upregulates *SMAD2* expression in ECs. Knocking down NRP1 reduced *SMAD2* expression, while overexpressing NRP1 increased it (Figure 4J; Figure S6A,C,I, Supporting Information). To further investigate the mechanisms, we used SB431542 to inhibit SMAD2 signal pathway. SB431542 could significantly decrease NRP1 expression and attenuate mesenchymal transition under TGF‐β induction (Figure S7A,B, Supporting Information). Moreover, it could also suppress mesenchymal transition in NRP1‐overexpressing cells (Figure S7C,D, Supporting Information). In summary, our results show that TGF‐β upregulates NRP1 through SMAD2 signal, which in turn promotes EndMT through SMAD2 signal. There may be a positive‐feedback loop between NRP1 and TGF‐β/SMAD2 signaling pathway in EndMT.

### 2.5 Inhibition of NRP1 Restores Vessels and Impairs EndMT in Wounds

**Figure 5:**
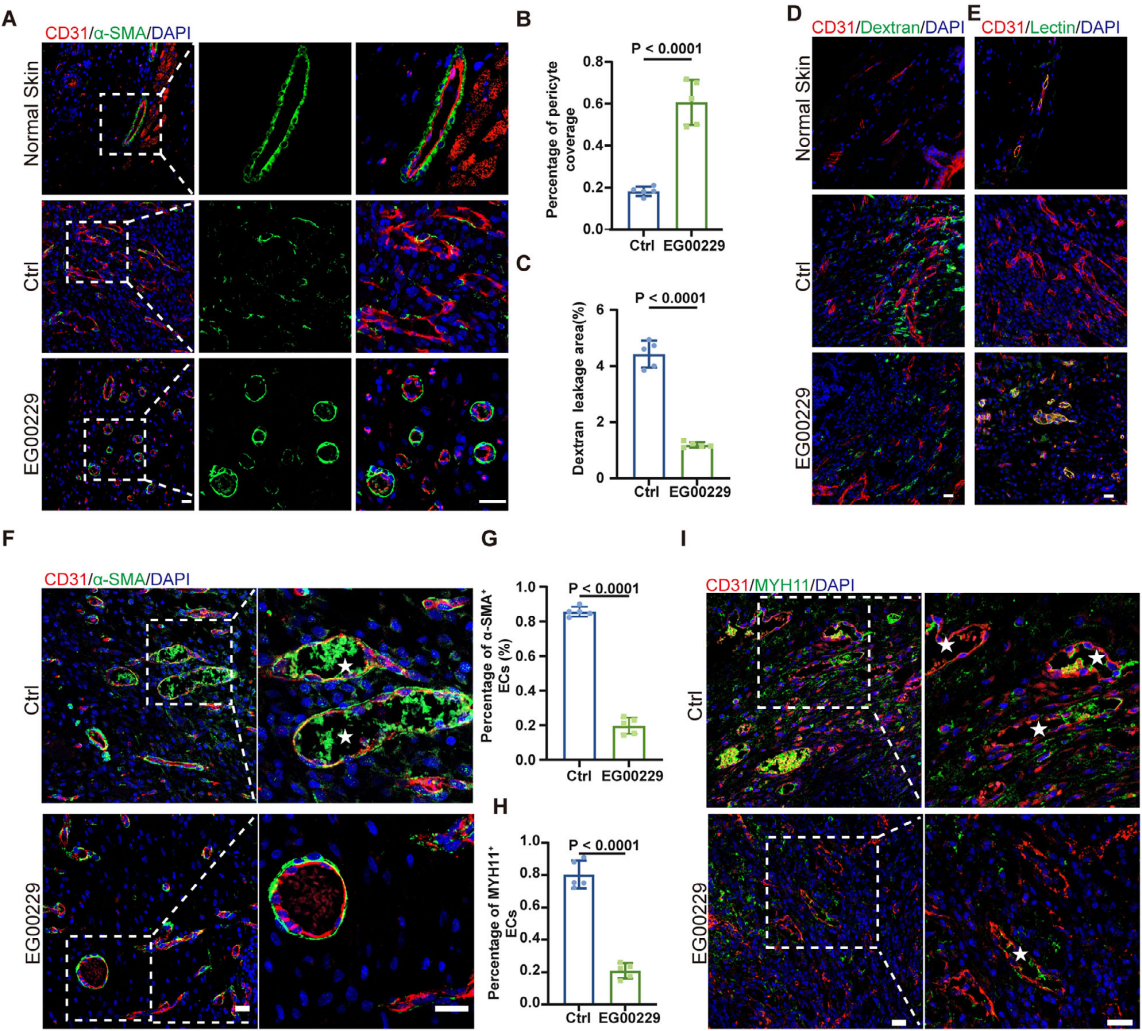
Inhibition of NRP1 impairs EndMT and normalizes the vessels in wound A,B) Immunofluorescent image and quantification of pericyte coverage of vessels, in mice normal skin and wound tissue treated with EG00229 or vehicle Ctrl on the 7th day (*n* = 5). α‐SMA (green), CD31(red), DAPI (blue). The white dotted lines indicate the area of vessels. Scale bars, 20 µm. C,D) Immunofluorescent image and quantification of Dextran leakage area in mice normal skin and wound tissue treated with EG00229 or Ctrl on the 7th day (*n* = 5). Dextran(green), CD31(red), DAPI (blue). Scale bars, 20 µm. E) Immunofluorescent image of Lectin perfused vessels in mice normal skin and wound tissue treated with EG00229 or Ctrl on the 7th day. Lectin(green), CD31(red), DAPI (blue). Scale bars, 20 µm. F,G) Immunofluorescent images and quantification of percentage of α‐SMA^+^ ECs in mice wound tissue treated with EG00229 or Ctrl on the 14^th^ day (*n* = 5). α‐SMA (green), CD31(red), DAPI (blue). The white dotted lines indicate vessels, and the stars indicate the vessels expressed with α‐SMA. Scale bars, 20 µm. H,I) Immunofluorescent images and quantification of percentage of MYH11^+^ ECs in mice wound tissue treated with EG00229 or Ctrl on the 14th day (*n* = 5). MYH11(green), CD31(red), DAPI (blue). The white dotted lines indicate vessels, and the stars indicate the vessels expressed with MYH11. Scale bars, 20 µm. Statistical significance was analyzed by an unpaired two‐tailed Student's *t*‐test.

We wanted to verify if NRP1 is involved in abnormal vascular changes within the wounds of mice scar models. So, we analyzed the vascular structure and function in normal skin, wounds, and wounds treated with an NRP1 antagonist, EG00229.^[^ ^43^ ^]^ Inhibiting NRP1 really made a difference. It significantly decreased the number of blood vessels and branches in mouse wounds (Figure S8A,B, Supporting Information). This indicates that NRP1 plays a role in the excessive angiogenesis of wounds. We further explore the impact of NRP1 inhibition on the vascular structure in wounds. α‐SMA was used to label pericytes.^[^ ^44^ ^]^ In normal mouse skin, ECs were fully covered by pericytes. But in wound tissue, blood vessels had poor pericyte coverage. Interestingly, NRP1 inhibition could increase the pericyte coverage of vessels in wounds (**Figure** **5**A,B). We also used SEM to detect blood vessels in wounds. Blood vessels in wounds had rough and defective inner walls. Inhibiting NRP1 could make the inner wall smoother and intact (Figure S8C, Supporting Information). Our findings confirm that mouse wound vasculature undergoes abnormal changes. And inhibiting NRP1 can restore the vascular structure in wounds.

Next, we investigated the effect of NRP1 inhibition on the function of wound vessels. FITC‐Dextran was injected to assess vascular permeability.^[^ ^45^ ^]^ In normal mouse skin, dextran did not leak into the tissue interstitium. However, a large amount of Dextran was observed in the wound tissue interstitium, suggesting the permeability of wound vessels was increased. While NRP1 inhibition can significantly decrease the permeability of wound vessels (Figure 5C,D). The perfusion function of vessels was evaluated by FITC‐Lectin.^[^ ^46^ ^]^ In normal skin, vessels were labeled by lectin. Conversely, in wound tissue, only a small proportion of vessels were labeled by Lectin. NRP1 inhibition significantly increased this proportion, confirming that blood vessels in wounds lose normal perfusion function, and NRP1 inhibition can remarkably improve it (Figure 5E; Figure S8D, Supporting Information). Moreover, abnormal changes in wound vessels lead to tissue hypoxia, and NRP1 inhibition significantly alleviated hypoxia (Figure S8E,F, Supporting Information). In summary, we found that the structure and function of wound vessels are abnormally altered, and targeting NRP1 can promote the normalization of vessels. Combined with previous research, to further confirm that NRP1 inhibition promotes the normalization of wound blood vessels by reducing EndMT, we examined the impact of NRP1 inhibition on EndMT in mouse scars. Inhibiting NRP1 significantly decreased the proportion of vessels expressing mesenchymal‐related markers, α‐SMA, myosin heavy chain 11(MYH11), FSP1 in wound (Figure 5F–I; Figure S8G,H, Supporting Information). The above studies further validate that NRP1 triggers abnormal alterations in the function and structure of blood vessels within wounds via EndMT.

### 2.6 NRP1 Inhibition Significantly Prevents Scar Formation

**Figure 6:**
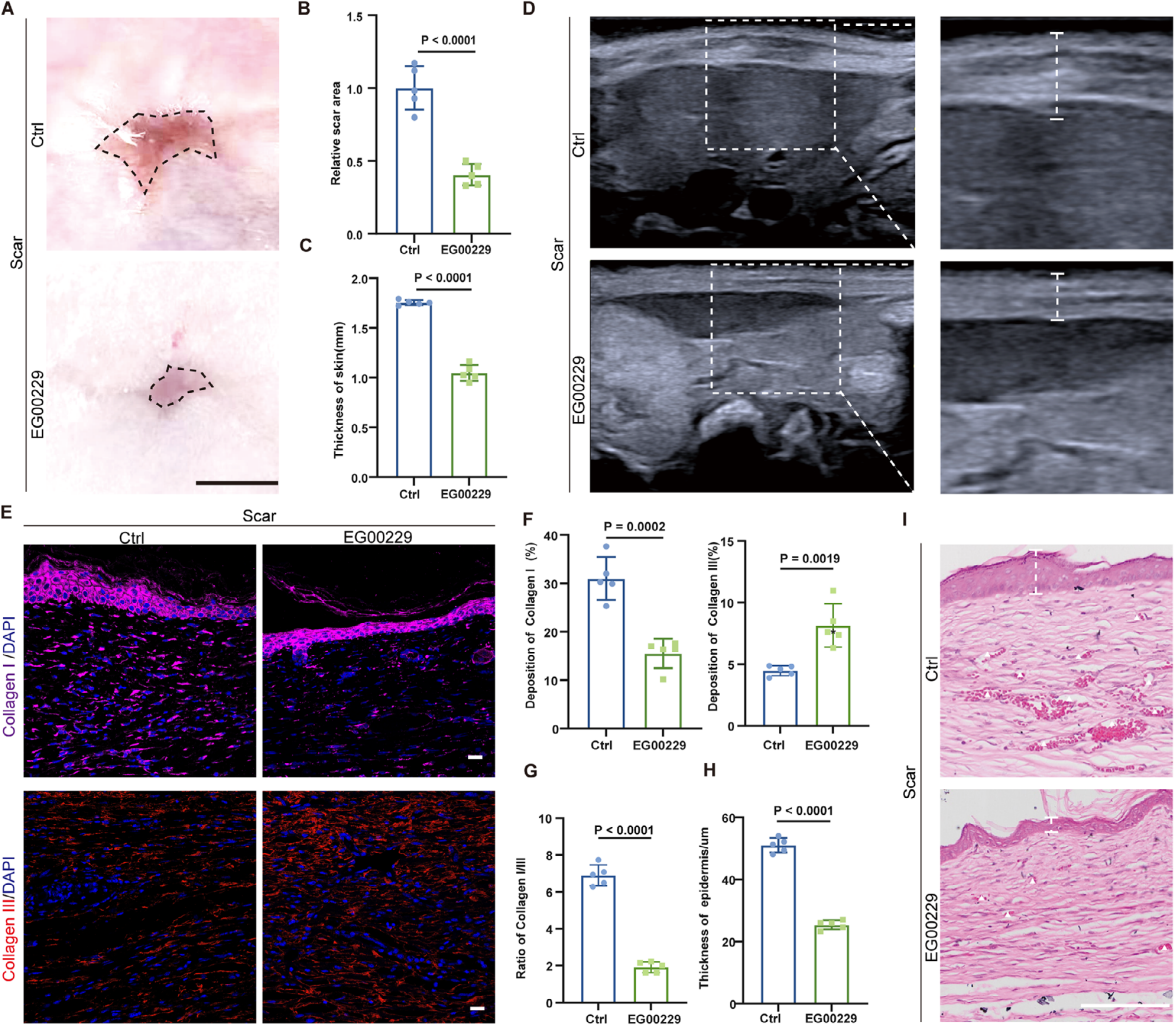
Inhibition of NRP1 prevents scar in mice scar model. A) Representative pictures of scar after treatment with EG00229 or vehicle Ctrl on the 35th day. The black dotted lines indicate the area of scar. Scale bars, 5 mm. B) Quantification of scar area treated with EG00229 or Ctrl on the 35th day (*n* = 5). C) Quantification of skin thickness in mice scar tissue treated with EG00229 or Ctrl on the 35th day (*n=5*). D) B‐ultrasonography was used to detect the thickness of scar treated with EG00229 or Ctrl on the 35th day. The white dotted lines indicate the scar area. E) Immunofluorescent staining of Collagen I (purple), Collagen III (red)in scar tissue treated with EG00229 or Ctrl on the 35th day. F) Quantification of Collagen I and Collagen III deposition in mice scar tissue treated with EG00229 or Ctrl on the 35th day (*n = 5*). H) Quantification of the ratio of Collagen I/ III in mice scar tissue treated with EG00229 or Ctrl on the 35th day (*n = 5*). H) Quantification of epidermis thickness in mice scar tissue treated with EG00229 or Ctrl on the 35th day (*n* = 5). I) Representative H&E‐stained scar treated with EG00229 or Ctrl on the 35th day. The white dotted lines indicate the area of epidermis; the triangles indicate the vessels. Scale bars, 100 µm. Statistical significance was analyzed by unpaired two‐tailed Student's *t*‐test.

Based on the above results, we further explored the role of NRP1 in scar formation. In the mouse scar model, NRP1 protein expression increased by 4.133‐fold (Figure S9A, Supporting Information), and was primarily expressed on ECs (Figure S9B, Supporting Information). To investigate NRP1‐targeting for scar prevention, we administered the NRP1 antagonist EG00229 to mice via intraperitoneal injection. Wound areas were recorded on days 0, 7, and 14. NRP1 inhibition increased the wound‐closure rate from 20 % to 63 % at day 7, and from 44 % to 84 % at day 14 (Figure S9C–E, Supporting Information). Re‐epithelialization improved from 57 % to 93 % at day 7 (Figure S9F–G, Supporting Information). NRP1 inhibition significantly improved the appearance of scars and made the surrounding skin smoother (**Figure** **6**A). After inhibition, the scar area was reduced by 59% (Figure 6B). Ultrasound showed skin thickness at the scar site dropped from 1.75 mm to 1.05 mm (Figure 6C,D). Inhibiting NRP1 decreased Collagen I from 31% to 15%, increased Collagen III from 4.4 % to 8.1 %, and decreased the ratio of Collagen I/III from 6.9 to 1.9 (Figure 6E–G). The α‐SMA deposition decreased from 26% to 11% (Figure S9H, Supporting Information). H&E staining showed that EG00229 reduced the epidermal thickness from ≈51 µm to ≈25 µm (Figure 6H,I). These results suggest that inhibiting NRP1 could prevent scar formation. NRP1 and abnormal vascularization could serve as potential targets for scar prevention.

### 2.7 Synthesis of Hydrogel Spray by NRP1‐Targeting Peptide

**Figure 7:**
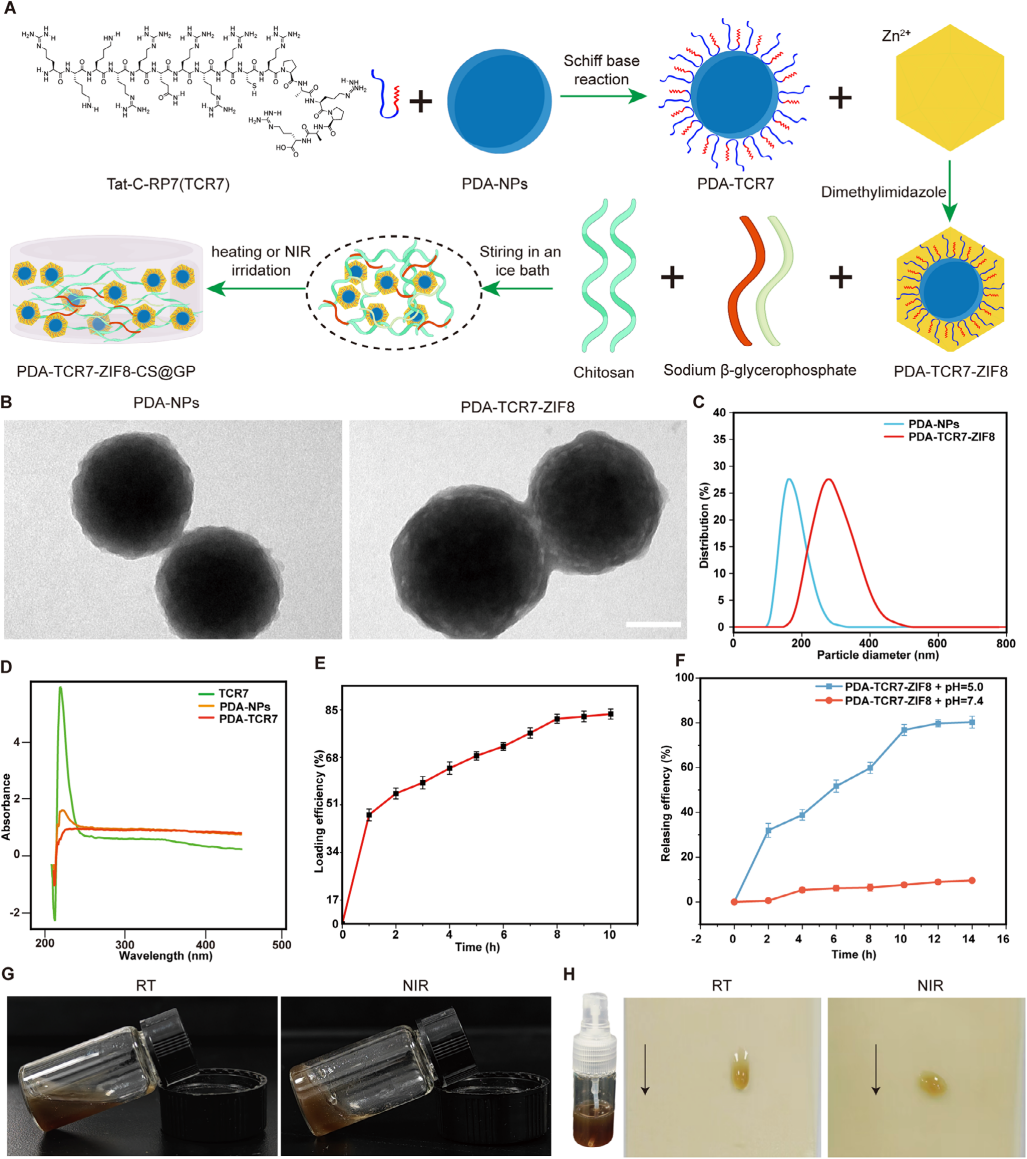
Characterization of PDA‐TCR7‐ZIF8‐CS@GP hydrogel sprayer. A) Schematic diagram about the synthesis of PDA‐TCR7‐ZIF8‐CS@GP. B) Representative TEM images of PDA‐NPs and PDA‐TCR7‐ZIF8‐NPs. Scale bar, 100 nm. C) Light scattering analysis of PDA‐NPs and PDA‐TCR7‐ZIF8‐NPs. D) UV–vis spectrum of TCR7, PDA‐NPs, PDA‐TCR7‐NPs. E) Cumulative loading efficiency of PDA‐NPs on TCR7. F) Cumulative release efficiency of PDA‐TCR7‐NPs from PDA‐TCR7‐ZIF8‐NPs in pH=5.0 and pH=7.4 solutions. G) Photograph of the PDA‐TCR7‐ZIF8‐CS@GP in the inclined tube at room temperature or under NIR irradiation. H) Photograph of the PDA‐TCR7‐ZIF8‐CS@GP hydrogel loaded in a medical spray bottle. Images showing the PDA‐TCR7‐ZIF8‐CS@GP hydrogel sprayed on a piece of vertically placed artificial skin without or with NIR irradiation.

Given NRP1's role in scar formation, we further explored targeting NRP1 to prevent scar formation. EG00229 is an organic compound. It antagonizes NRP1 by mimicking the binding of NRP1‐targeting peptide. While small peptides exhibit higher targeting specificity and better biocompatibility.^[^ ^47^ ^]^ The NRP1‐targeting peptide RP7 exhibits a strong binding affinity to NRP1. The limited tissue penetration of RP7 relies on NRP1‐mediated CendR pathway. The Tat (cell‐penetrating peptid), by contrast, enters cells and tissues via its intrinsic physicochemical properties, yielding markedly higher uptake and penetration. Linking RP7 with the Tat to form Tat‐C‐RP7 (TCR7). TCR7 could cross the blood‐brain barrier and suppress glioblastoma.^[^ ^48^ ^]^ However, small peptides suffer from poor stability and inadequate tissue selectivity. Integrating peptides with nanocarriers and biomaterials could overcome these limitations.^[^ ^49^ ^]^ Polydopamine nanoparticles (PDA‐NPs) have been widely applied in wound healing for their excellent biocompatibility and ease modification.^[^ ^49^, ^50^ ^]^ ZIF8 is a typical metal‐organic framework (MOF). ZIF8 exhibits good biocompatibility and thermal stability. Under acidic conditions, ZIF8 gradually degrades to release encapsulated drugs.^[^ ^51^ ^]^ Chitosan is a natural polymer and commonly used for thermosensitive hydrogels.^[^ ^52^ ^]^ Therefore, we conjugated TCR7 with PDA‐NPs via Schiff base reactions. And ZIF8 was used to encapsulate the complex. The entire complex was finally embedded in a chitosan hydrogel to synthesize PDA‐TCR7‐ZIF8‐CS@GP. This hydrogel demonstrates good injectability and could be used as a spray. The spray can fill and protect wounds (**Figure** **7**A).

We carried out molecular docking experiments. The results showed both TCR7 and RP7 bind to the same site on the NRP1 protein (Figure S10A,B, Supporting Information). In vivo and vitro experiments indicated that TCR7 displays good biocompatibility (Figure S11D–G, Supporting Information). PDA‐NPs were synthesized by solution oxidation. We then analyzed the morphology and size of PDA‐NPs, PDA‐TCR7‐ZIF8‐NPs, using transmission electron microscopy (TEM) and dynamic light scattering (DLS). TEM images and DLS results revealed that PDA‐TCR7‐ZIF8‐NPs exhibit a distinct core‐shell structure. Their diameters increased from ≈180 nm (PDA‐NPs) to ≈380 nm (Figure 7B,C). Compared to PDA‐NPs, PDA‐TCR7‐NPs showed distinct absorption peaks at 1053 cm^−1^ and 1498–1621 cm^−1^ (Figure S8C, Supporting Information). UV spectrophotometry results showed that only PDA‐TCR7‐NPs and TCR7 exhibit a characteristic absorption peak at 210 nm (Figure 7D). This confirmed successful conjugation of TCR7 to PDA‐NPs. The loading efficiency of TCR7 onto PDA‐NPs increased rapidly within 1 h and reached a maximum of 74% after 10 h (Figure 7E).

To evaluate the controlled release of PDA‐TCR7‐ZIF8‐NPs, we dispersed them in buffers with different pH. At pH 7.4, almost no PDA‐TCR7‐NPs were released. At pH 5.0, they released slowly and reached 80% after 14 h (Figure 7F). This is because ZIF8 is stable in physiological conditions but degrades in acidic environments. Under near‐infrared (NIR) irradiation, PDA‐TCR7‐ZIF8‐NPs exhibited similar photothermal conversion efficiency to PDA‐NPs (Figure S10D, Supporting Information). Additionally, PDA‐TCR7‐ZIF8‐NPs demonstrated good ability to scavenge reactive oxygen species (ROS) (Figure S10E, Supporting Information).

We incorporated PDA‐TCR7‐ZIF8‐NPs into a chitosan/β‐glycerophosphate hydrogel to synthesize PDA‐TCR7‐ZIF8‐CS@GP. At room temperature, the hydrogel stayed liquid. But under NIR, it turned into a gel (Figure 7G). The rheological results indicate that PDA‐TCR7‐ZIF8‐CS@GP undergoes significant crosslinking at 50 °C (Figure S11A, Supporting Information). When sprayed on artificial skin and exposed to NIR, PDA‐TCR7‐ZIF8‐CS@GP rapidly formed a gel layer. The unirradiated samples flowed off (Figure 7H). After stretching, bending, and twisting, the hydrogel demonstrates good adhesion to the artificial skin and does not easily fall off (Figure S11C, Supporting Information). In vitro tests show that the degradation rate of hydrogel reaches 75% within 18 days (Figure S11B, Supporting Information). This good degradability protects the wound from secondary injuries associated with dressing changes. SEM images showed that the hydrogel had a porous structure with lots of PDA‐TCR7‐ZIF8‐NPs inside (Figure S10F, Supporting Information). The hydrogel also converted light to heat efficiently under NIR (Figure S10G, Supporting Information). In summary, we developed the PDA‐TCR7‐ZIF8‐CS@GP hydrogel spray using the NRP1‐targeting peptide TCR7, PDA‐NPs, ZIF8, and chitosan. This system combines pH‐responsive drug release, photothermal activity, and wound‐adhesive properties. The hydrogel spray offered a targeted way to normalize vascular and prevent scars.

### 2.8 PDA‐TCR7‐ZIF8‐CS@GP Hydrogel Spray Restores Vascular Structure and Function to Prevent Scar Formation

**Figure 8:**
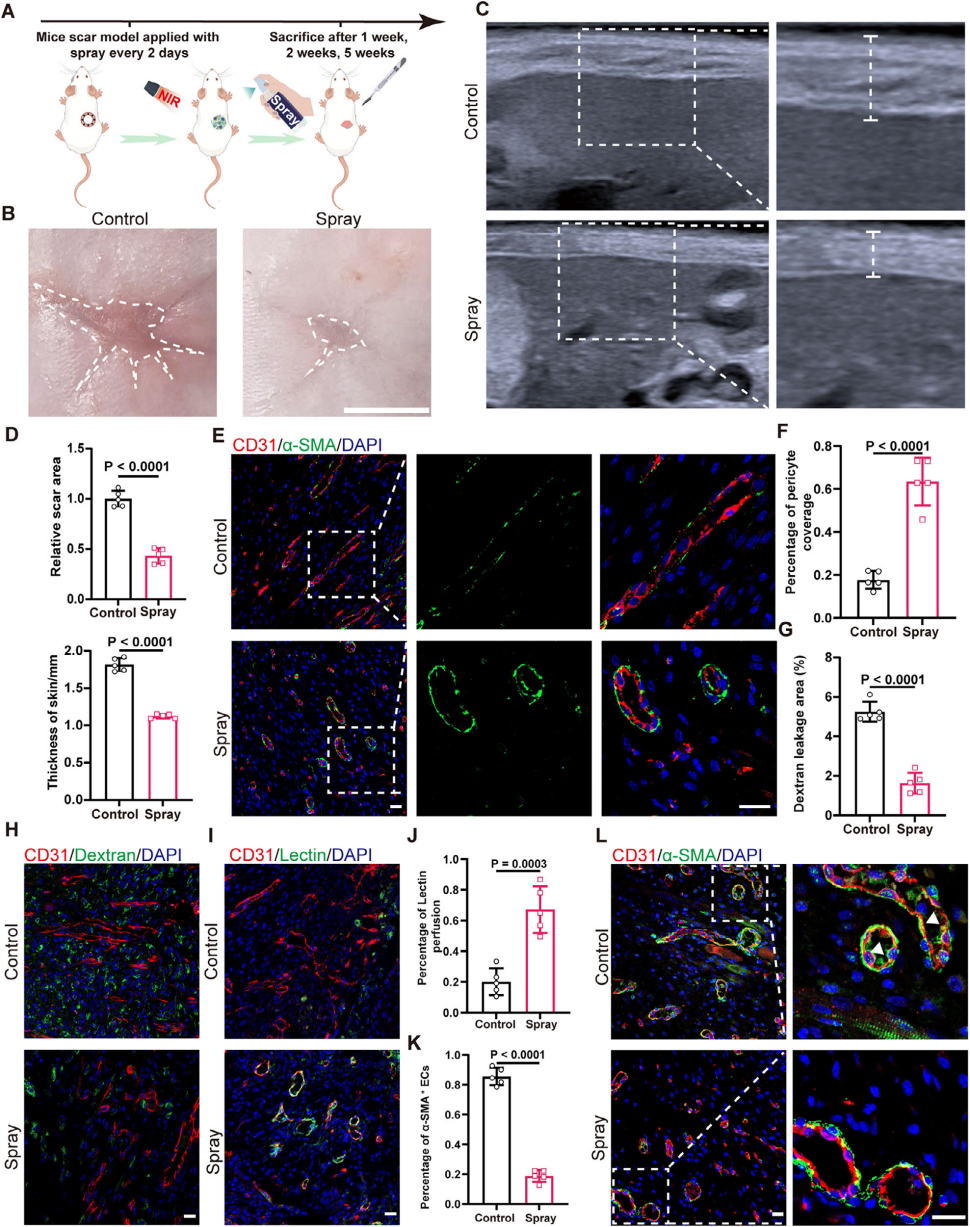
PDA‐TCR7‐ZIF8‐CS@GP hydrogel spray could normalize the function and structure of vessels, preventing scar. A) Schematic diagram of the spray applied in mice scar model. B) Representative pictures of scar after treatment with spray or control (PBS)on the 35th day. The white dotted lines indicate the area of scar. Scale bars, 5 mm. C) B‐ultrasonography was used to detect the thickness of scar treated with spray or PBS on the 35th day. The white dotted lines indicate the scar area. D) Quantification of scar area and skin thickness treated with spray or PBS on the 35th day (*n* = 5). E) Immunofluorescent staining of α‐SMA (green), CD31(red), DAPI (blue) in mice scar tissue treated with spray or not on the 7th day. The white dotted lines indicate the area of vessels. Scale bars, 20 µm. F) Quantification of pericyte coverage of vessels in mice wound tissue treated with spray or PBS on the 7th day (*n* = 5). G,H) Immunofluorescent images and quantification of dextran leakage area in mice wound tissue treated with spray or PBS on the 7th day (*n* = 5). Dextran(green), CD31(red), DAPI (blue). Scale bars, 20 µm. I,J) Immunofluorescent images and quantification of lectin perfused area in mice wound tissue treated with spray or PBS on the 7th day (*n* = 5). Lectin(green), CD31(red), DAPI (blue). Scale bars, 20 µm. K) Quantification of the percentage of α‐SMA^+^ ECs in mice wound tissue treated with spray on the 14th day (*n* = 5). L) Immunofluorescent staining of α‐SMA (green), CD31(red), DAPI (blue) in mice wound tissue treated with spray or PBS on the 14th day. The white dotted lines indicate the vessels area. The triangles indicate the ECs expressed with α‐SMA. Scale bars, 20 µm. Statistical significance was analyzed by unpaired two‐tailed Student's *t*‐test.

To evaluate the effects of PDA‐TCR7‐ZIF8‐CS@GP hydrogel spray, we applied it to a mouse scar model (**Figure 8**A). Wound areas were recorded on days 0, 7, and 14. Spray increased the wound‐closure rate from 33% to 67% on day 7, and from 65% to 84 % on day 14 (Figure S12A–C, Supporting Information). Re‐epithelialization improved from 50 % to 95% on day 7(Figure S12D,E, Supporting Information). The spray significantly reduced the scar area by 60% and improved scar appearance. Ultrasound imaging showed that the scar skin thickness decreased from ≈1.8 mm to 1.1 mm (**Figure** **8**B–D). H&E stains revealed a reduction in epidermal thickness from ≈48 µm to ≈24 µm in scar tissue (Figure S13A, Supporting Information). In scars, the spray decreased α‐SMA deposition from ≈28% to ≈13%. Additionally, the spray decreased Collagen I from ≈24% to ≈7%, and increased Collagen III from ≈4% to ≈8%. The ratio of Collagen I/III significantly decreased from 6.9% to 1.9% (Figure S13B–D, Supporting Information). These findings demonstrate that the spray effectively prevents scar formation.

**Figure 9:**
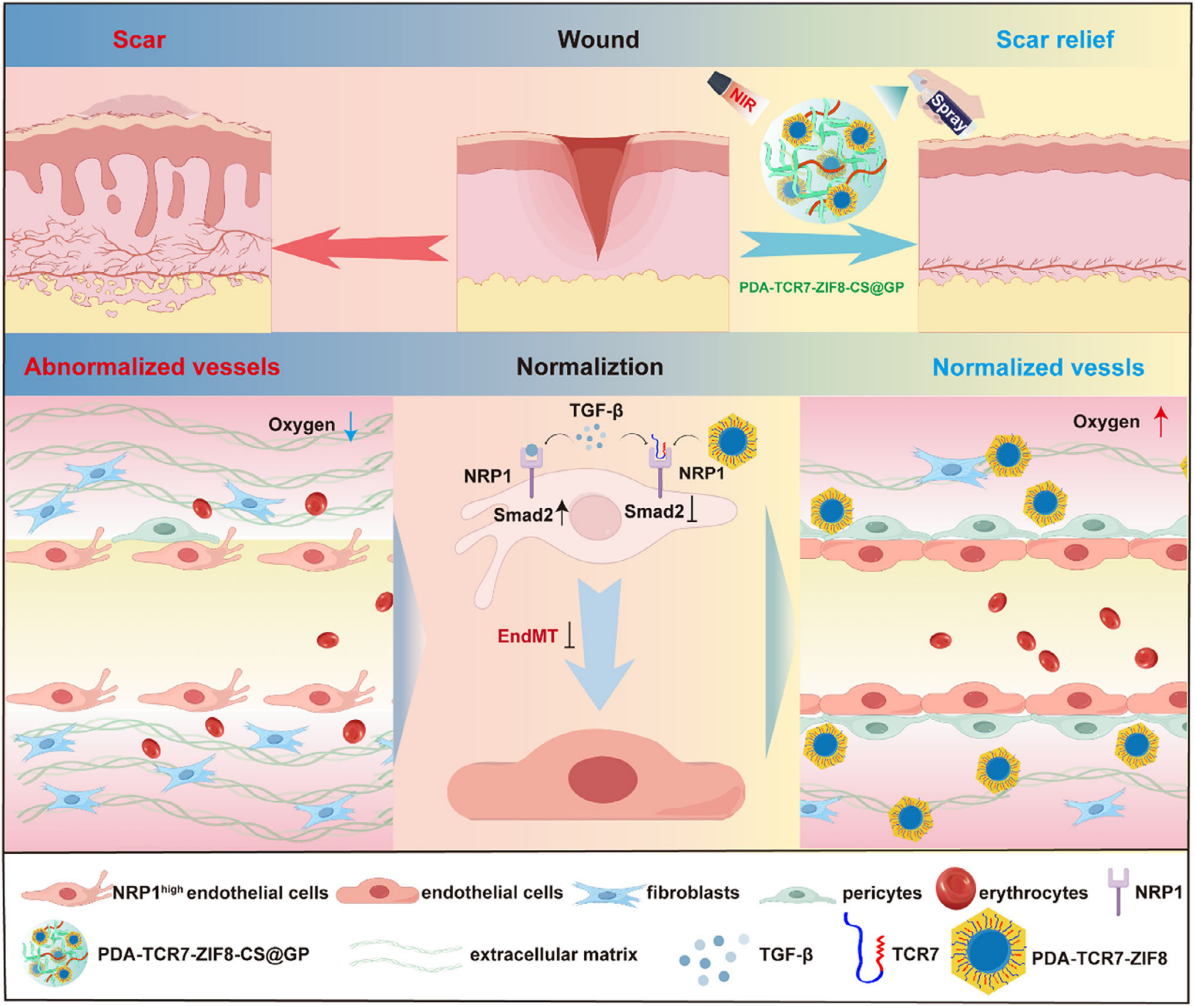
Schematic of PDA‐TCR7‐ZIF8‐CS@GP hydrogel spray to normalize the function and structure of vessels, preventing scar.

The effects of spray on the structure and function of scar vessels were further investigated. The spray reduced neovascularization by 61% and decreased vascular branching ratio by 76% (Figure S13E,F, Supporting Information). The spray increased pericyte coverage of scar vessels from 17% to 63% (Figure 8E,F). Furthermore, the permeability and perfusion functions of blood vessels were assessed by tail vein injection of FITC‐Dextran and FITC‐Lectin. The extravasation of dextran was reduced from 5.2% to 1.6% by spray (Figure 8G,H). Additionally, the proportion of lectin‐labeled vessels increased from 20% to 67% by spray, restoring vascular perfusion (Figure 8I,J). By restoring vascular structure and function, the spray significantly reduces the area of hypoxia from 12.5% to 2.8% (Figure S13G,H, Supporting Information). Moreover, the occurrence of EndMT, was inhibited by spray. Immunofluorescence staining showing a significant reduction in the proportion of ECs expressed with MYH11, α‐SMA, and FSP1(Figure 8K,L; Figure S14A–D, Supporting Information). In summary, these data indicate that targeting NRP1 can restore vascular structure and function, and prevents scar formation (**Figure** **9**).

## 3 Discussion

Vascular abnormalities are increasingly recognized as a critical factor in various pathological conditions. These abnormalities can compromise vascular barriers and lead to impaired perfusion.^[^ ^52^ ^]^ In oncology, abnormal vasculature significantly impacts not only tumor growth and metastasis but also therapeutic response.^[^ ^53^, ^54^, ^55^ ^]^Tumors often develop chaotic vascular networks. They are leaky and poorly functional. The abnormal vasculature hinders the effective delivery of chemotherapeutic agents and allows tumor cells to enter the circulation.^[^ ^56^ ^]^ Beyond cancer, vascular abnormalities are also central to fibrotic disorders. Such as systemic sclerosis and psoriasis which featured with cutaneous fibrosis.^[^ ^57^, ^58^ ^]^ These abnormalities can initiate inflammation, activate fibroblasts, and drive fibrosis.^[^ ^59^ ^]^ Excessive angiogenesis is also common in cutaneous scar formation.^[^ ^60^ ^]^ Importantly, there are similar cellular and molecular mechanisms in the angiogenic processes between scars and cancer.^[^ ^61^ ^]^ Our study delves into the vascular abnormalities in scars. Our findings reinforce the role of vascular dysfunction in scar pathogenesis and expand the theoretical framework for current scar therapies. Future research should investigate adapting vascular‐targeted therapies in cancer to manage scars.

Single‐cell RNA sequencing has expanded our understanding of cellular heterogeneity. Endothelial cells (ECs) exhibit high spatial and functional diversity.^[^ ^62^ ^]^ ECs in different tissues display distinct molecular and functional properties.^[^ ^63^, ^64^, ^65^ ^]^ In normal skin, ECs have unique metabolic and signaling features. Researchers have identified five subclusters of ECs in normal skin.^[^ ^66^, ^67^ ^]^ In keloids, a severe form of pathological scarring, ECs significantly increase and form four subclusters.^[^ ^68^ ^]^ Despite the heterogeneity of EC, most scar research has focused on fibroblast heterogeneity.^[^ ^69^, ^70^, ^71^ ^]^ So, there is a gap in the understanding of ECs in scar formation. To address this, we constructed a comprehensive EC atlas for healthy skin and scar. This atlas uncovered the transcriptional heterogeneity and molecular features in scar ECs. Our study showed that scar ECs are in a metabolically stressed state. The upregulated genes in scar ECs were concentrated in ATP synthesis, decomposition, and oxidative phosphorylation. Metabolic issued significantly affect EC functions, such as angiogenesis,^[^ ^72^ ^]^ barrier integrity,^[^ ^73^ ^]^ inflammation,^[^ ^74^ ^]^ and cellular crosstalk.^[^ ^75^ ^]^ We also found three EC subclusters in scars. The subcluster 1 may drive abnormal vascularization in scars. These results enhance our comprehension of scar pathogenesis. This could offer new directions for scar research and the development of targeted therapies.

NRP1 is known to promote fibrosis in multiple organs, including the liver and lung.^[^ ^76^, ^77^ ^]^ And NRP1 is also implicated in tumor angiogenesis.^[^ ^78^ ^]^ But its role in skin scar formation has not been well understood. Our study shows that *NRP1* is significantly upregulated in scar‐associated ECs. The *NRP1* ^high^ ECs were specifically activated in scar tissue. Pseudotime analysis revealed that genes associated with tumor angiogenesis and EMT(such as *MDK*, *COL1AI*, *ACTG1*, *IGFBP7*, *VIM*, and *ZEB1*)^[^ ^33^, ^34^, ^35^, ^36^, ^37^ ^]^ are significantly upregulated in *NRP1* ^high^ ECs. This aligns with previous reports that human keloid ECs express mesenchymal markers.^[^ ^79^ ^]^ The promotion of EndMT exacerbate skin scar formation, while its inhibition mitigates scar.^[^ ^80^, ^81^ ^]^ We identified *NRP1* ^high^ ECs in scar tissue. These ECs were linked to abnormal vascular changes and exhibited mesenchymal characteristics. Our findings suggested NRP1 could be a preventive target for skin scars. These results enhance our understanding of scar formation mechanisms and offer new avenues for targeted prevention development.

NRP1 is a single‐pass transmembrane receptor without enzymatic activity.^[^ ^82^ ^]^ Its extensive extracellular tail contains multiple domains. This enables NRP1 to interact with various ligands and participate in diverse signaling pathways.^[^ ^83^ ^]^ Notably, NRP1 plays a crucial role in enhancing TGF‐β signaling. For example, NRP1‐positive distal tubular epithelial cells are activated by myofibroblast‐derived TGF‐β. This enhances SMAD3 expression and exacerbates renal fibrosis.^[^ ^84^ ^]^ In our study, we found that the TGF‐β signaling pathway is significantly activated in *NRP1* ^high^ ECs. Additionally, TGF‐β upregulated NRP1 in HUVECs. By analyzing single cell sequencing data and in vitro experiments, we demonstrated that TGF‐β upregulates NRP1 in ECs and promotes EndMT via SMAD2. These findings indicate that TGF‐β may be crucial for EndMT and the vascular abnormalities. Our research further elucidates the molecular mechanisms underlying NRP1's role in fibrosis. And this could be provided as therapeutic targets for scars.

In a mouse model of pulmonary fibrosis, treatment with the NRP1‐targeting small‐molecule inhibitor EG00229 significantly reduced collagen deposition, slowed disease progression, and lowered mortality by inhibiting ILC2 function.^[^ ^77^ ^]^ Abnormal skin blood vessels are a hallmark of psoriasis; targeting IGFBP7^hi^ ECs can restore vascular structure, reduce inflammatory infiltration, and alleviate psoriasis.^[^ ^85^ ^]^ Given that abnormal blood vessels may underlie keloid and scar pathogenesis,^[^ ^86^ ^]^ we combined a mouse scar model with the NRP1 inhibitor EG00229. Our results indicate that vascular structure and function abnormalities are essential for scar formation. Targeting NRP1 and vascular abnormalities is a viable strategy to restore blood vessel structure and function, thereby preventing scar formation. Collectively, these findings suggest that NRP1 is a promising therapeutic target for preventing fibrotic conditions.

Our work further explored the translational potential of targeting NRP1 for scar. Peptides are ease of synthesis with high specificity and biocompatibility.^[^ ^47^ ^]^ But they degrade quickly and don't penetrate tissue well.^[^ ^49^ ^]^ To overcome these limitations, we developed a hydrogel spray delivery system. We synthesized a novel NRP1‐targeting nanomaterial by conjugating small peptides to polydopamine nanoparticles and coating them with a ZIF8 shell. This design enhances peptide stability in the acidic wound microenvironment and enables controlled release.^[^ ^87^ ^]^ We encapsulated this nanomaterial into a chitosan hydrogel. When applied to wounds, it effectively restored vascular structure and function in scar tissue, inhibiting scar formation. Our findings suggested that NRP1 and abnormal vessels could be promising targets for scar prevention.

There are some limitations in our study. We investigated the vascular and EC alterations in scar tissue. And their role in scar formation had been validated. But the exact mechanisms about how these changes contributing to scar formation were not fully understood. In human keloids, a subcluster of ECs with mesenchymal traits is particularly activated. This EC subcluster interacts significantly with keloid fibroblasts.^[^ ^79^ ^]^ Research showed that vascular irregularities in psoriasis can drive T cell infiltration and inflammation. NRP1 supports Treg cell's function. It could enhance Treg cell recruitment and stability in chronic inflammation.^[^ ^88^ ^]^ Our single cell sequencing data showed T cells increased in scars (Figure 1E). In *NRP1* ^high^ ECs, antigen processing and presentation pathways were upregulated (Figure S5A, Supporting Information). These findings indicate that the abnormal blood vessels and ECs may interact with immune cells to enhance scar formation. Still, more research is needed to fully understand these mechanisms.

Although our findings in rodent models demonstrate the therapeutic potential of targeting NRP1, translation to clinical application requires addressing potential species‐specific differences. Our future validation strategy will progress through large animal models (porcine and non‐human primate) with greater physiological similarity to humans, followed by human‐derived platforms including skin organoids and ex vivo pathological scar cultures. This stepwise approach will provide comprehensive evidence for the clinical translatability of NRP1‐targeted anti‐fibrotic interventions.

In this study, we found that *NRP1* ^high^ ECs are key drivers of vascular abnormalities in scar tissue. By targeting and inhibiting NRP1, we could promote vascular normalization and prevent scar. We also developed a novel hydrogel spray targeting NRP1. This spray was proved effective in scar prevention. These results suggest that modulating vascular ECs through NRP1 is a promising approach for scar prevention. This not only provides valuable insights into the mechanisms of scar formation but also offers potential for future clinical applications.

## 4 Experimental Section

### Human Tissue Samples

The study was approved by the Ethics Committee of the First Affiliated Hospital of Army Medical University under approval numbers KY2022115. Skin and scar tissues were acquired after obtaining written informed consent. The clinical samples utilized in this study were obtained from 4 healthy individuals and 4 patients diagnosed with hypertrophic scarring. The demographic information, including sex, age, height, weight, ethnicity, location of sample collection, medical history and pathology of the subjects, was presented in Table S2 (Supporting Information). Prior to core resection, none of the patients had received chemotherapy, radiotherapy or intralesional hormone injections for scar site. To avoid any potential confounding effects due to site‐specific characteristics, tissue biopsies were collected from the central region of the scar rather than from its periphery. The skin tissue was washed twice in PBS and was partly fixed with 4% paraformaldehyde or electron microscopy fixative solution for immunofluorescent analyses and scanning electron microscopy observation.

### Analysis of Human Single Cell Sequencing Data from Public Databases

This study integrated 6 single‐cell transcriptome data from the Gene Expression Omnibus (https://www.ncbi.nlm.nih.gov/geo/), including 3 hypertrophic scar samples and 3 normal skin samples. These 6 samples were obtained from 1 dataset (GSE156326). Detailed information of these samples was provided in Table S1 (Supporting Information). Raw count data from these 6 samples were extract and integrate them into the Seurat object. Use DoubletFinder (v.2.0.3) to detect and filter out Doublets, further filtering out a small number of characteristic cells (nFeature_RNA<500) and a high percentage of mitochondrial gene cells (over 25%). Using Harmony software package (v 0.1.1), calibrate and evaluate the batch effects between various datasets and samples, and proceed with the next step of dimensionality reduction clustering analysis after meeting the requirements. Manually annotate the obtained clusters using unique markers.

### Assessment of Vascular Perfusion and Leakage

For vessel perfusion quantification, FITC‐Lectin (1 mg mL^−1^,100 µL, Sigma–Aldrich, #L0401,) was intravenously injected into the mice through the tail vein and protected from light. After 30 mins, the mice were euthanized, and scar tissues were harvested. Frozen sections were then subjected to IF with anti‐CD31 (1:100, Abcam, #ab281583). Finally, the slices were photographed using a confocal fluorescence microscope (LSM800, Zeiss, Germany). The views to respectively count CD31+ vessels and Lectin+ vessels (Lectin+CD31+ vessels) were randomly selected, and their ratios were calculated. To evaluate vessel leakage, FITC‐ Dextran (70 kDa,25 mg mL^−1^, Sigma Aldrich, #46945) was intravenously injected into mice. After removing scar tissues from euthanized mice, they were cut into cryosections. The following procedures were the same as described above. Dextran+ area (%) = Dextran+ area/ total sectional area× 100%.

### Animals

All mice were maintained on a BALB/c background and housed under specific‐pathogen free (SPF) conditions. All animal surgeries were performed with inhalation anesthesia with isoflurane at a concentration of 1 to 2% in oxygen at 3 liters/min. All mouse experiments were performed in accordance with the guidelines of the Institutional Animal Care and Use Committees of the Army Medical University under approval numbers AMUWEC20211795.

### Statistics Analysis

GraphPad Prism 8.0 (San Diego, CA, USA) was used to analyze the data, which were presented as the mean ± SEM. The statistical significance of differences between two groups was assessed using a two‐tailed Student's *t*‐test. All statistical analyses were performed using the Prism software package (Prism 8.3.0, GraphPad Software). Statistical significance was defined as ^*^ *P* < 0.05, ^**^ *P* < 0.01, ^***^ *P* < 0.001.

## Conflict of Interest

The authors declare that they have no competing interests.

## Author's Contributions

Y.W., X.Z., M.L., M.H. and P.C. contributed equally to this work. Conceptualization was carried out by YW, XZ, ML, YL, and WZ. Methodology was developed by YW, XZ, ML, MH, XL, FX, WZ, DL, and LL, while investigation efforts were undertaken by MH, XL, FX, WZ, DL, and LL. Visualization was performed by YW, XZ, and ML. Funding acquisition was supported by ML, YL, and WZ. Project administration was managed by YW, XZ, YL, and WZ, and supervision was provided by YW, XZ, ML, YL, and WZ. The original draft was written by YW, XZ, and ML, with review and editing conducted by YW, XZ, ML, YL, and WZ.

## Supporting information



Supporting Information

## Data Availability

The data that support the findings of this study are available in the supplementary material of this article.

## References

[1] T. Mishra , S. Wairkar , Tissue Cell 2025, 94, 102800. 39999656 10.1016/j.tice.2025.102800

[2] Y. Cao , R. Langer , N. Ferrara , Nat. Rev. Drug Discov. 2023, 22, 476. 37041221 10.1038/s41573-023-00671-z

[3] R. Leszczynski , S. C. Da , A. Pinto , U. Kuczynski , S. E. Da , Cochrane Database Syst. Rev. 2022, 9, CD011642. 36161591 10.1002/14651858.CD011642.pub2 PMC9511989

[4] M. Rodrigues , N. Kosaric , C. A. Bonham , G. C. Gurtner , Physiol. Rev. 2018, 99, 665. 10.1152/physrev.00067.2017 PMC6442927 30475656

[5] M. Zhao , L. Wang , M. Wang , S. Zhou , Y. Lu , H. Cui , A. C. Racanelli , L. Zhang , T. Ye , B. Ding , B. Zhang , J. Yang , Y. Yao , Signal Transduct. Target Ther. 2022, 7, 206. 35773269 10.1038/s41392-022-01070-3 PMC9247101

[6] M. Xu , H. H. Xu , Y. Lin , X. Sun , L. J. Wang , Z. P. Fang , X. H. Su , X. J. Liang , Y. Hu , Z. M. Liu , Y. Cheng , Y. Wei , J. Li , L. Li , H. J. Liu , Z. Cheng , N. Tang , C. Peng , T. Li , T. Liu , L. Qiao , D. Wu , Y. Q. Ding , W. J. Zhou , Cell 2019, 178, 1478. 31474362 10.1016/j.cell.2019.07.021

[7] A. Hellmut G , K. Gou Young , Science 2017, 357, 2379.

[8] M. G. Jesus , I. Tomer , H. Sean , B. Chaitanya , L. Yang , K. Viktoria , D. Neil , P. Claire , Y. Masataka , W. Matthew , L. Tyler , L. Ge , X. Jenny Zhaoying , H. Yen‐Michael Sheng , R. David , S. Ryan , B. Graeme , R. Anna , R. Shahin , Nat. Cardiovasc. Res. 2023, 1, 882.

[9] N. Dufton , C. Peghaire , L. O. Almagro , C. Raimondi , V. Kalna , A. Chauhan , G. J. Webb , Y. Yang , G. M. Birdsey , P. Lalor , J. C. Mason , D. H. Adams , J. C Mason , D. H Adams , A. M. Randi , Nat. Commun. 2020, 11, 1301. 32139699 10.1038/s41467-020-15151-w PMC7058024

[10] K. Joanna , D. R. Laura P M H , G. Jermaine , R. Katerina , D. Sébastien J , M. Elda , C. Nadine V , T. Federico , T. Laure‐Anne , V. Koen , G. Melissa , K. Shawez , G. Vincent , S. Liliana , C. Rongyuan , T. Lucas , B. Mila , D. Z. Pauline , D. Charlotte , K. Tobias K , F. Kim D , P. Magdalena , Y. Xiangke , V. Stefan , D. Yuxiang , F. Robert A , S. Luc , D. Mieke , E. Guy , T. Bernard , et al., Cell 2020, 180, 764.32059779

[11] S. Jonas C , A. Taylor S , C. Carlos Jr , R. Micha Sam Brickman , Y. Yifan , O. Norihito , P. Sergio , C. Maurizio , R. Kadi‐Ann , M. Edward P , S. Maor , D. Giuseppe , A. Farida , N. Nir , H. Arun C , G. Austin J , B. Linh T , L. Robert , P. Richard W , M. Kerstin B , N. Martijn C , T. Sarah A , B. Nicholas E , K. Jonathan A , N. Laura E , P. Dana , Y. Xiting , H. Robert J , R. Ivan O , K. Naftali , Circulation 2021, 144, 286.34030460

[12] J. M. Gomez‐Salinero , D. Redmond , S. Rafii , Nat. Rev. Mol. Cell Biol. 2025, 26, 476. 39875728 10.1038/s41580-024-00825-w

[13] H. E. Talbott , S. Mascharak , M. Griffin , D. C. Wan , M. T. Longaker , Cell Stem Cell 2022, 29, 1161. 35931028 10.1016/j.stem.2022.07.006 PMC9357250

[14] S. Mascharak , H. E. Desjardins‐Park , M. F. Davitt , M. Griffin , M. R. Borrelli , A. L. Moore , K. Chen , B. Duoto , M. Chinta , D. S. Foster , A. H. Shen , M. Januszyk , S. H. Kwon , G. Wernig , D. C. Wan , H. P. Lorenz , G. C. Gurtner , M. T. Longaker , Science 2021, 372, 237. 33888614 10.1126/science.aba2374 PMC9008875

[15] D. C. West , C. G. Rees , L. Duchesne , S. J. Patey , C. J. Terry , J. E. Turnbull , M. Delehedde , C. W. Heegaard , F. Allain , C. Vanpouille , D. Ron , D. G. Fernig , J. Biol. Chem. 2005, 280, 13457. 15695515 10.1074/jbc.M410924200

[16] M. Berge , D. Allanic , P. Bonnin , C. de Montrion , J. Richard , M. Suc , J. F. Boivin , J. O. Contreres , B. P. Lockhart , M. Pocard , B. I. Levy , G. C. Tucker , G. Tobelem , T. Merkulova‐Rainon , J. Hepatol. 2011, 55, 866. 21338642 10.1016/j.jhep.2011.01.033

[17] S. Koch , L. A. van Meeteren , E. Morin , C. Testini , S. Westrom , H. Bjorkelund , S. Le Jan , J. Adler , P. Berger , L. Claesson‐Welsh , Dev. Cell 2014, 28, 633. 24656741 10.1016/j.devcel.2014.02.010

[18] R. Fu , Du W. , Z. Ding , Y. Wang , Y. Li , J. Zhu , Y. Zeng , Y. Zheng , Z. Liu , J. A. Huang , Cell Death Dis. 2021, 12, 394. 33850110 10.1038/s41419-021-03682-z PMC8044151

[19] T. Kitsukawa , A. Shimono , A. Kawakami , H. Kondoh , H. Fujisawa , Development 1995, 121, 4309. 8575331 10.1242/dev.121.12.4309

[20] H. Q. Miao , P. Lee , H. Lin , S. Soker , M. Klagsbrun , FASEB J. 2000, 14, 2532. 11099472 10.1096/fj.00-0250com

[21] C. Raimondi , A. Fantin , A. Lampropoulou , L. Denti , A. Chikh , C. Ruhrberg , J. Exp. Med. 2014, 211, 1167. 24863063 10.1084/jem.20132330 PMC4042645

[22] C. Raimondi , C. Ruhrberg , Semin. Cell Dev. Biol. 2013, 24, 172. 23319134 10.1016/j.semcdb.2013.01.001

[23] C. A. Chuckran , C. Liu , T. C. Bruno , C. J. Workman , D. A. Vignali , J. Immunother. Cancer 2020, 8, 000967. 10.1136/jitc-2020-000967 PMC7368550 32675311

[24] M. Leclerc , E. Voilin , G. Gros , S. Corgnac , V. de Montpreville , P. Validire , G. Bismuth , F. Mami‐Chouaib , Nat. Commun. 2019, 10, 3345. 31350404 10.1038/s41467-019-11280-z PMC6659631

[25] Y. Chen , Q. Pu , Y. Ma , H. Zhang , T. Ye , C. Zhao , X. Huang , Y. Ren , L. Qiao , H. M. Liu , C. T. Esmon , B. S. Ding , Z. Cao, Cell Metab. 2021, 33, 395. 10.1016/j.cmet.2020.11.019 33357457

[26] S. Perrotta , L. Carnevale , M. Perrotta , F. Pallante , T. P. Mikolajczyk , V. Fardella , A. Migliaccio , S. Fardella , S. Nejat , B. Kapelak , A. Zonfrilli , J. Pacella , F. Mastroiacovo , R. Carnevale , C. Bain , S. L. Puhl , G. D'Agostino , S. Epelman , T. J. Guzik , G. Lembo , D. Carnevale , Immunity 2025, 58, 648. 40023160 10.1016/j.immuni.2025.02.013

[27] P. N. Matkar , K. K. Singh , D. Rudenko , Y. J. Kim , M. A. Kuliszewski , G. J. Prud'Homme , D. W. Hedley , H. Leong‐Poi , Oncotarget 2016, 7, 69489. 27542226 10.18632/oncotarget.11060 PMC5342493

[28] B. Cruys , B. W. Wong , A. Kuchnio , D. Verdegem , A. R. Cantelmo , L. C. Conradi , S. Vandekeere , A. Bouche , I. Cornelissen , S. Vinckier , R. M. Merks , E. Dejana , H. Gerhardt , M. Dewerchin , K. Bentley , P. Carmeliet , Nat. Commun. 2016, 7, 12240. 27436424 10.1038/ncomms12240 PMC4961802

[29] C. Viallard , B. Larrivee , Angiogenesis 2017, 20, 409. 28660302 10.1007/s10456-017-9562-9

[30] M. Schafer , S. Werner , Nat. Rev. Mol. Cell Biol. 2008, 9, 628. 18628784 10.1038/nrm2455

[31] Z. Hou , Y. Liu , M. Zhang , L. Zhao , X. Jin , L. Liu , Z. Su , H. Cai , Y. Qin , Commun. Biol. 2021, 4, 1149. 34599277 10.1038/s42003-021-02676-z PMC8486858

[32] T. Mu , L. Xu , Y. Zhong , X. Liu , Z. Zhao , C. Huang , X. Lan , C. Lufei , Y. Zhou , Y. Su , L. Xu , M. Jiang , H. Zhou , X. Lin , L. Wu , S. Peng , S. Liu , S. Brix , M. Dean , N. R. Dunn , K. S. Zaret , X. Y. Fu , Y. Hou , Commun. Biol. 2020, 3, 642. 33144666 10.1038/s42003-020-01364-8 PMC7642341

[33] J. Q. Luo , T. W. Yang , J. Wu , H. H. Lai , L. B. Zou , W. B. Chen , X. M. Zhou , D. J. Lv , S. R. Cen , Z. N. Long , Y. Y. Mao , P. X. Zheng , X. H. Su , Z. Y. Xian , F. P. Shu , X. M. Mao , Cell Death Dis. 2023, 14, 502. 37542027 10.1038/s41419-023-06007-4 PMC10403531

[34] C. Richter , D. Mayhew , J. P. Rennhack , J. So , E. H. Stover , J. H. Hwang , D. Szczesna‐Cordary , Int. J. Mol. Sci. 2020, 21, 8690. 33217970 10.3390/ijms21228690 PMC7698702

[35] Y. Sun , W. Chen , R. J. Torphy , S. Yao , G. Zhu , R. Lin , R. Lugano , E. N. Miller , Y. Fujiwara , L. Bian , L. Zheng , S. Anand , F. Gao , W. Zhang , S. E. Ferrara , A. E. Goodspeed , A. Dimberg , X. J. Wang , B. H. Edil , C. C. Barnett , R. D. Schulick , L. Chen , Y. Zhu , Sci. Transl. Med. 2021, 13, abc8922. 10.1126/scitranslmed.abc8922 PMC8749958 34321321

[36] W. Wang , T. Li , Y. Cheng , F. Li , S. Qi , M. Mao , J. Wu , Q. Liu , X. Zhang , X. Li , L. Zhang , H. Qi , L. Yang , K. Yang , Z. He , S. Ding , Z. Qin , Y. Yang , X. Yang , C. Luo , Y. Guo , C. Wang , X. Liu , L. Zhou , Y. Liu , W. Kong , J. Miao , S. Ye , M. Luo , L. An , et al., Cancer Cell 2024, 42, 815. 38640932 10.1016/j.ccell.2024.03.013

[37] Z. Huang , Y. Li , Y. Qian , E. Zhai , Z. Zhao , T. Zhang , Y. Liu , L. Ye , R. Wei , R. Zhao , Z. Li , Z. Liang , S. Cai , J. Chen , Cell Death Dis. 2024, 15, 756. 39424639 10.1038/s41419-024-07153-z PMC11489581

[38] S. Piera‐Velazquez , S. A. Jimenez , Physiol. Rev. 2019, 99, 1281. 30864875 10.1152/physrev.00021.2018 PMC6734087

[39] S. Lovisa , E. Fletcher‐Sananikone , H. Sugimoto , J. Hensel , S. Lahiri , A. Hertig , G. Taduri , E. Lawson , R. Dewar , I. Revuelta , N. Kato , C. J. Wu , R. J. Bassett , N. Putluri , M. Zeisberg , E. M. Zeisberg , V. S. Lebleu , R. Kalluri , Sci. Signal 2020, 13, aaz2597. 10.1126/scisignal.aaz2597 PMC7790440 32518142

[40] D. M. Gonzalez , D. Medici , Sci. Signal 2014, 7, re8. 25249658 10.1126/scisignal.2005189 PMC4372086

[41] T. Watabe , K. Takahashi , K. Pietras , Y. Yoshimatsu , Semin. Cancer Biol. 2023, 92, 130. 37068553 10.1016/j.semcancer.2023.04.007

[42] L. Hong , Du X. , W. Li , Y. Mao , L. Sun , X. Li , Eur. J. Cell Biol. 2018, 97, 493. 30082099 10.1016/j.ejcb.2018.07.005

[43] Y. Wang , J. Wang , G. Liu , X. Yi , J. Wu , H. Cao , L. Zhang , P. Zhou , Y. Fan , Y. Yu , Q. Liu , Z. Yao , H. Wang , J. Zhou , Cell Mol. Immunol. 2025, 22, 161. 39741194 10.1038/s41423-024-01246-7 PMC11782674

[44] Z. Xu , C. Guo , Q. Ye , Y. Shi , Y. Sun , J. Zhang , J. Huang , Y. Huang , C. Zeng , X. Zhang , Y. Ke , H. Cheng , Nat. Commun. 2021, 12, 6310. 34728626 10.1038/s41467-021-26697-8 PMC8564544

[45] J. S. Park , I. K. Kim , S. Han , I. Park , C. Kim , J. Bae , S. J. Oh , S. Lee , J. H. Kim , D. C. Woo , Y. He , H. G. Augustin , I. Kim , D. Lee , G. Y. Koh , Cancer Cell 2017, 31, 157. 28073001 10.1016/j.ccell.2016.12.009

[46] C. Qian , Y. Zhou , T. Zhang , G. Dong , M. Song , Y. Tang , Z. Wei , S. Yu , Q. Shen , W. Chen , J. P. Choi , J. Yan , C. Zhong , L. Wan , J. Li , A. Wang , Y. Lu , Y. Zhao , Acta Pharm. Sin. B 2024, 14, 2077. 38799619 10.1016/j.apsb.2024.02.003 PMC11121179

[47] S. M. Corsello , J. A. Bittker , Z. Liu , J. Gould , P. Mccarren , J. E. Hirschman , S. E. Johnston , A. Vrcic , B. Wong , M. Khan , J. Asiedu , R. Narayan , C. C. Mader , A. Subramanian , T. R. Golub , Nat. Med. 2017, 23, 405. 28388612 10.1038/nm.4306 PMC5568558

[48] L. Zhao , H. Chen , L. Lu , C. Zhao , C. V. Malichewe , L. Wang , X. Guo , X. Zhang , Life Sci. 2021, 270, 119113. 33508290 10.1016/j.lfs.2021.119113

[49] J. You , Y. Guo , Z. Dong , Pharmaceutics 2024, 16, 1192. 39339228 10.3390/pharmaceutics16091192 PMC11435007

[50] P. Ge , S. Chang , T. Wang , Q. Zhao , G. Wang , B. He , Nanoscale 2023, 15, 644. 36515078 10.1039/d2nr04908b

[51] Z. Han , L. Deng , S. Chen , H. Wang , Y. Huang , Burns Trauma 2023, 11, tkac048. 36751362 10.1093/burnst/tkac048 PMC9897938

[52] C. Liu , P. Yang , J. Li , S. Cao , J. Shi , Carbohydr. Polym. 2022, 295, 119853. 35988979 10.1016/j.carbpol.2022.119853

[53] S. Guelfi , K. Hodivala‐Dilke , G. Bergers , Nat. Rev. Cancer 2024, 24, 655. 39210063 10.1038/s41568-024-00736-0

[54] S. Goel , D. G. Duda , L. Xu , L. L. Munn , Y. Boucher , D. Fukumura , R. K. Jain , Physiol. Rev. 2011, 91, 1071. 21742796 10.1152/physrev.00038.2010 PMC3258432

[55] S. Goel , D. G. Duda , L. Xu , L. L. Munn , Y. Boucher , D. Fukumura , R. K. Jain , Physiol. Rev. 2011, 91, 1071. 21742796 10.1152/physrev.00038.2010 PMC3258432

[56] P. Carmeliet , R. K. Jain , Nat. Rev. Drug Discov. 2011, 10, 417. 21629292 10.1038/nrd3455

[57] Q. Li , B. Pang , E. Dang , G. Wang , J. Invest. Dermatol. 2024, 144, 1935. 38493385 10.1016/j.jid.2024.02.013

[58] H. Yin , O. Distler , L. Shen , X. Xu , Y. Yuan , R. Li , B. Liu , Q. Li , Q. Huang , F. Xie , Z. Zhang , R. Liang , X. Dai , X. Chen , B. Li , Q. Yan , L. Lu , Arthritis Rheumatol. 2024, 76, 78. 37488975 10.1002/art.42662

[59] F. Rieder , L. E. Nagy , T. M. Maher , J. Distler , R. Kramann , B. Hinz , M. Prunotto , Nat. Rev. Drug Discov. 2025, 24, 543. 40102636 10.1038/s41573-025-01158-9 PMC13264708

[60] X. Liu , W. Chen , Q. Zeng , B. Ma , Z. Li , T. Meng , J. Chen , N. Yu , Z. Zhou , X. Long , J. Invest. Dermatol. 2022, 142, 124. 34242659 10.1016/j.jid.2021.06.010

[61] M. Schafer , S. Werner , Nat. Rev. Mol. Cell Biol. 2008, 9, 628. 18628784 10.1038/nrm2455

[62] J. M. Gomez‐Salinero , D. Redmond , S. Rafii , Nat. Rev. Mol. Cell Biol. 2025, 26, 476. 39875728 10.1038/s41580-024-00825-w

[63] J. Kalucka , L. de Rooij , J. Goveia , K. Rohlenova , S. J. Dumas , E. Meta , N. V. Conchinha , F. Taverna , L. A. Teuwen , K. Veys , M. Garcia‐Caballero , S. Khan , V. Geldhof , L. Sokol , R. Chen , L. Treps , M. Borri , P. de Zeeuw , C. Dubois , T. K. Karakach , K. D. Falkenberg , M. Parys , X. Yin , S. Vinckier , Du Y. , R. A. Fenton , L. Schoonjans , M. Dewerchin , G. Eelen , B. Thienpont , et al., Cell 2020, 180, 764. 32059779 10.1016/j.cell.2020.01.015

[64] M. Vanlandewijck , L. He , M. A. Mae , J. Andrae , K. Ando , G. F. Del , K. Nahar , T. Lebouvier , B. Lavina , L. Gouveia , Y. Sun , E. Raschperger , M. Rasanen , Y. Zarb , N. Mochizuki , A. Keller , U. Lendahl , C. Betsholtz , Nature 2018, 554, 475. 29443965 10.1038/nature25739

[65] H. G. Augustin , G. Y. Koh , Science 2017, 357, aal2379. 10.1126/science.aal2379 28775214

[66] Q. Li , S. Shao , Z. Zhu , J. Chen , J. Hao , Y. Bai , B. Li , E. Dang , G. Wang , J. Clin. Invest. 2023, 133, 160451. 10.1172/JCI160451 PMC10145935 36917196

[67] Q. Li , Z. Zhu , L. Wang , Y. Lin , H. Fang , J. Lei , T. Cao , G. Wang , E. Dang , Theranostics 2021, 11, 6461. 33995668 10.7150/thno.54917 PMC8120211

[68] X. Liu , W. Chen , Q. Zeng , B. Ma , Z. Li , T. Meng , J. Chen , N. Yu , Z. Zhou , X. Long , J. Invest. Dermatol. 2022, 142, 124. 34242659 10.1016/j.jid.2021.06.010

[69] C. C. Deng , Y. F. Hu , D. H. Zhu , Q. Cheng , J. J. Gu , Q. L. Feng , L. X. Zhang , Y. P. Xu , D. Wang , Z. Rong , B. Yang , Nat. Commun. 2021, 12, 3709. 34140509 10.1038/s41467-021-24110-y PMC8211847

[70] C. F. Guerrero‐Juarez , P. H. Dedhia , S. Jin , R. Ruiz‐Vega , D. Ma , Y. Liu , K. Yamaga , O. Shestova , D. L. Gay , Z. Yang , K. Kessenbrock , Q. Nie , W. S. Pear , G. Cotsarelis , M. V. Plikus , Nat. Commun. 2019, 10, 650. 30737373 10.1038/s41467-018-08247-x PMC6368572

[71] V. Vorstandlechner , M. Laggner , D. Copic , K. Klas , M. Direder , Y. Chen , B. Golabi , W. Haslik , C. Radtke , E. Tschachler , K. Hotzenecker , H. J. Ankersmit , M. Mildner , Nat. Commun. 2021, 12, 6242. 34716325 10.1038/s41467-021-26495-2 PMC8556235

[72] K. De Bock , M. Georgiadou , S. Schoors , A. Kuchnio , B. W. Wong , A. R. Cantelmo , A. Quaegebeur , B. Ghesquiere , S. Cauwenberghs , G. Eelen , L. K. Phng , I. Betz , B. Tembuyser , K. Brepoels , J. Welti , I. Geudens , I. Segura , B. Cruys , F. Bifari , I. Decimo , R. Blanco , S. Wyns , J. Vangindertael , S. Rocha , R. T. Collins , S. Munck , D. Daelemans , H. Imamura , R. Devlieger , M. Rider , et al., Cell 2013, 154, 651. 23911327 10.1016/j.cell.2013.06.037

[73] A. R. Cantelmo , L. C. Conradi , A. Brajic , J. Goveia , J. Kalucka , A. Pircher , P. Chaturvedi , J. Hol , B. Thienpont , L. A. Teuwen , S. Schoors , B. Boeckx , J. Vriens , A. Kuchnio , K. Veys , B. Cruys , L. Finotto , L. Treps , T. E. Stav‐Noraas , F. Bifari , P. Stapor , I. Decimo , K. Kampen , K. De Bock , G. Haraldsen , L. Schoonjans , T. Rabelink , G. Eelen , B. Ghesquiere , J. Rehman , et al., Cancer Cell 2016, 30, 968. 27866851 10.1016/j.ccell.2016.10.006 PMC5675554

[74] L. S. Tombor , D. John , S. F. Glaser , G. Luxan , E. Forte , M. Furtado , N. Rosenthal , N. Baumgarten , M. H. Schulz , J. Wittig , E. M. Rogg , Y. Manavski , A. Fischer , M. Muhly‐Reinholz , K. Klee , M. Looso , C. Selignow , T. Acker , S. I. Bibli , I. Fleming , R. Patrick , R. P. Harvey , W. T. Abplanalp , S. Dimmeler , Nat. Commun. 2021, 12, 681. 33514719 10.1038/s41467-021-20905-1 PMC7846794

[75] S. Lovisa , E. Fletcher‐Sananikone , H. Sugimoto , J. Hensel , S. Lahiri , A. Hertig , G. Taduri , E. Lawson , R. Dewar , I. Revuelta , N. Kato , C. J. Wu , R. J. Bassett , N. Putluri , M. Zeisberg , E. M. Zeisberg , V. S. Lebleu , R. Kalluri , Sci Signal 2020, 13, aaz2597. 10.1126/scisignal.aaz2597 PMC7790440 32518142

[76] S. Cao , U. Yaqoob , A. Das , U. Shergill , K. Jagavelu , R. C. Huebert , C. Routray , S. Abdelmoneim , M. Vasdev , E. Leof , M. Charlton , R. J. Watts , D. Mukhopadhyay , V. H. Shah , J. Clin. Invest. 2010, 120, 2379. 20577048 10.1172/JCI41203 PMC2898590

[77] J. Zhang , J. Qiu , W. Zhou , J. Cao , X. Hu , W. Mi , B. Su , B. He , J. Qiu , L. Shen , Nat. Immunol. 2022, 23, 237. 35075279 10.1038/s41590-021-01097-8

[78] A. Abdullah , S. S. Akhand , J. Paez , W. Brown , L. Pan , S. Libring , M. Badamy , E. Dykuizen , L. Solorio , T. W. Andy , M. K. Wendt , Oncogene 2021, 40, 322. 33128042 10.1038/s41388-020-01530-6 PMC7808937

[79] J. Shim , S. J. Oh , E. Yeo , J. H. Park , J. H. Bae , S. H. Kim , D. Lee , J. H. Lee , J. Invest. Dermatol. 2022, 142, 2128. 35123990 10.1016/j.jid.2022.01.017

[80] J. Zhao , J. Patel , S. Kaur , S. L. Sim , H. Y. Wong , C. Styke , I. Hogan , S. Kahler , H. Hamilton , R. Wadlow , J. Dight , G. Hashemi , L. Sormani , E. Roy , M. C. Yoder , M. Francois , K. Khosrotehrani , Nat. Commun. 2021, 12, 2564. 33963183 10.1038/s41467-021-22717-9 PMC8105340

[81] J. Patel , B. Baz , H. Y. Wong , J. S. Lee , K. Khosrotehrani , J. Invest. Dermatol. 2018, 138, 1166. 29248546 10.1016/j.jid.2017.12.004

[82] S. Roy , A. K. Bag , R. K. Singh , J. E. Talmadge , S. K. Batra , K. Datta , Front. Immunol. 2017, 8, 1228. 29067024 10.3389/fimmu.2017.01228 PMC5641316

[83] S. E. Mcgowan , D. M. Mccoy , Am. J. Physiol. Lung Cell Mol. Physiol. 2021, 320, L179. 33174445 10.1152/ajplung.00149.2020

[84] Y. Li , Z. Wang , H. Xu , Y. Hong , M. Shi , B. Hu , X. Wang , S. Ma , M. Wang , C. Cao , H. Zhu , D. Hu , C. Xu , Y. Lin , G. Xu , Y. Yao , R. Zeng , Nat. Commun. 2024, 15, 5731. 38977708 10.1038/s41467-024-50121-6 PMC11231174

[85] Q. Li , S. Shao , Z. Zhu , J. Chen , J. Hao , Y. Bai , B. Li , E. Dang , G. Wang , J. Clin. Invest. 2023, 133, 160451. 10.1172/JCI160451 PMC10145935 36917196

[86] R. Ogawa , S. Akaishi , Med. Hypotheses 2016, 96, 51. 27959277 10.1016/j.mehy.2016.09.024

[87] S. Zhang , W. He , J. Dong , Y. K. Chan , S. Lai , Y. Deng , ACS Nano 2025, 19, 10922. 40071724 10.1021/acsnano.4c15743

[88] C. Liu , A. Somasundaram , S. Manne , A. M. Gocher , A. L. Szymczak‐Workman , K. M. Vignali , E. N. Scott , D. P. Normolle , E. J. Wherry , E. J. Lipson , R. L. Ferris , T. C. Bruno , C. J. Workman , D. A. A. Vignal , Nat. Immunol. 2020, 21, 1010. 32661362 10.1038/s41590-020-0733-2 PMC7442600

