# Supplementary material for: Targeting NRP1 in Endothelial Cells Facilitates the Normalization of Scar Vessels and Prevents Fibrotic Scarring
Source: Adv Sci (Weinh). 2025 Dec 8;13(13):e10545. doi: 10.1002/advs.202510545 (PMC12955901; doi:10.1002/advs.202510545)
Supplement: Supplementary file 1 — Supporting Information [file ADVS-13-e10545-s001.docx]

**Supplementary Materials for**

**Targeting NRP1 in Endothelial Cells Facilitates the Normalization of Scar Vessels and Prevents Fibrotic Scarring**

Yu Wang^1,3†^, Xin Zhou^1,2†^, Min Liu^1,5†^, Meimei Huang^4†^, Peirong Chen^1†^, Xinying Li^3^, Fangchao Xue^1^, Wenyan Zhao^1^, Di Liu^1^, Lang Li^1^, Yuangang Lu^3*^, Wen Zeng^1,2,6*^

^1^Department of Cell Biology, Army Medical University, Chongqing 400038, China.

^2^Jinfeng Laboratory, Chongqing 401329, China.

^3^Department of Plastic & Cosmetic Surgery, Daping Hospital, Army Medical University, Chongqing 400042, China.

^4^Department of Plastic Surgery, Southwest Hospital, Army Medical University, Chongqing 400038, China.

^5^Department of Neurology, Southwest Hospital, Army Medical University, Chongqing 400038, China.

^6^State Key Laboratory of Trauma, Burn and Combined Injury, Chongqing 400038, China.

*Corresponding author Email: skin515@tmmu.edu.cn; zengw0105@tmmu.edu.cn.

†These authors contributed equally to this work

**This PDF file includes:**

**Materials and Methods**

**Figures. S1 to S10**

**Tables S1 to S15**

**MATERIALS AND METHODS**

**Scanning Electron Microscopy Observation**

To evaluate the morphology of the scar vessels, a scanning electron microscopy assay was performed. After being cut into small pieces, scar tissues were fixed with electron microscopy fixative solution at 4℃ overnight. The second day, after being soaked in 50-100% acetone for dehydration, scar tissues were displaced with isoamyl acetate for more than 30 min and again treated with 100% acetone three times. Based on the critical-point drying method, the samples were dried and then fixed onto the objective table for metal coating. Images were obtained with a scanning electron microscope.

**Histological analysis and immunofluorescence staining**

Scar tissues were harvested and fixed with 4% PFA. Then, the tissues were dehydrated using graded ethanol, vitrified with dimethylbenzene, embedded in paraffin blocks, and cut into 5µm sections. After dewaxing and rehydration, the sections were stained with hematoxylin/eosin according to standard procedures. For immunofluorescence staining, the sections were first immersed in antigen repair solution under high temperature and pressure for antigen repair. Then, the sections were blocked with 5% goat serum, incubated at 4℃ overnight with primary antibody: anti-collagen I (1:100, Abclonal, #A5786), anti-CD31 (1:100, Abcam, #ab281583), anti-α-SMA (1:100, BOSTER, #BM0002), anti-α-SMA (1:100, BOSTER, #BM3902), anti-NRP1(1:100, Bioss, #bs-23865R),anti-NRP1(1:100, Bioss, #bsm-52479R),anti-CD31 (1:100, Abcam, #ab218), anti- VE-Cadherin (1:100, Immunoway, #YT5611), anti-FSP1 (1:100, Abclonal, #A19109), anti-Vimentin (1:100, MedChemExpress, #HY-P80371), anti-MYH11 (1:100, SANTA CRUZ , #sc-6956)and rinsed. Alexa-fluor 568, 647 or 488 was used (Invitrogen, #A11004, #A11001, #A32731, #A11011, #A21244), and the nuclei were back stained with DAPI (Beyotime, #C1002). The tissue sections were framed and photographed using a confocal fluorescence microscope (LSM800, Zeiss, Germany). All the immunofluorescence images were calculated with ImageJ software. For pericyte coverage quantification, vessels were counted manually in random regions, and the percentage of vessels covered with α-SMA+ pericytes = area of CD31+ blood vessels attached by α-SMA+ cells/total area of CD31+ blood vessels. The quantitative analysis of other vessels was carried out using the same method described above.

**Animal modeling and drug administration**

Scar animal models were constructed as previously reported. After the mice were anesthetized with 2% isoflurane, their back hair was removed. A full-thickness circular wound was created on the back of the mouse using a 6-millimeter-diameter punch. A 12-mm diameter silicone ring was secured around the wound with 6-0 sutures. The animals were divided randomly into 2 groups, one group was intraperitoneal injected with EG00229(NRP1-antagonist, MedChemExpress, #HY-10799) at 10 mg/kg every 2 days, another group was intraperitoneal injected with blank solvent as control (n=5). After 35 days, the scar area was photographed and detected. Then the scar and surrounding skin were collected and analyzed histologically.

**Evaluation of Hypoxia in Scar Tissue**

One hour after injection of Hypoxyprobe-1 (60 mg/kg, Hypoxyprobe™ Kit, Hypoxyprobe) into mice by tail vein, the mice were euthanized, and scar tissues were harvested for tissue hypoxia detection. Following the manufacturer's instructions, the tissue was embedded in paraffin and sectioned into 5µm slices for further staining. The hypoxic areas were quantitatively analyzed using ImageJ software.

**Cell culture**

Human umbilical vein endothelial cells (HUVECs) were purchased from Procell (China) and cultured in complete medium. The complete medium consisted of Dulbecco's Modified Eagle's Medium (DMEM, #C11995, Gibco), 10% (v/v) fetal bovine serum (FBS, #10099141, Gibco), 100U/ml penicillin and 100µg/ml streptomycin (#C0222, Beyotime). The cells were cultured at 37°C in a humidified incubator with 5% CO_2_.

**Knockdown and overexpression of NRP1 in HUVECs**

The siRNA kit was purchased from GenePharma, and specific sequences can be found in Table S4. Cells were digested and transferred into six-well plates. The transfection system was prepared according to the instructions and added to the wells. After 24 hours of transfection, the medium was changed, and TGF-β (Solarbio, #P02279) was added to continue induction. After the induction was complete, the cells were collected. Lentivirus overexpressing NRP1 was purchased from Heyuan Biotechnology Co., Ltd., and the specific plasmid information and sequences can be found in Table S5. Cells were digested and transferred into six-well plates. The transfection system was prepared according to the instructions and added to the wells. After 24 hours of transfection, the medium was changed, and puromycin at a concentration of 1:1000 was added. After 24 hours of treatment, the medium was changed again to obtain HUVECs overexpressing NRP1.

**Scratch assay**

Cells were seeded at 5× 10^5^ cells/well in 24-well plates. After growing to confluence, a 200 µl pipette tip was used to scratch a cross-shaped space in each well. The plates were gently washed with PBS three times, and the cells were cultured in FBS free culture medium supplemented with TGF-β (Solarbio, #P02279); the HUVECs were cultured at 37℃ with 5% CO_2_. Next, cells were photographed at 0 and 24 h. The relative migration rate of the cells was calculated based on the area at the 0 h time point.

**Endothelial Tube Formation Assay**

Cells were seeded at a density of 1×10⁴ cells per well in 24-well plates coated with basement membrane matrix and incubated at 37℃ for 8 hours. Images were acquired using a live-cell imaging workstation. The number of junctions, total length, and other parameters were calculated using ImageJ software.

**Western blot**

Protein was obtained from cells lysed or tissues with radio immunoprecipitation (RIPA) buffer (CST, #9806s) supplemented with 1× complete protease inhibitor (Sigma-Aldrich, #S8830), followed by sonication and centrifugation. The protein samples were separated by SDS-PAGE and transferred onto poly-vinylidene difluoride membranes (Merck, #IPVH0010). After blocking, the membranes were incubated with primary antibodies: anti-CD31 (1:800, Abcam, #ab281583), anti-α-SMA (1:1000, BOSTER, #BM3902), anti-NRP1(1:1000, Bioss, #bs-23865R),anti-NRP1(1:1000, Bioss, #bsm-52479R), anti-VE-Cadherin (1:800, Immunoway, #YT5611), anti-β-Actin (1:1500, Proteintech, #66009-1), anti-SMAD2 (1:1000, UpingBio, # YP-mAb-02015) overnight at 4℃. The membranes were then washed 3 times with TBST for 10 min, followed by incubation with secondary antibodies for 1 h. After additional washing, the protein signals were revealed using a Clarity™ Western ECL Substrate Kit (Bio-Rad, #1705060).

**Quantitative Real-Time Polymerase Chain Reaction**

RNA was extracted from cells using a RNeasy kit (Promega, #LS1040). RNA quality and concentration were assessed using A260 nm/A280 nm spectroscopy on a Nanodrop one (Thermo). Total RNA (1 μg) was used for cDNA synthesis with GoScript™ Reverse Transcriptase (Promega, #A5004) according to the manufacturer’s instructions. QPCR assays were performed with a Bio-Rad iQ5 Optical System and SYBR Green Master Mix (Promega, #A6001). Relative expression levels were calculated with the ΔΔCT method and β-Actin as the housekeeping gene. Sequences of the PCR primers used in this experiment are listed in Table S3.

**Molecular docking experiments for NRP1 and small peptides**

AlphaFold3 was used for protein structure prediction and construction. Paste the NRP1 sequence and the peptide sequence into the input box in AlphaFold3 to predict. Five parallels were made for each set of predictions. Finally, the structure with the highest pTM score was selected for structural analysis in PyMOL, and PyMOL 2.5.3 was used for drawing.

**Synthesis of PDA-NPs, PDA-TCR7-NPs, PDA-TCR7-ZIF8-NPs**

In a flask, 3.0 ml NH₃·H₂O (Aladdin, # A112079), 40 ml anhydrous ethanol (CHRON CHEMICALS, # 64-17-5), and 90 ml H_2_O were added sequentially. The reaction system was gently stirred at 30°C for 30 minutes, followed by the addition of a dopamine hydrochloride solution (0.5 g/10 ml, Aladdin, # D10311). The mixture was stirred continuously for 24 hours. Subsequently, PDA-NPs were collected by centrifugation and washed with anhydrous ethanol at least three times. The final product was dispersed in anhydrous ethanol (4 mg/ml) and stored in a refrigerator at 4°C. The PDA-NPs obtained in the previous step were placed in a Tris-HCl buffer solution (pH = 8.5) containing 2 mg/ml of TCR7 and stirred magnetically at room temperature for 0.5 hours. After centrifugation, PDA-TCR7-NPs were obtained. Under gentle stirring, 2-methylimidazole (1 ml, 1.32 M, Macklin, #693-98-1) was added to the PDA-TCR7-NPs solution (1.0 ml, 400 µg/ml). After 1 minute, zinc nitrate hexahydrate (RHAWN, #R051386) was added, and the mixture was stirred for an additional 5 minutes, followed by standing at room temperature for 3 hours. Finally, PDA-TCR7-ZIF8-NPs were collected by centrifugation after washing with water.

**Synthesis of PDA-TCR7-ZIF8-CS@GP**

The synthesized PDA-TCR7-ZIF8-NPs were redispersed in the solution of sodium β-glycerophosphate (500 mg/Ml, Aladdin, #D106347). Subsequently, a 3% (w/v) chitosan (BBI, #9012-76-4) solution was mixed with the above liquid under ice-water bath conditions until uniformly stirred, resulting in the PDA-TCR7-ZIF8-CS@GP hydrogel solution.

**Characterization of Synthesized Nanoparticles**

Transmission electron microscopy (FEI I, FEI Tecnai G2 Spirit 120 kV) was used to observe the structures of PDA-NPs and PDA-TCR7-ZIF8-NPs. The size of the nanoparticles was measured using a Zetasizer Nano ZS (Malvern). The UV−vis absorption spectra and infrared absorption spectra of the samples were detected using a Nanodrop one UV−vis spectrophotometer (Thermo) and an IS10 Fourier transform infrared (FTIR) spectrometer (Nicolet), respectively. For the photothermal characterization of different solutions, a 1064 nm laser was employed to irradiate aqueous solutions, and the temperature changes were monitored using a thermal imager (Hikmicro, H16).

**Assess the loading efficiency of TCR7 on PDA-NPs**

Five milliliters of Tris-HCl buffer (pH = 8.5) containing PDA-NPs (0.5 mg) and TCR7 (0.1 mg) were magnetically stirred at room temperature for 10 hours. Supernatant was collected at different time points (0-10 h), and the absorbance was measured at each time point to calculate the relative concentration of loaded TCR7.

**Assessment of the release efficiency of PDA-TCR7-NPs from PDA-TCR7-ZIF8-NPs**

PDA-TCR7-ZIF8-NPs were resuspended in buffers with pH=5.0 and pH=7.4, respectively, and incubated at room temperature for 14 hours. At two-hour intervals, a certain amount of the suspension was taken to measure the absorbance, and the concentration of PDA-TCR7-NPs was calculated.

**Photothermal-induced gelation of PDA-TCR7-ZIF8-CS@GP**

To compare the adhesive ability of PDA-TCR7-ZIF8-CS@GP on skin tissue with and without laser irradiation, it was first sprayed onto the surface of vertically placed artificial skin. One group was irradiated with a 1064 nm laser (power density of 1 W/cm²) for 20 seconds, while the other group was not exposed to near-infrared light. The gelation degree of the two groups was compared and photographed for record.

**The rheological property of hydrogel**

Temperature sweeps were performed on a HAAKE MARS 60 rheometer with a 20 mm parallel-plate geometry (gap 0.5 mm) at a constant frequency of 1 Hz and strain of 1 %. Samples were heated from 5 °C to 80 °C at 1 °C min⁻¹ under a solvent trap to minimize evaporation. The sol-to-gel transition temperature (Tgel) was defined as the crossover point where *G’ = G’’*.

**Cytotoxicity test**

HUVECs (10^4^ cells) were grown in 96-well plates.TCR7 (0, 5, 10, 15, 20, 25μg/mL) was added to different wells. CCK-8 was added after 24 h of incubation. The 96-well plates were incubated in an incubator protected from light for 2 h. Absorbance value at 450 nm was determined by a microplate reader.

**Hemolysis Assay**

Red blood cells (RBCs) were obtained by centrifuging rat blood at 1500 rpm for 10 min. RBCs were washed 5 times with PBS and resuspended in PBS to prepare a 2% (v/v) RBC suspension. Different concentration of TCR7 was added to RBC suspension. Triton X-100 (0.1%) was used as positive control, and PBS was used as a negative control. Samples were incubated at 37℃ for 2 h. The absorbance of the solutions was measured at 540 nm on a microplate reader. The hemolysis rate was calculated as follows: Hemolysis rate (%) = (sample-negative control)/(positive control-negative control)×100%.

**In vivo toxicity test**

TCR7 was administered to the mice via tail-vein injection. One day later, whole blood was collected for complete blood counts. Seven days after injection, the heart, liver, spleen, lung, and kidney were harvested, fixed, and embedded, followed by H&E staining.

**The degradation behavior of hydrogel**

The initial masses of the hydrogels after lyophilization were recorded as W0. They were then immersed in PBS at 37 °C. The hydrogels were removed at different time intervals and then lyophilized. The final weight of the degraded hydrogel was measured as Wt. Degradation rate (%) = (Wt -W0)/W0 ×100%

**In vitro adhesion of hydrogel**

The 150 μL of hydrogel was injected onto the artificial skin with NIR irradiation. Then the stresses induced by stretch, bend and twist were applied on the hydrogel to examine the adhesion properties on artificial skin.


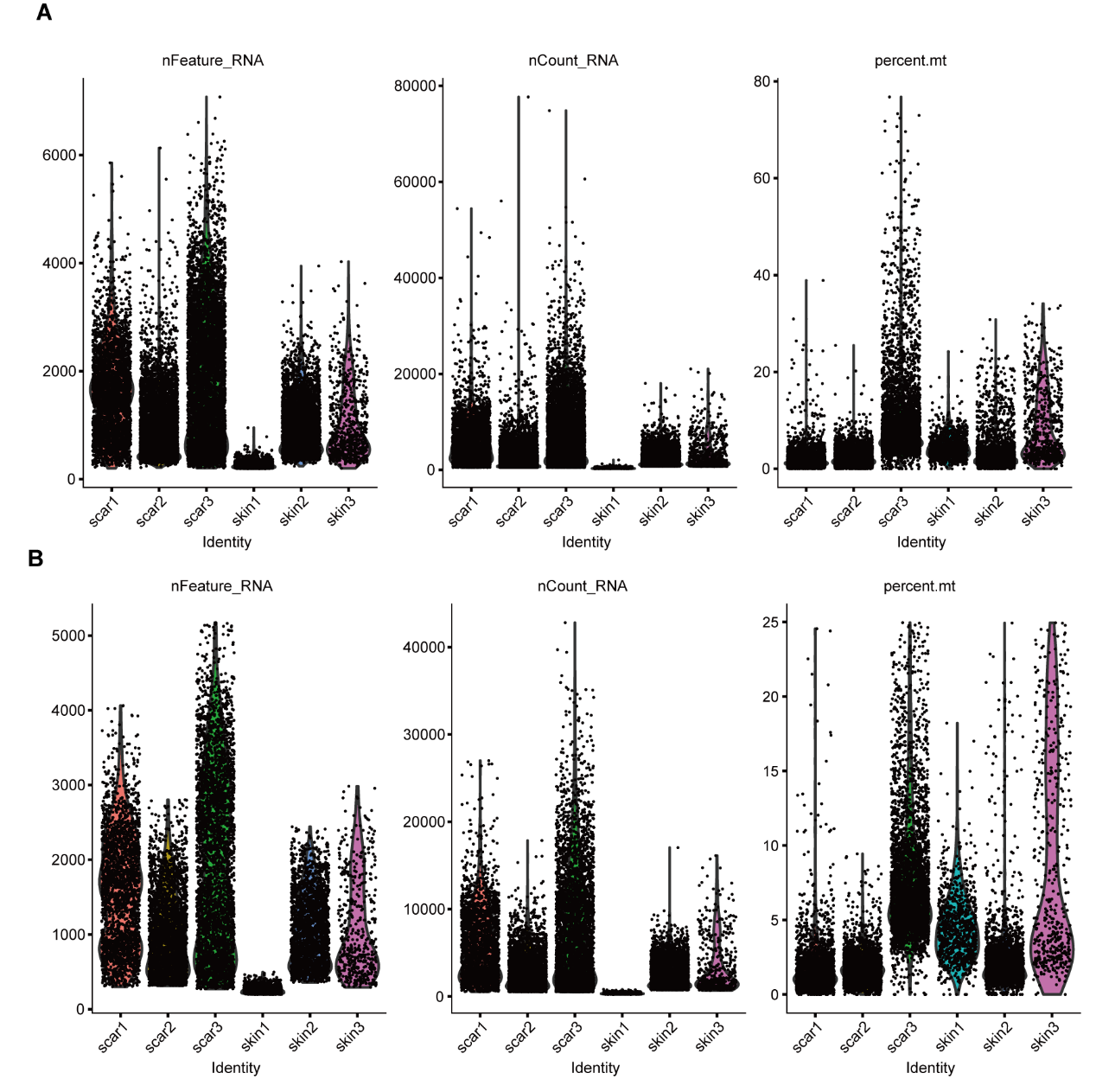


**Figure S1.** **Quality control on single-cell RNA sequencing data.** A) The raw data status of single-cell RNA sequencing data from three scar samples and three normal skins. B) The single-cell RNA sequencing data conducted with quality control.


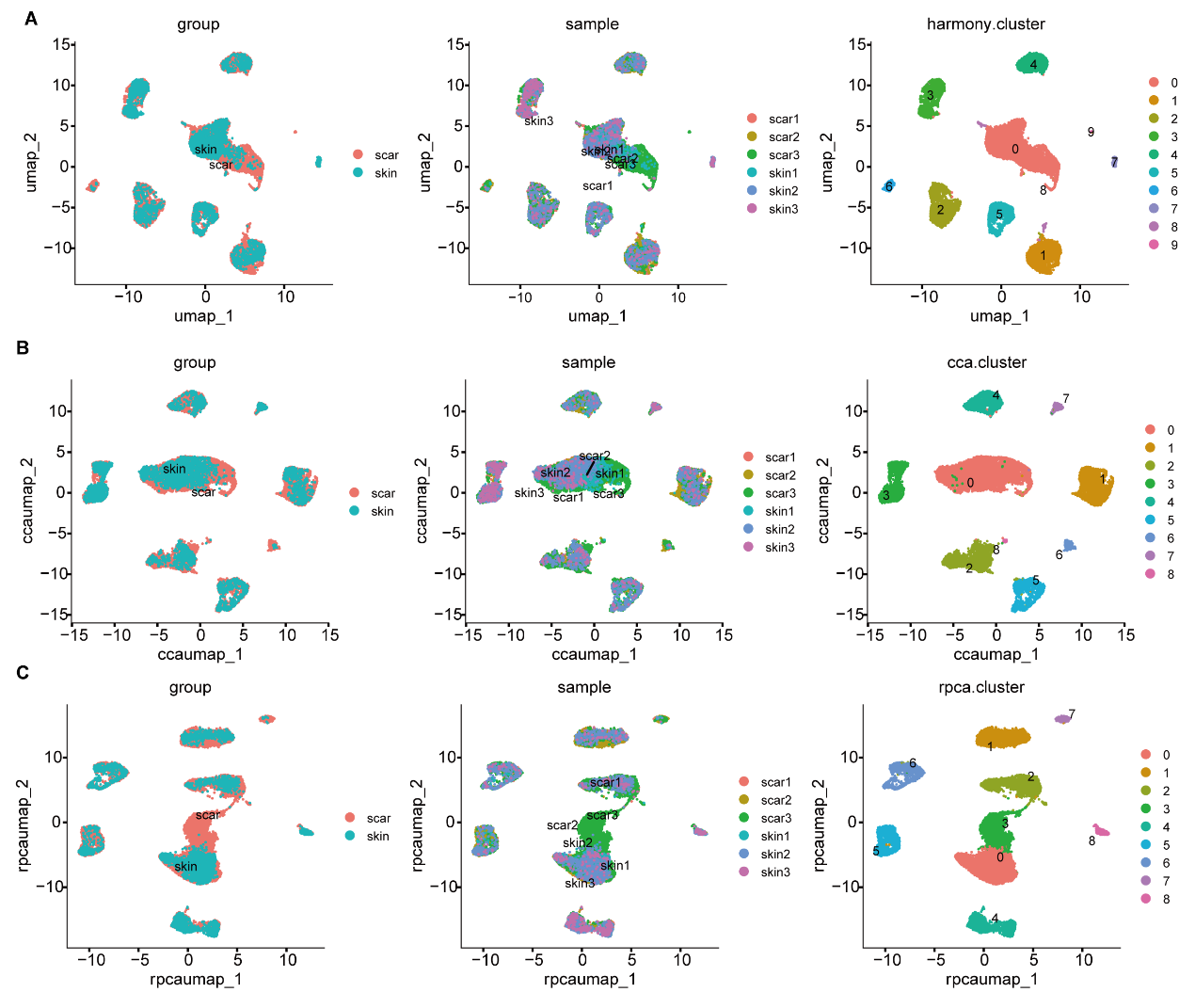


**Figure S2.** **Identification and assessment of batch effects in single-cell RNA sequencing data.** A)Identification and assessment of batch effects in single-cell RNA sequencing data using the Harmony algorithm. B)Identification and assessment of batch effects in single-cell RNA sequencing data using Canonical Correlation Analysis (CCA). C)Identification and assessment of batch effects in single-cell RNA sequencing data using Reciprocal Principal Component Analysis (RPCA).


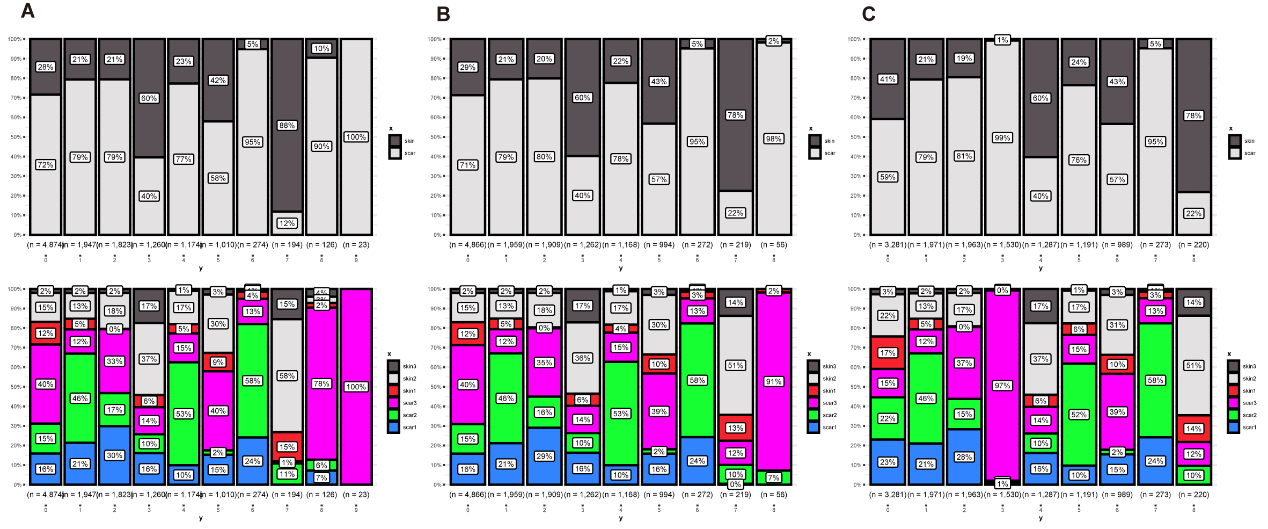


**Figure S3.** **Analysis of proportional variation differences between cell populations post-data integration.** A) Discrepancy bar plots of inter-group (top) and inter-sample (bottom) distributions in cell populations after Harmony integration. B) Discrepancy bar plots of inter-group (top) and inter-sample (bottom) distributions in cell populations after CCA integration. C) Discrepancy bar plots of inter-group (top) and inter-sample (bottom) distributions in cell populations after RPCA integration.


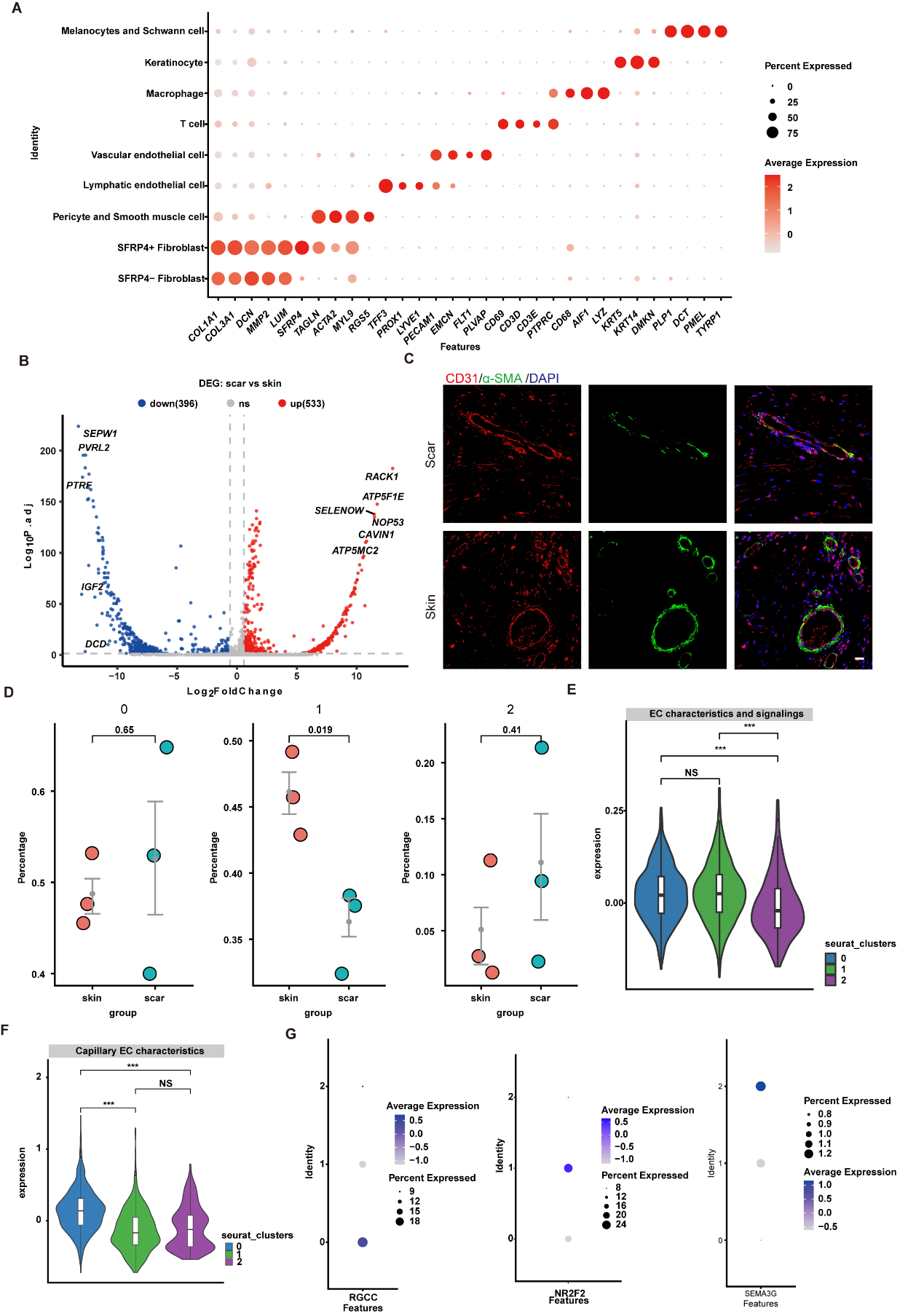


**Figure S4. Identification of cell types by marker genes and characteristics of ECs in human normal skin and scar.** A) Feature Plots showing the expression of discriminatory marker for each cell type in human normal skin and scar. B) Volcano plot of DEGs between the scar ECs and the normal skin ECs. Genes with log_2_ (fold change) > 0 are upregulated in scar ECs. C)Representative immunofluorescent images of vessels structure. CD31(red), α-SMA (green), DAPI (blue). Scale bars, 20 µm. D) Proportion of different EC subclusters in scar tissues and normal skin. E) Violin plots showing the analysis of EC characteristics and signaling in different EC subclusters. F) Violin plots showing the analysis of capillary EC characteristics and signaling in different EC subclusters. G) Expression of *RGCC*, *NR2F2* and *SEMA3G* in different EC subclusters.


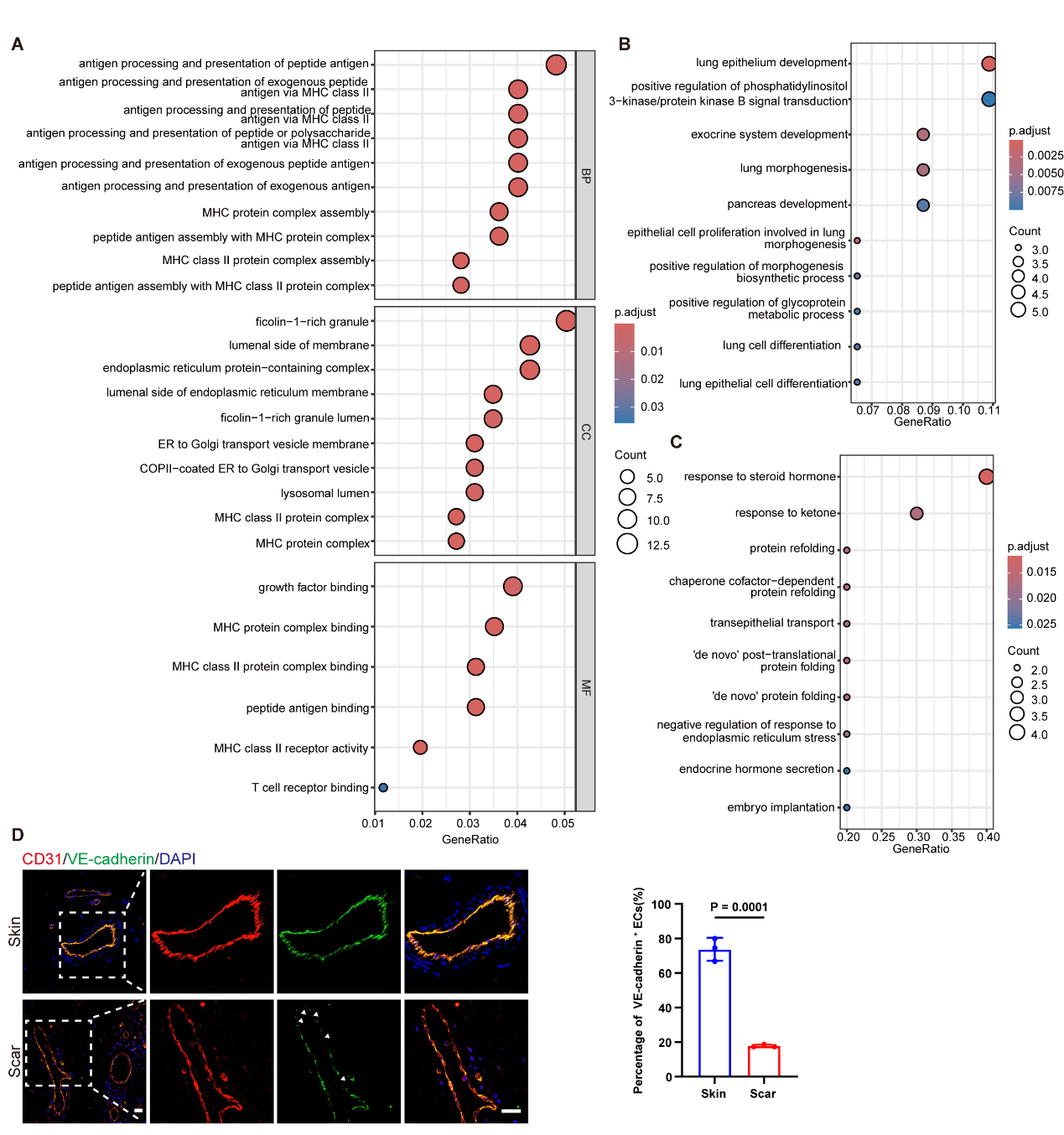


**Figure S5. Characteristics of NRP1^high^ ECs.** A) GO functional enrichment of differential genes between *NRP1*^high^ECs and *NRP1*^low^ ECs. B) GO functional enrichment of differential genes which are mostly downregulated along the pseudotime axis. C) GO functional enrichment of differential genes which are mostly upregulated along the pseudotime. D) Immunofluorescent staining of VE-cadherin(green), CD31(red) and DAPI (blue) in human normal skin and scar tissue. The white dotted lines indicate vessels, and the triangles indicate the ECs loss the expression of VE-cadherin. Quantification of the percentage of VE-cadherin^+^ ECs(*n=3*). Scale bars, 20µm.


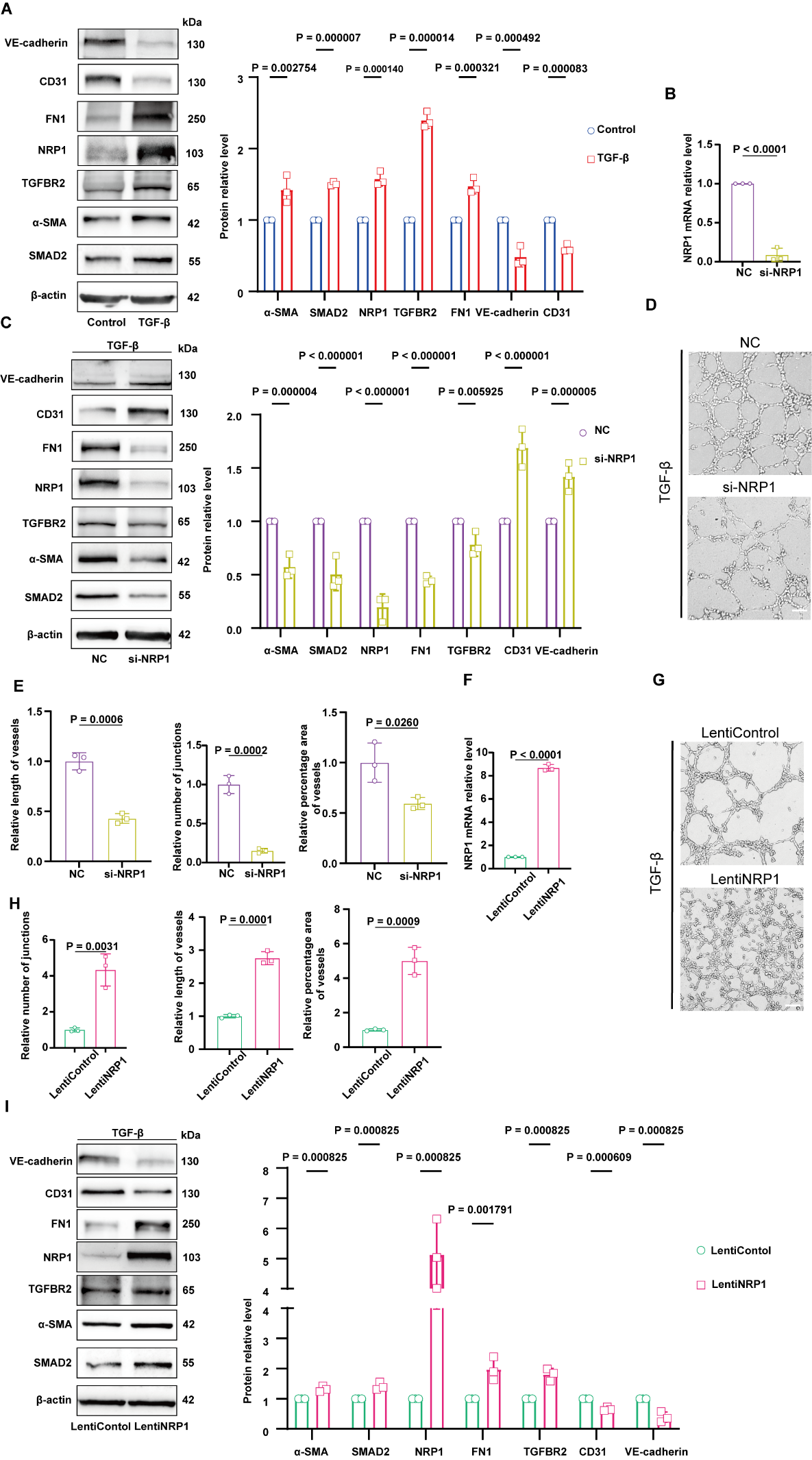


**Figure S6. NRP1 engages TGF-β induced EndMT by SMAD2.** A) Western blot and quantification of NRP1, VE-cadherin, CD31, α-SMA, SMAD2，TGFBR2, FN1 expression in HUVECs induced by TGF-β (*n* = 3). B) Detection of transcript levels of *NRP1* in HUVECs transfected with siRNA (*n* = 3). C) Western blot and quantification of NRP1, VE-cadherin, CD31, α-SMA, SMAD2, TGFBR2, FN1expression in HUVECs transfected with siRNA and induced by TGF-β (*n* = 3). D-E) Quantification of tube formation in HUVECs treated with TGF-β and transfected with siRNA (*n* = 3). F) Detection of transcript levels of *NRP1* in HUVECs transfected with lentivirus (*n* = 3). G-H) Quantification of tube formation in HUVECs treated with TGF-β and transfected with lentivirus (*n* = 3). I)Western blot and quantification of NRP1, VE-cadherin, CD31, α-SMA, SMAD2, TGFBR2, FN1expression in HUVECs overexpressed with NRP1 and induced by TGF-β (*n* = 3). Statistical significance was analyzed by unpaired two-tailed Student’s *t*-test.


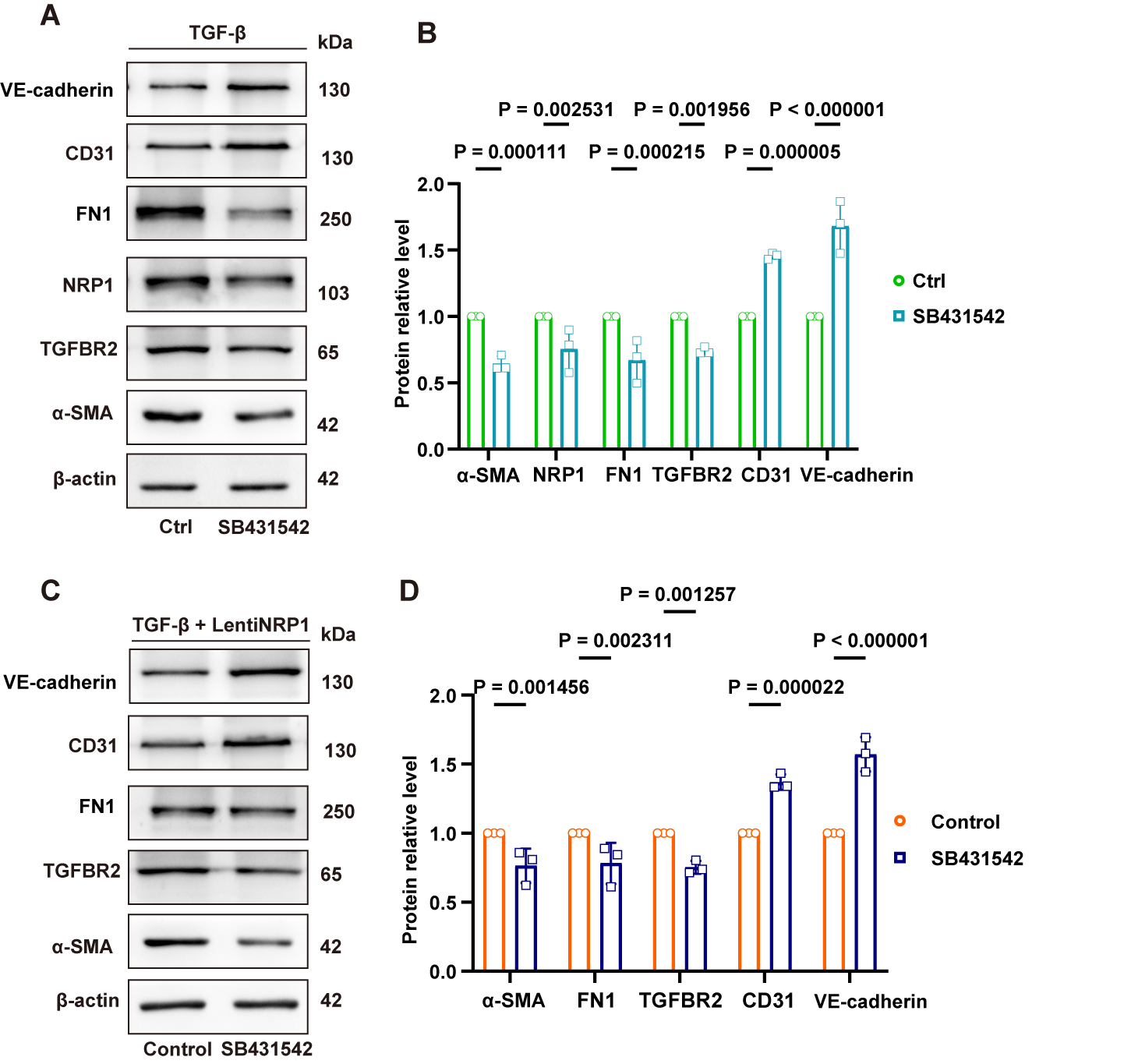


**Figure S7. The mechanism between NRP1 and TGF-β/SMAD2 signal pathway in EndMT.** A-B) Western blot and quantification of NRP1, VE-cadherin, CD31, α-SMA, TGFBR2, FN1 expression in EndMT cells treated with SB431542 or vehicle Ctrl (*n* = 3). C-D) Western blot and quantification of VE-cadherin, CD31, α-SMA, TGFBR2, FN1 expression in overexpressing NRP1 HUVECs treated with SB431542 or vehicle Control (*n* = 3). Statistical significance was analyzed by unpaired two-tailed Student’s *t*-test.


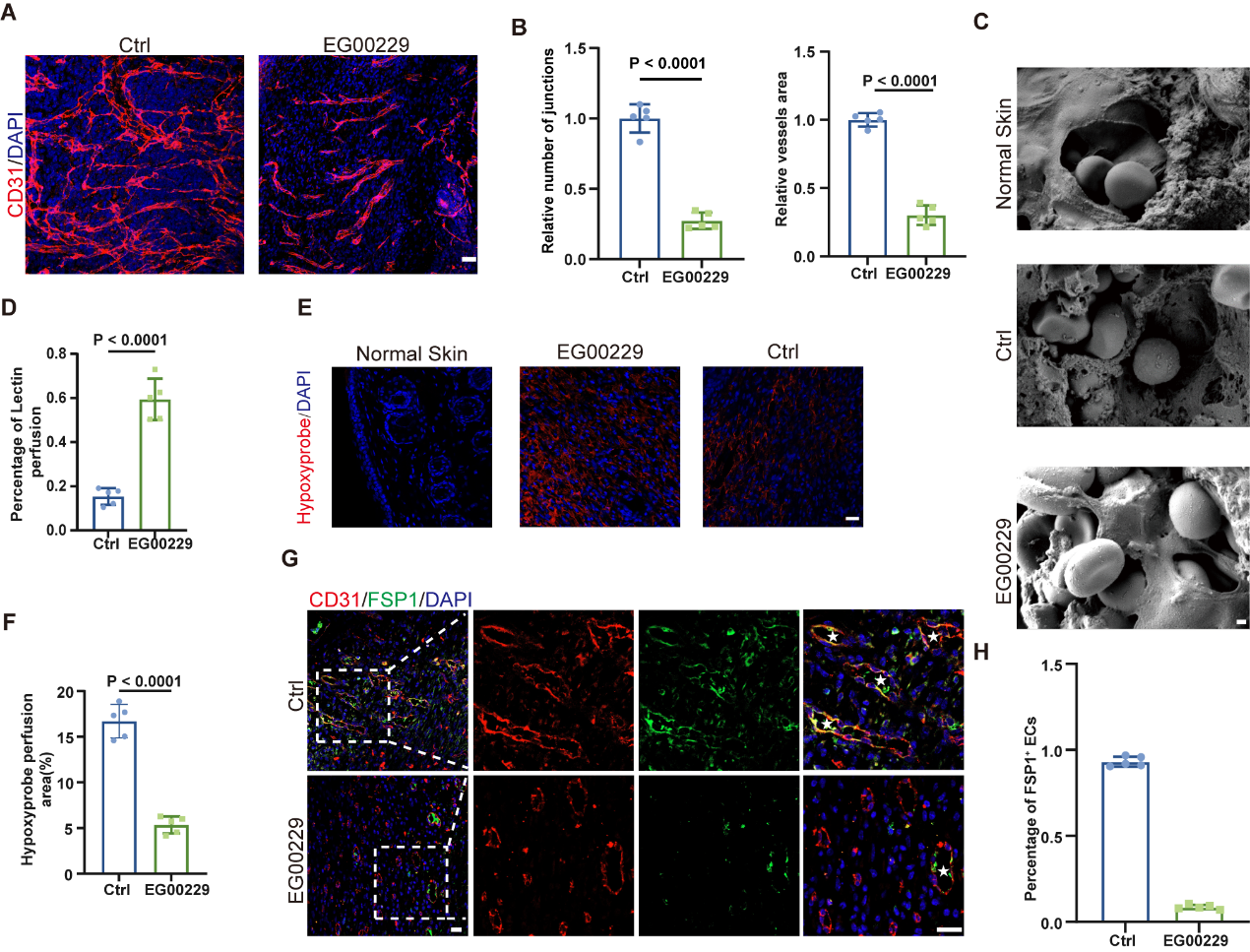


**Figure S8. Inhibition of NRP1 restores vessels and impairs EndMT in wounds.** A) Immunofluorescent staining of CD31(red) and DAPI (blue) in mice wound tissue treated with EG00229 or vehicle Ctrl on the 7^th^ day. Scale bars, 20 µm. B) Quantification of vessel junction and area in mice wound tissue treated with EG00229 or not on the 7^th^ day (*n* = 5). C) Representative SEM images of vessels in mice normal skin, wound tissue treated with EG00229 or vehicle Ctrl on the 7^th^ day. Scale bars, 200 nm. D) Quantification of Lectin perfused vessels in mice normal skin and wound tissue treated with EG00229 or Ctrl on the 7^th^ day (*n* = 5). E-F) Immunofluorescent image and quantification of Hypoxyprobe perfusion area in mice normal skin, wound tissue treated with EG00229 or Ctrl on the 7^th^ day (*n* = 5). Hypoxyprobe (red), DAPI (blue). Scale bars, 50 µm. G) Immunofluorescent staining of FSP1(green), CD31(red) and DAPI (blue) in mice wound tissue treated with EG00229 or Ctrl on the 14^th^ day. The white dotted lines indicate vessels, and the stars indicate the ECs expressed FSP1. Scale bars, 20 µm. H) Quantification of percentage of FSP1+ ECs in mice wound tissue treated with EG00229 or Ctrl on the 14^th^ day (*n* = 5). Statistical significance was analyzed by unpaired two-tailed Student’s *t*-test.


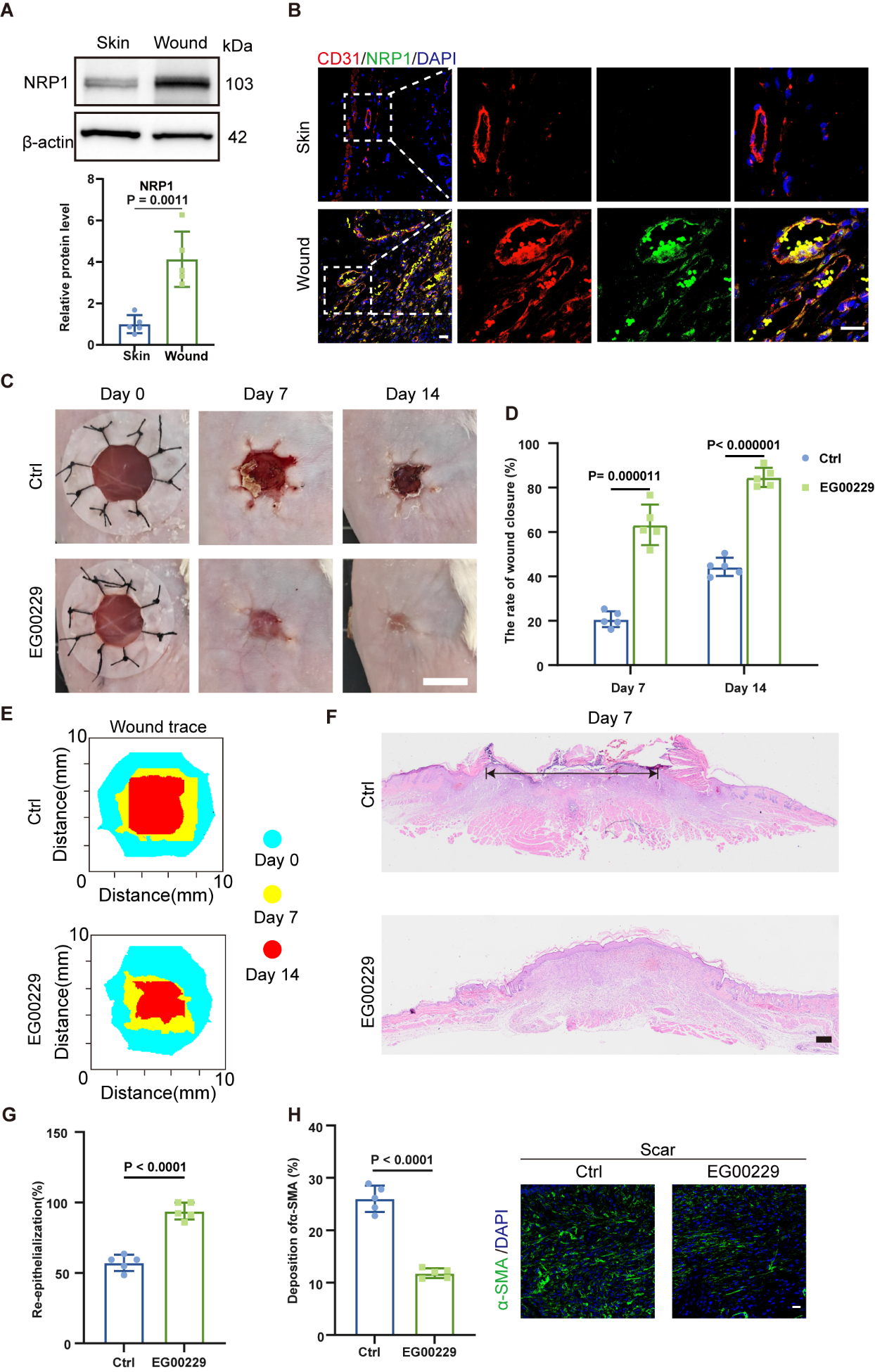


**Figure S9. Inhibition of NRP1 accelerated wound healing.** A) Western blot and quantification of expression of NRP1 in mice wound tissue and normal skin on the 7^th^ day (*n* = 5). B) Immunofluorescent staining of NRP1(green), CD31(red), DAPI (blue) in mice wound tissue and normal skin. The white dotted lines indicate the area of vessels. Scale bars, 20 µm. C) Representative images of wounds in mice treated with EG00229 or vehicle Ctrl on the 7^th^ and 14^th^ day. Scale bars, 5 mm. D) Quantification of wound closure rate in mice scar tissue treated with EG00229 or Ctrl on the 7^th^ day (*n* = 5). E) The simulation of wound healing in mice treated with EG00229 or Ctrl on the 7^th^ and 14^th^ day. F) Representative images of H&E-stained scar treated with EG00229 or Ctrl on the 7^th^ day. The black arrow indicates the area without epithelium covered. Scale bars, 200 µm. G) Quantification of re-epithelialization in mice scar tissue treated with EG00229 or Ctrl on the 7^th^ day (*n* = 5). H) Representative immunofluorescent images and quantification of α-SMA (green) deposition in scar tissue treated with EG00229 or Ctrl on the 35^th^ day. (*n* = 5). Statistical significance was analyzed by unpaired two-tailed Student’s *t*-test.


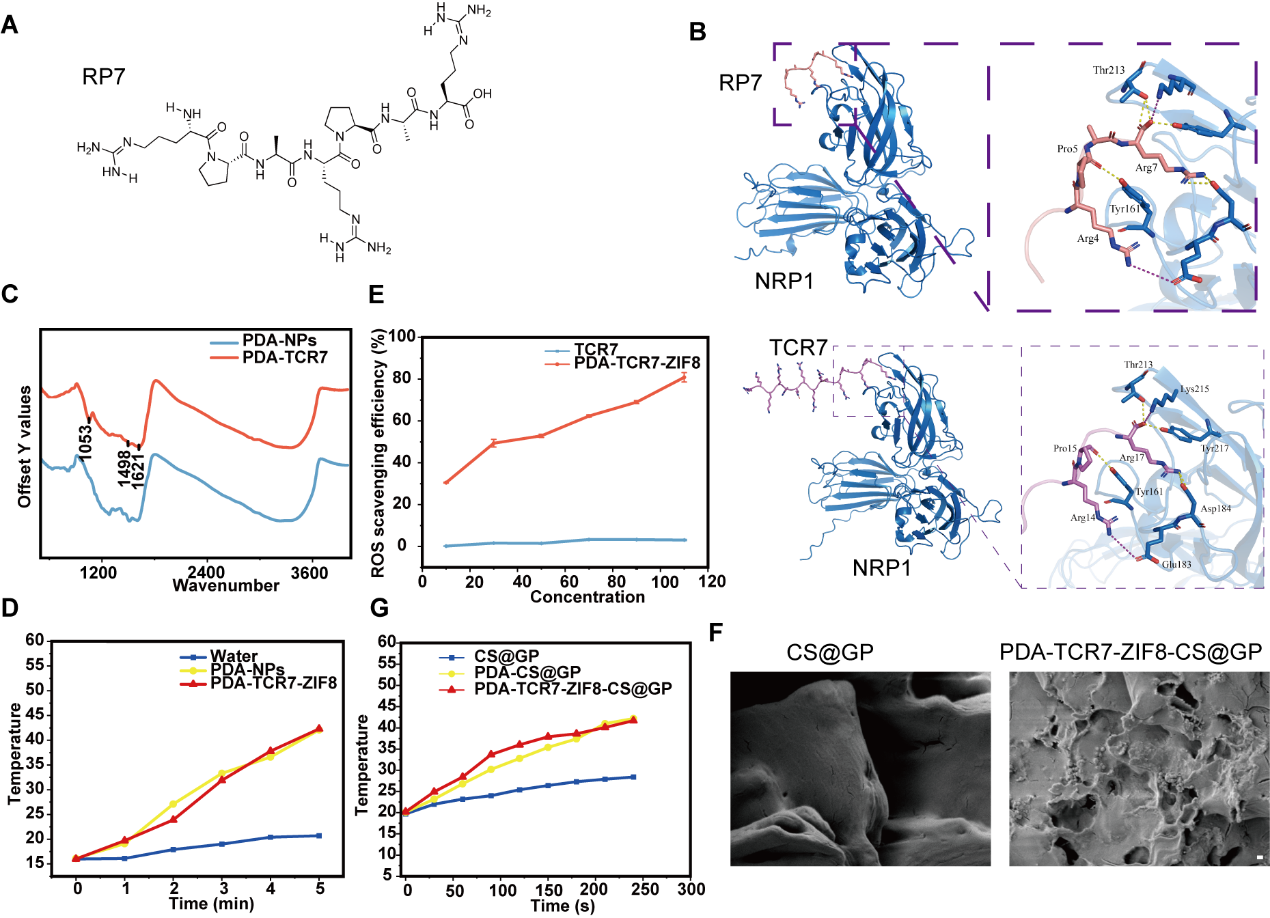


**Figure S10. Characterization of PDA-TCR7-ZIF8-CS@GP hydrogel.** A) Molecular structure of RP7 peptide. B) 3D combined diagram of Docking results of RP7 and TCR7 on NRP1 proteins. All the yellow dotted lines are hydrogen bonds, and all the purple dotted lines are electrostatic interactions. C) Fourier transform infrared spectroscopy (FTIR)spectra of PDA-NPs and PDA-TCR7-NPs. D) Quantification of the temperature increment of PDA-NPs and PDA-TCR7-ZIF8-NPs. E) Quantification of ROS scavenging efficiency in different concentrations of PDA-TCR7-ZIF8-NPs (*n*=3). F) Representative scanning electron microscope images of CS@GP and PDA-TCR7-ZIF8-CS@GP hydrogel. Scale bars, 200nm. G) Quantification of the temperature increment of CS@GP, PDA-CS@GP and PDA-TCR7-ZIF8-CS@GP hydrogel. Statistical significance was analyzed by unpaired two-tailed Student’s *t*-test.


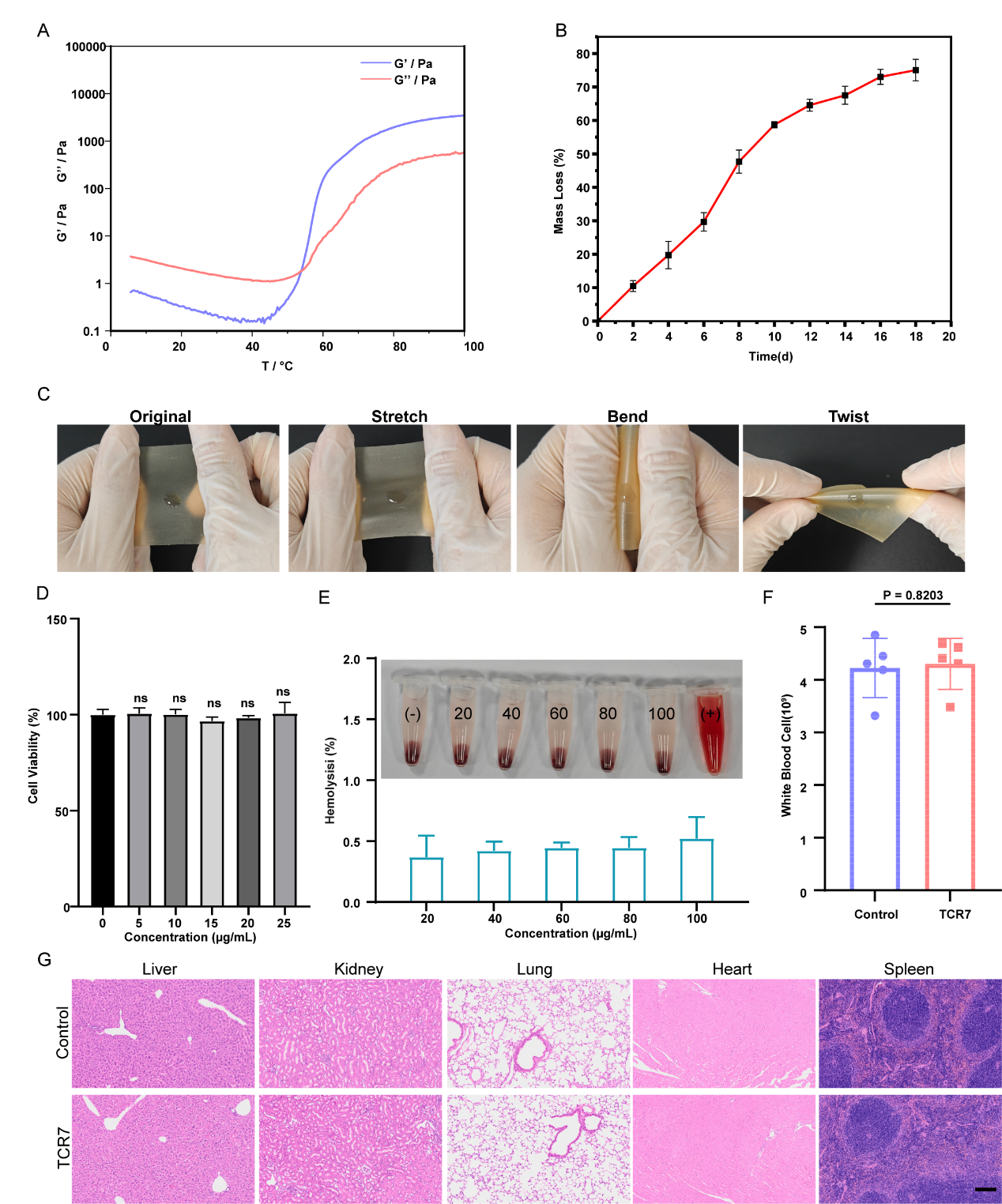


**Figure S11. *In vivo* and *in vitro* biocompatibility of TCR7.** A) Rheological properties of the PDA-TCR7-ZIF8-CS@GP hydrogel. B) Degradation rates of PDA-TCR7-ZIF8-CS@GP hydrogel. C) Photographs of PDA-TCR7-ZIF8-CS@GP hydrogel adhered to artificial skin following stretching, bending, and twisting. D) Cell viability of HUVECs after treatment with different concentrations of TCR7. E) Hemolysis rate and images of hemolysis of TCR7 at different concentrations. F) Quantification of white blood cell in mice injected with TCR7 or Control (normal saline) through tail veins for 1day (*n=5*). G) H&E staining of liver, kidney, lung, heart, spleen of mice injected with TCR7 or Control (normal saline) through tail vein for 7days. Scale bars, 100 µm. Statistical significance was analyzed by unpaired two-tailed Student’s *t*-test.


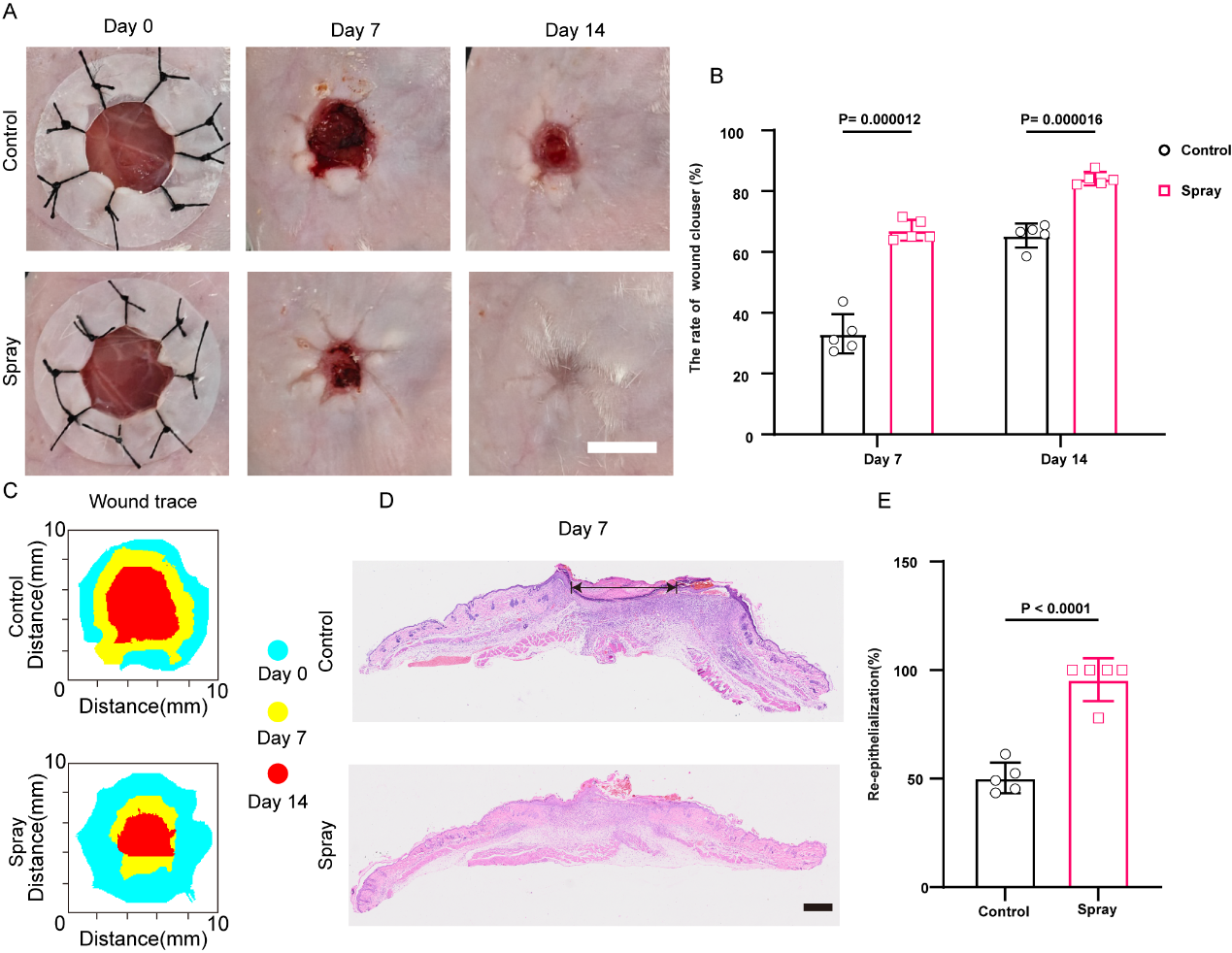


**Figure S12. PDA-TCR7-ZIF8-CS@GP hydrogel spray accelerated wound healing.** A) Representative images of wounds in mice treated with spray or control (PBS) on the 7^th^ and 14^th^ day. Scale bars, 5 mm. B) Quantification of wound closure rate in mice scar tissues treated with spray or control on the 7^th^ day (*n* = 5). C) The simulation of wound healing in mice treated with spray or control on the 7^th^ and 14^th^ day. D) Representative images of H&E-stained scar treated with spray or control on the 7^th^ day. The black arrow indicates the area without epithelium covered. Scale bars, 200 µm. E) Quantification of re-epithelialization in mice scar tissue treated with spray or control on the 7^th^ day (*n* = 5). Statistical significance was analyzed by unpaired two-tailed Student’s t-test.


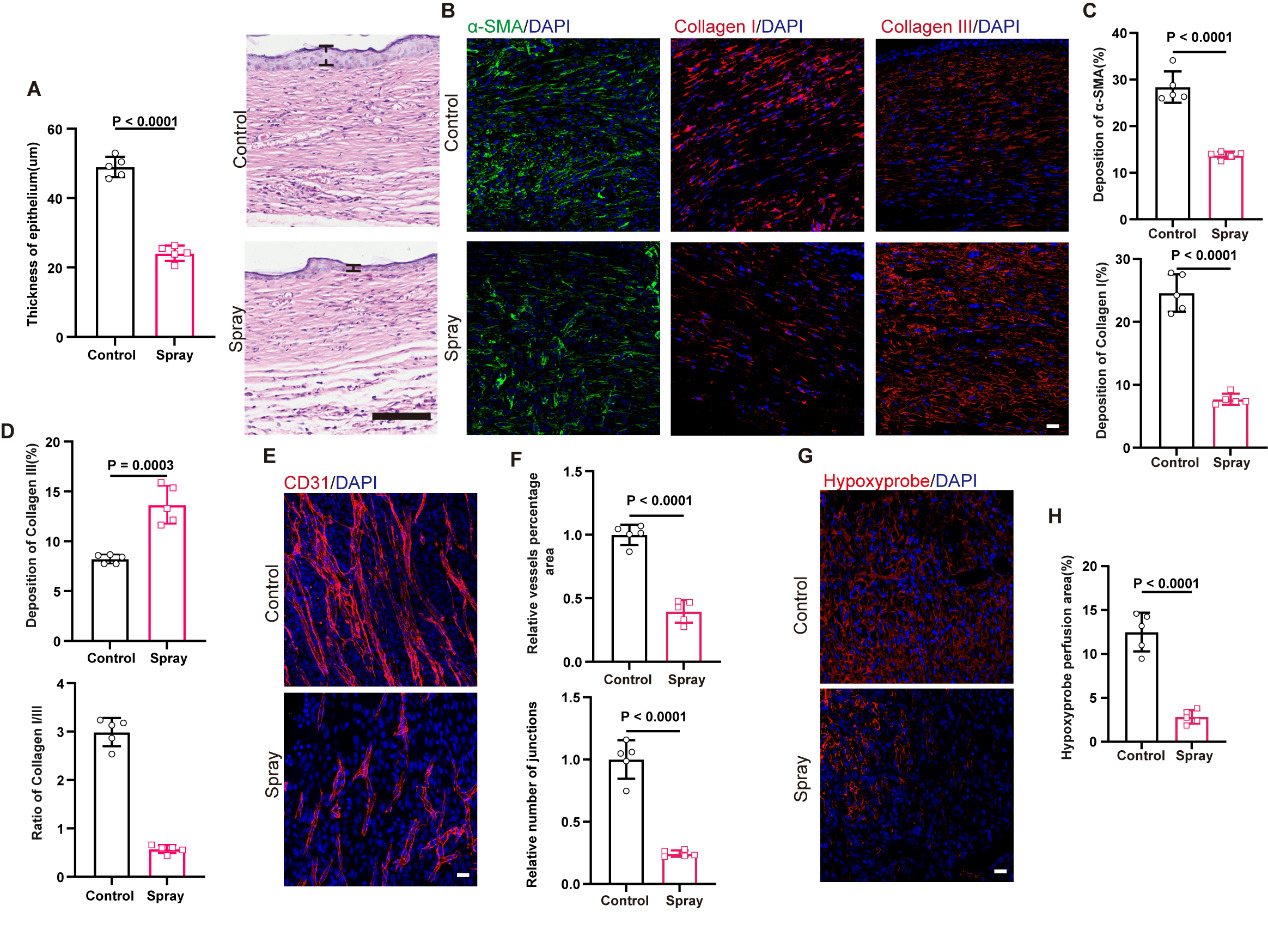


**Figure S13. PDA-TCR7-ZIF8-CS@GP hydrogel spray could normalize the function and structure of vessels, alleviating scar formation.** A) Quantification and representative images of skin epidermis thickness in mice scar tissue treated with spray or control (PBS) on the 35^th^ day (*n* = 5). The black dotted lines indicate the area of epidermis. Scale bars, 100 µm. B) Representative immunofluorescent images of α-SMA (green), Collagen I (red), Collagen III (red)deposition in mice scar tissues treated with spray or control on the 35^th^ day (*n* = 5). Scale bars, 20 µm. C) Quantification of α-SMA (green), Collagen I (red) deposition in mice scar tissues treated with spray or control on the 35^th^ day (*n* = 5). D) Quantification of Collagen III (red) deposition and ratio of Collagen I / III in mice scar tissues treated with spray or control on the 35^th^ day (*n* = 5). E-F) Immunofluorescent staining of CD31(red) and DAPI (blue) in mice scar tissue treated with spray or control on the 35^th^ day. Quantification of vessel junction and area in mice scar tissue treated with spray or control on the 35^th^ day (*n* = 5). Scale bars, 20 µm. G-H) Representative immunofluorescent images and quantification of Hypoxyprobe in scar tissues treated with spray or control on the 7^th^ day. Hypoxyprobe (red), DAPI (blue) (*n* = 5). Scale bars, 20 µm. Statistical significance was analyzed by unpaired two-tailed Student’s *t*-test.


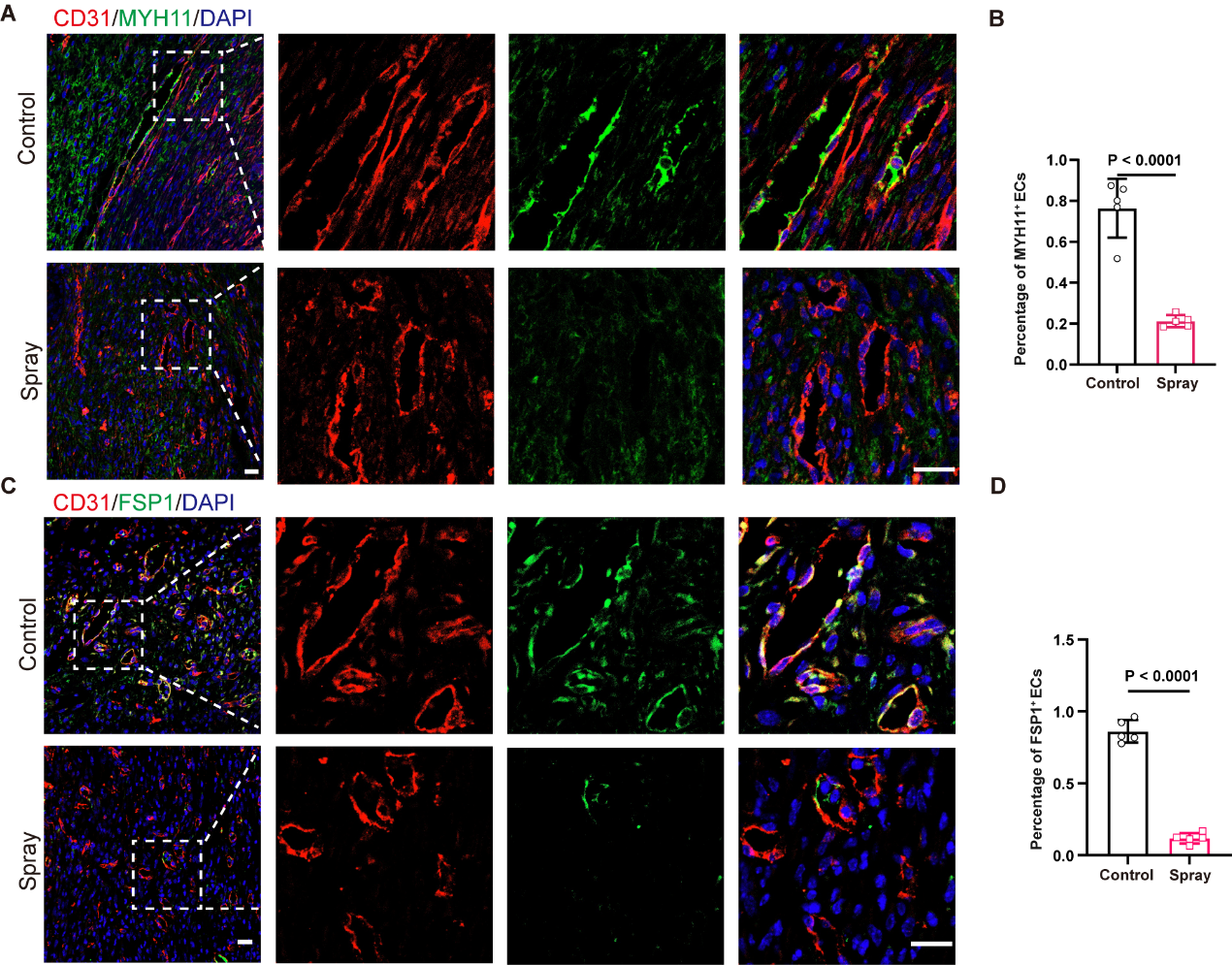


**Figure S14. PDA-TCR7-ZIF8-CS@GP hydrogel sprayer could impair EndMT in wound.** A) Immunofluorescent staining of MYH11(green), CD31(red) and DAPI (blue) in mice wound tissue treated with spray on the 14^th^ day. The white dotted lines indicate the ECs expressed MYH11. Scale bars, 20μm. B) Quantification of the percentage of MYH11^+^ ECs in mice wound tissue treated with spray or PBS on the 14^th^ day (*n* = 5). D) Immunofluorescent staining of FSP1(green), CD31(red) and DAPI (blue) in mice wound tissue treated with spray on the 14^th^ day. The white dotted lines indicate the ECs expressed FSP1. Scale bars, 20μm. E) Quantification of the percentage of FSP1^+^ ECs in mice wound tissue treated with spray on the 14^th^ day (*n* = 5). Statistical significance was analyzed by unpaired two-tailed Student’s *t*-test.

**Table S1: Detailed information of clinical samples for single cell sequencing analysis.**

| Sample | Data Accession No. | Sex | Age | Group | Location | Etiology |
| --- | --- | --- | --- | --- | --- | --- |
| Skin1 | GSM4729097 | Female | 30 | Skin | Abdomen | Healthy skin |
| Skin2 | GSM4729098 | Female | 36 | Skin | Abdomen | Healthy skin |
| Skin3 | GSM4729099 | Female | 43 | Skin | Abdomen | Healthy skin |
| Scar1 | GSM4729100 | Female | 75 | Scar | Forearm | Injury |
| Scar2 | GSM4729101 | Male | 24 | Scar | Calf | Burn |
| Scar3 | GSM4729102 | Female | 54 | Scar | Axilla | Surgical |

**Table S2: Detailed information of clinical samples for experiments.**

| **Sample** | **Sex** | **Age** | **Height**  **(cm)** | **Weight**  **(kg)** | **Race** | **Group** | **Location** | **Medical history** | **Etiology** |
| --- | --- | --- | --- | --- | --- | --- | --- | --- | --- |
| Skin1 | Female | 37 | 153 | 49 | Asia | Skin | Abdomen | None | Health skin |
| Skin2 | Female | 31 | 155 | 50 | Asia | Skin | Abdomen | None | Health skin |
| Skin3 | Female | 31 | 155 | 50 | Asia | Skin | Abdomen | None | Health skin |
| Skin4 | Female | 34 | 155 | 50 | Asia | Skin | Abdomen | None | Health skin |
| Scar1 | Female | 34 | 160 | 50 | Asia | Scar | Abdomen | None | Burn |
| Scar2 | Female | 32 | 150 | 47.5 | Asia | Scar | Abdomen | None | Surgical scar |
| Scar3 | Female | 30 | 160 | 70 | Asia | Scar | Abdomen | None | Surgical scar |
| Scar4 | Female | 16 | 168 | 59 | Asia | Scar | Abdomen | None | Burn |

**Table S3: Primer sequences used for qPCR analysis.**

| Gene | Forward | Reverse |
| --- | --- | --- |
| PECAM1 | AACAGTGTTGACATGAAGAGCC | TGTAAAACAGCACGTCATCCTT |
| SMAD2 | CGTCCATCTTGCCATTCA | CTCAAGCTCATCTAATCGTCCTG |
| CDH5 | TTGGAACCAGATGCACATTGAT | TCTTGCGACTCACGCTTGAC |
| FN1 | CGGTGGCTGTCAGTCAAAG | AAACCTCGGCTTCCTCCATAA |
| NRP1 | GGCGCTTTTCGCAACGATAAA | TCGCATTTTTCACTTGGGTGAT |
| β-ACTIN | GACTTAGTTGCGTTACACCCTTTCT | GCTGTCACCTTCACCGTTCC |
| ACTA2 | AAAAGACAGCTACGTGGGTGA | GCCATGTTCTATCGGGTACTTC |

**Table S4: Sequences of siRNA used in this study.**

| Name | Forward | Reverse |
| --- | --- | --- |
| si-NRP1 | CCUGAAUGUUCCCAGAACUTT | AGUUCUGGGAACAUUCAGGTT |

**Table S5: Sequences of NRP1 used in plasmid.**

| ATGGAGAGGGGGCTGCCGCTCCTCTGCGCCGTGCTCGCCCTCGTCCTCGCCCCGGCCGGCGCTTTTCGCAACGATAAATGTGGCGATACTATAAAAATTGAAAGCCCCGGGTACCTTACATCTCCTGGTTATCCTCATTCTTATCACCCAAGTGAAAAATGCGAATGGCTGATTCAGGCTCCGGACCCATACCAGAGAATTATGATCAACTTCAACCCTCACTTCGATTTGGAGGACAGAGACTGCAAGTATGACTACGTGGAAGTCTTCGATGGAGAAAATGAAAATGGACATTTTAGGGGAAAGTTCTGTGGAAAGATAGCCCCTCCTCCTGTTGTGTCTTCAGGGCCATTTCTTTTTATCAAATTTGTCTCTGACTACGAAACACATGGTGCAGGATTTTCCATACGTTATGAAATTTTCAAGAGAGGTCCTGAATGTTCCCAGAACTACACAACACCTAGTGGAGTGATAAAGTCCCCCGGATTCCCTGAAAAATATCCCAACAGCCTTGAATGCACTTATATTGTCTTTGTGCCAAAGATGTCAGAGATTATCCTGGAATTTGAAAGCTTTGACCTGGAGCCTGACTCAAATCCTCCAGGGGGGATGTTCTGTCGCTACGACCGGCTAGAAATCTGGGATGGATTCCCTGATGTTGGCCCTCACATTGGGCGTTACTGTGGACAGAAAACACCAGGTCGAATCCGATCCTCATCGGGCATTCTCTCCATGGTTTTTTACACCGACAGCGCGATAGCAAAAGAAGGTTTCTCAGCAAACTACAGTGTCTTGCAGAGCAGTGTCTCAGAAGATTTCAAATGTATGGAAGCTCTGGGCATGGAATCAGGAGAAATTCATTCTGACCAGATCACAGCTTCTTCCCAGTATAGCACCAACTGGTCTGCAGAGCGCTCCCGCCTGAACTACCCTGAGAATGGGTGGACTCCCGGAGAGGATTCCTACCGAGAGTGGATACAGGTAGACTTGGGCCTTCTGCGCTTTGTCACGGCTGTCGGGACACAGGGCGCCATTTCAAAAGAAACCAAGAAGAAATATTATGTCAAGACTTACAAGATCGACGTTAGCTCCAACGGGGAAGACTGGATCACCATAAAAGAAGGAAACAAACCTGTTCTCTTTCAGGGAAACACCAACCCCACAGATGTTGTGGTTGCAGTATTCCCCAAACCACTGATAACTCGATTTGTCCGAATCAAGCCTGCAACTTGGGAAACTGGCATATCTATGAGATTTGAAGTATACGGTTGCAAGATAACAGATTATCCTTGCTCTGGAATGTTGGGTATGGTGTCTGGACTTATTTCTGACTCCCAGATCACATCATCCAACCAAGGGGACAGAAACTGGATGCCTGAAAACATCCGCCTGGTAACCAGTCGCTCTGGCTGGGCACTTCCACCCGCACCTCATTCCTACATCAATGAGTGGCTCCAAATAGACCTGGGGGAGGAGAAGATCGTGAGGGGCATCATCATTCAGGGTGGGAAGCACCGAGAGAACAAGGTGTTCATGAGGAAGTTCAAGATCGGGTACAGCAACAACGGCTCGGACTGGAAGATGATCATGGATGACAGCAAACGCAAGGCGAAGTCTTTTGAGGGCAACAACAACTATGATACACCTGAGCTGCGGACTTTTCCAGCTCTCTCCACGCGATTCATCAGGATCTACCCCGAGAGAGCCACTCATGGCGGACTGGGGCTCAGAATGGAGCTGCTGGGCTGTGAAGTGGAAGCCCCTACAGCTGGACCGACCACTCCCAACGGGAACTTGGTGGATGAATGTGATGACGACCAGGCCAACTGCCACAGTGGAACAGGTGATGACTTCCAGCTCACAGGTGGCACCACTGTGCTGGCCACAGAAAAGCCCACGGTCATAGACAGCACCATACAATCAGAGTTTCCAACATATGGTTTTAACTGTGAATTTGGCTGGGGCTCTCACAAGACCTTCTGCCACTGGGAACATGACAATCACGTGCAGCTCAAGTGGAGTGTGTTGACCAGCAAGACGGGACCCATTCAGGATCACACAGGAGATGGCAACTTCATCTATTCCCAAGCTGACGAAAATCAGAAGGGCAAAGTGGCTCGCCTGGTGAGCCCTGTGGTTTATTCCCAGAACTCTGCCCACTGCATGACCTTCTGGTATCACATGTCTGGGTCCCACGTCGGCACACTCAGGGTCAAACTGCGCTACCAGAAGCCAGAGGAGTACGATCAGCTGGTCTGGATGGCCATTGGACACCAAGGTGACCACTGGAAGGAAGGGCGTGTCTTGCTCCACAAGTCTCTGAAACTTTATCAGGTGATTTTCGAGGGCGAAATCGGAAAAGGAAACCTTGGTGGGATTGCTGTGGATGACATTAGTATTAATAACCACATTTCACAAGAAGATTGTGCAAAACCAGCAGACCTGGATAAAAAGAACCCAGAAATTAAAATTGATGAAACAGGGAGCACGCCAGGATACGAAGGTGAAGGAGAAGGTGAC  AAGAACATCTCCAGGAAGCCAGGCAATGTGTTGAAGACCTTAGACCCCATCCTCATCACCATCATAGCCATGAGTGCCCTGGGGGTCCTCCTGGGGGCTGTCTGTGGGGTCGTGCTGTACTGTGCCTGTTGGCATAATGGGATGTCAGAAAGAAACTTGTCTGCCCTGGAGAACTATAACTTTGAACTTGTGGATGGTGTGAAGTTGAAAAAAGACAAACTGAATACACAGAGTACTTATTCGGAGGCA |
| --- |

**Table S6: Sequences of NRP1 and peptides for molecular docking.**

| NRP1 | GSHMFKRGPECSQNYTTPSGVIKSPGFPEKYPNSLECTYIVFAPKMSEIILEFESFDLEPDSNPPGGMFCRYDRLEIWDGFPDVGPHIGRYCGQKTPGRIRSSSGILSMVFYTDSAIAKEGFSANYSVLQSSVSEDFKCMEALGMESGEIHSDQITASSQYSTNWSAERSRLNYPENGWTPGEDSYREWIQVDLGLLRFVTAVGTQGAISKETKKKYYVKTYKIDVSSNGEDWITIKEGNKPVLFQGNTNPTDVVVAVFPKPLITRFVRIKPATWETGISMRFEVYGCKITDYPCSGMLGMVSGLISDSQITSSNQGDRNWMPENIRLVTSRSGWALPPAPHSYINEWLQIDLGEEKIVRGIIIQGGKHRENKVFMRKFKIGYSNNGSDWKMIMDDSKRKAKSFEGNNNYDTPELRTFPALSTRFIRIYPERATHGGLGLRMELLGCEV |
| --- | --- |
| RP7 | RPARPAR |
| Tat-C-RP7 | RKKRRQRRRCRPARPAR |

**Table S7: Gene list for GOBP-EMT**

| ACVR1 | FLNA | NOG | SPRED3 | EMP2 |
| --- | --- | --- | --- | --- |
| ACVRL1 | FOXC1 | NOLC1 | SPRY1 | ENG |
| ADAM15 | FOXF2 | NOTCH1 | SPRY2 | EOMES |
| ADAM8 | FUZ | NOTCH4 | TBX3 | EPB41L5 |
| ADIPOR1 | GATA3 | OLFM1 | TBX5 | EPHA3 |
| AGT | GCNT2 | OVOL2 | TCF7L2 | EPHA4 |
| AKNA | GLIPR2 | PDCD4 | TCOF1 | EZH2 |
| ALX1 | GREM1 | PDCD6 | TGFB1 | FAM83D |
| APLF | GSC | PDPN | TGFB1I1 | FERMT2 |
| AXIN2 | GSK3B | PEF1 | TGFB2 | FGFR1 |
| BAMBI | HAS2 | PHLDB1 | TGFB3 | FGFR2 |
| BCL9L | HDAC2 | PHLDB2 | TGFBR1 | LOXL3 |
| BMP2 | HEY1 | POFUT2 | TGFBR2 | LRG1 |
| BMP4 | HEY2 | POLR1B | TGFBR3 | LRP6 |
| BMP7 | HEYL | PPP2CA | TGFBR3L | MAD2L2 |
| CLASP1 | HGF | PPP3R1 | TIAM1 | MARK1 |
| CLASP2 | HIF1A | PTEN | TMEM100 | MCRIP1 |
| COL1A1 | HMGA2 | PTK2 | TNXB | MDK |
| CRB2 | HNRNPAB | QKI | TRIM28 | MSX1 |
| CTNNB1 | IGF1 | RBPJ | TRIM62 | MSX2 |
| CUL7 | IL17RD | RFLNB | TWIST1 | MTOR |
| DAB2 | IL1B | RGCC | USF3 | NCAM1 |
| DAB2IP | IL6 | ROCK1 | VASN | SLC39A6 |
| DACT3 | JAG1 | ROCK2 | VEGFA | SMAD2 |
| DAG1 | KAT8 | RTN4 | WNT11 | SMAD3 |
| DDX17 | KBTBD8 | S100A4 | WNT16 | SMAD4 |
| DDX5 | KDM1A | SDCBP | WNT2 | SMAD7 |
| DLG5 | KLHL12 | SDHAF2 | WNT4 | SNAI1 |
| EDN1 | LDLRAD4 | SERPINB3 | WNT5A | SNAI2 |
| EDNRA | LEF1 | SFRP1 | WWTR1 | SOX9 |
| EFNA1 | LIMS1 | SFRP2 | ZNF703 | SP6 |
| ELL3 | LOXL2 | SLC39A10 | ZNF750 | SPRED1 |
| SPRED2 |  |  |  |  |

**Table S8: Gene list for HALL-EMT**

| ABI3BP | CXCL6 | IGFBP3 | NT5E | SPOCK1 |
| --- | --- | --- | --- | --- |
| ACTA2 | CXCL8 | IGFBP4 | NTM | SPP1 |
| ADAM12 | DAB2 | IL15 | OXTR | TAGLN |
| ANPEP | DCN | IL32 | P3H1 | TFPI2 |
| APLP1 | DKK1 | IL6 | PCOLCE | TGFB1 |
| AREG | DPYSL3 | INHBA | PCOLCE2 | TGFBI |
| BASP1 | DST | ITGA2 | PDGFRB | TGFBR3 |
| BDNF | ECM1 | ITGA5 | PDLIM4 | TGM2 |
| BGN | ECM2 | ITGAV | PFN2 | THBS1 |
| BMP1 | EDIL3 | ITGB1 | PLAUR | THBS2 |
| CADM1 | EFEMP2 | ITGB3 | PLOD1 | THY1 |
| CALD1 | ELN | ITGB5 | PLOD2 | TIMP1 |
| CALU | EMP3 | JUN | PLOD3 | TIMP3 |
| CAP2 | ENO2 | LAMA1 | PMEPA1 | TNC |
| CAPG | FAP | LAMA2 | PMP22 | TNFAIP3 |
| CD44 | FAS | LAMA3 | POSTN | TNFRSF11B |
| CD59 | FBLN1 | LAMC1 | PPIB | TNFRSF12A |
| CDH11 | FBLN2 | LAMC2 | PRRX1 | TPM1 |
| CDH2 | FBLN5 | LGALS1 | PRSS2 | TPM2 |
| CDH6 | FBN1 | LOX | PTHLH | TPM4 |
| COL11A1 | FBN2 | LOXL1 | PTX3 | VCAM1 |
| COL12A1 | FERMT2 | LOXL2 | PVR | VCAN |
| COL16A1 | FGF2 | LRP1 | QSOX1 | VEGFA |
| COL1A1 | FLNA | LRRC15 | RGS4 | VEGFC |
| COL1A2 | FMOD | LUM | RHOB | VIM |
| COL3A1 | FN1 | MAGEE1 | SAT1 | WIPF1 |
| COL4A1 | FOXC2 | MATN2 | SCG2 | WNT5A |
| COL4A2 | FSTL1 | MATN3 | SDC1 | SPARC |
| COL5A1 | FSTL3 | MCM7 | SDC4 | NOTCH2 |
| COL5A2 | FUCA1 | MEST | SERPINE1 | IGFBP2 |
| COL5A3 | FZD8 | MFAP5 | SERPINE2 | SLIT3 |
| COL6A2 | GADD45A | MGP | SERPINH1 | SNAI2 |
| COL6A3 | GADD45B | MMP1 | SFRP1 | SNTB1 |
| COL7A1 | GAS1 | MMP14 | SFRP4 | MYLK |
| COL8A2 | GEM | MMP2 | SGCB | NID2 |
| COLGALT1 | GJA1 | MMP3 | SGCD | NNMT |
| COMP | GLIPR1 | MSX1 | SGCG | GREM1 |
| COPA | GPC1 | MXRA5 | SLC6A8 | HTRA1 |
| CRLF1 | GPX7 | MYL9 | SLIT2 | ID2 |
| CTHRC1 | CXCL1 | CXCL12 |  |  |

**Table S9: Gene list for Foroutan_TGFB_UP**

| ABCA1 | DLC1 | KDELR3 | PSMD2 | TNFAIP6 |
| --- | --- | --- | --- | --- |
| ACKR3 | DOCK4 | KLF7 | PTHLH | TNS1 |
| ACTN1 | DSE | LAMC2 | PTPN21 | TP53I3 |
| ADAM12 | DUSP10 | LARP6 | PTPRK | TPM1 |
| ADAM19 | ELK3 | LBH | PXDC1 | TPM4 |
| ADAMTS6 | EML1 | LMCD1 | RALA | TPST1 |
| AKT3 | EPHB2 | LOX | RFTN1 | TPST2 |
| ALOX5AP | ETS2 | LUM | RGS4 | TUBA1A |
| AMIGO2 | FAM114A1 | MAF | RUNX2 | TUBA4A |
| ANGPTL4 | FBN1 | MAGED2 | SACS | TUFT1 |
| ANKLE2 | FERMT2 | MAP1LC3B | SCG2 | VCAN |
| AP1S2 | FHOD3 | MATN3 | SCG5 | VEGFC |
| APBB2 | FN1 | MBOAT2 | SEMA3C | VGLL3 |
| ARFGAP1 | FOXD1 | MFAP2 | SERPINE1 | VIM |
| ARHGEF40 | FSTL3 | MICAL2 | SERPINE2 | WNT5A |
| BHLHE40 | GADD45B | MMP1 | SIK1 | WNT5B |
| BMP1 | GAL | MMP10 | SKIL | XYLT1 |
| BMP2 | GALNT10 | MMP2 | SLC22A4 | ZNF365 |
| BMPR2 | GFPT2 | MMP9 | SLC26A2 | KCNMA1 |
| BPGM | GLIPR1 | MN1 | SLCO2A1 | PRR5L |
| C3orf52 | GNG11 | MRC2 | SLN | TMCC1 |
| CALD1 | GRB10 | MYL9 | SMAD7 | TGM2 |
| CD59 | GREM1 | MYO10 | SMURF2 | THBS1 |
| CDH11 | HMGA2 | NCF2 | SNAI2 | TIMP2 |
| CDH2 | HMOX1 | NEDD9 | SPARC | PMEPA1 |
| CDK14 | HRH1 | NKX3-1 | SPDL1 | PODXL |
| CHRNA9 | HS3ST3A1 | NREP | SPHK1 | POSTN |
| CHST11 | HS3ST3B1 | NRIP3 | SPOCK1 | JUN |
| COL1A1 | HSF2BP | NT5E | SRPX | JUNB |
| COL3A1 | HTRA1 | NUAK1 | SRRD | KCNJ15 |
| COL4A1 | IGFBP5 | PALLD | STC1 | DACT1 |
| COL4A2 | IGFBP7 | PDGFA | TAGLN | DHRS2 |
| COL5A1 | IL11 | PDGFC | TAGLN2 | DIXDC1 |
| COL5A2 | INHBA | PDLIM7 | TBX3 | TGFBI |
| COL6A3 | INPP4B | PEA15 | TCF4 | PLEK2 |
| COL7A1 | ITGA5 | PID1 | TFPI2 | JARID2 |
| CRLF1 | ITGB3 | PIK3CD | TGFB1 | DAAM1 |
| CYTH1 | JAG1 | PLAUR | TGFB1I1 |  |

**Table S10: Gene list for TGFB_receptor_pathway**

| ACVR1 | ENG | ITGB8 | RBBP4 | TGFB3 |
| --- | --- | --- | --- | --- |
| ACVRL1 | EP300 | JUN | RBBP7 | TGFBR1 |
| ADAM17 | FAM89B | LATS1 | RNF111 | TGFBR2 |
| ADAM9 | FBN1 | LATS2 | SAP130 | TGFBR3 |
| ADAMTSL2 | FBN2 | LDLRAD4 | SAP30 | TGFBR3L |
| AMHR2 | FERMT1 | LEFTY1 | SAP30L | TGFBRAP1 |
| APOA1 | FERMT2 | LEMD3 | SDCBP | THBS1 |
| APPL1 | FKBP1A | LOX | SIN3A | TP53 |
| APPL2 | FLCN | LPXN | SINHCAF | TRIM33 |
| ARID4A | FMOD | LRG1 | SIRT1 | TSC22D1 |
| ARID4B | FNTA | LRRC32 | SKI | TWSG1 |
| ARRB2 | FOLR1 | LTBP1 | SKIL | USP15 |
| ASPN | FOS | LTBP2 | SLC2A10 | USP9X |
| AXIN1 | FURIN | LTBP3 | SMAD1 | USP9Y |
| BAMBI | FUT8 | LTBP4 | SMAD2 | VASN |
| BCL9 | GCNT2 | MAP3K7 | SMAD3 | VEPH1 |
| BCL9L | GDF10 | MEN1 | SMAD4 | WFIKKN1 |
| BMP2 | GDF15 | MSTN | SMAD5 | WFIKKN2 |
| BMPR1A | GDF5 | MTMR4 | SMAD6 | ZBTB7A |
| BRMS1 | GDF9 | MYOCD | SMAD7 | ZEB1 |
| BRMS1L | GIPC1 | NDP | SMAD9 | ZEB2 |
| CAV2 | GLG1 | NLK | SMURF1 | ZFYVE9 |
| CAV3 | HDAC1 | NPNT | SMURF2 | ZMIZ1 |
| CD109 | HDAC2 | NREP | SNW1 | ZMIZ2 |
| CDH3 | HIPK2 | NRROS | SNX25 | ZNF451 |
| CDH5 | HPGD | OGT | SNX6 | ZNF703 |
| CDKN1C | HSP90AB1 | ONECUT2 | SOX11 | ZYX |
| CDKN2B | HSPA1A | OVOL2 | SPRED1 | ITGB6 |
| CHST11 | HSPA5 | PARP1 | SPRED2 | RASL11B |
| CIDEA | HTRA1 | PBLD | SPRED3 | TGFB2 |
| CILP | HTRA3 | PDPK1 | SPRY1 | TGFB1 |
| CITED1 | HTRA4 | PEG10 | SPRY2 | TGFB1I1 |
| CITED2 | ID1 | PIN1 | SRC | PTPRK |
| CLDN5 | IL17F | PMEPA1 | STAT3 | PXN |
| COL1A2 | IL17RD | PML | STK11 | ITGB1 |
| COL3A1 | ING1 | PPARA | STRAP | ITGB5 |
| CREBBP | ING2 | PPARG | STUB1 | DUSP22 |
| DAB2 | INTS9 | PPM1A | SUDS3 | EID2 |
| DAND5 | ITGA3 | PRDM16 | TAB1 | TET1 |
| DKK3 | ITGA8 | PTK2 |  |  |

**Table S11: Gene list for TGFB_receptor_binding**

| AMH | TGFBR2 | SNX6 | SMAD2 | LEFTY1 |
| --- | --- | --- | --- | --- |
| BAMBI | TGFBR3 | TGFB1 | SMAD3 | LEFTY2 |
| ENG | TGFBR3L | TGFB2 | SMAD6 | LRG1 |
| FERMT2 | TGFBRAP1 | TGFB3 | SMAD7 | MAP3K7 |
| FKBP1A | USP15 | TGFBR1 | SMURF1 | RASL11B |

**Table S12: Gene list for HALL_TGFB**

| ACVR1 | TGFBR1 | SMAD1 | PPP1CA | KLF10 |
| --- | --- | --- | --- | --- |
| APC | TGIF1 | SMAD3 | PPP1R15A | LEFTY2 |
| ARID4B | THBS1 | SMAD6 | RAB31 | LTBP2 |
| BCAR3 | TJP1 | SMAD7 | RHOA | MAP3K7 |
| BMP2 | TRIM33 | SMURF1 | SERPINE1 | NCOR2 |
| BMPR1A | UBE2D3 | SMURF2 | SKI | NOG |
| BMPR2 | WWTR1 | SPTBN1 | SKIL | PMEPA1 |
| CDH1 | XIAP | TGFB1 | SLC20A1 | PPM1A |
| CDK9 | ENG | FURIN | ID1 | IFNGR2 |
| CDKN1C | FKBP1A | HDAC1 | ID2 | JUNB |
| CTNNB1 | FNTA | HIPK2 | ID3 |  |

**Table S13: Gene list for TGFB_receptor_activates_smads**

| BAMBI | UBB | TGFB1 | SMAD3 | PMEPA1 |
| --- | --- | --- | --- | --- |
| CBL | UBC | TGFB2 | SMAD4 | PPP1CA |
| FBN1 | UBE2M | TGFB3 | SMAD7 | PPP1CB |
| FKBP1A | UCHL5 | TGFBR1 | SMURF1 | PPP1CC |
| FURIN | USP15 | TGFBR2 | SMURF2 | PPP1R15A |
| ITGA8 | XPO1 | TGFBR3 | STRAP | RPS27A |
| ITGAV | ZFYVE9 | UBA52 | STUB1 | SMAD2 |
| ITGB1 | MTMR4 | LTBP2 | ITGB6 | LTBP1 |
| ITGB3 | NEDD4L | LTBP3 | ITGB8 | LTBP4 |
| ITGB5 | NEDD8 |  |  |  |

**Table S14: Gene list for TGFB_in_EMT**

| ARHGEF18 | UBA52 | TGFB1 | RHOA | PARD3 |
| --- | --- | --- | --- | --- |
| CGN | UBB | TGFBR1 | RPS27A | PARD6A |
| F11R | UBC | TGFBR2 | SMURF1 | PRKCZ |
| FKBP1A |  |  |  |  |

**Table S15: Gene list for Regulation_TGFB**

| CDKN1C | CITED2 | ENG | PEG10 | SMAD4 |
| --- | --- | --- | --- | --- |
| CDKN2B | EID2 | HIPK2 | SMAD2 | SNX6 |
| CIDEA | TGFB1 | IL17F | SMAD3 |  |
